# Analysis of Genetic Diversity in the Traditional Chinese Medicine Plant ‘Kushen’ (*Sophora flavescens* Ait.)

**DOI:** 10.3389/fpls.2021.704201

**Published:** 2021-08-03

**Authors:** Carolyn J. Schultz, Shashi N. Goonetilleke, Jianping Liang, Jelle Lahnstein, Kara A. Levin, Tina Bianco-Miotto, Rachel A. Burton, Diane E. Mather, Kenneth J. Chalmers

**Affiliations:** ^1^School of Agriculture, Food and Wine, Waite Research Institute, University of Adelaide, Adelaide, SA, Australia; ^2^Department of Chinese Medicine, College of Life Sciences, Shanxi Agricultural University, Shanxi, China

**Keywords:** quinolizidine alkaloids, traditional Chinese medicine, DArT sequencing, lysine decarboxylase, copper amine oxidase, single nucleotide polymorphisms, gene duplication, genetic diversity

## Abstract

Kushen root, from the woody legume *Sophora flavescens*, is a traditional Chinese medicine that is a key ingredient in several promising cancer treatments. This activity is attributed in part to two quinolizidine alkaloids (QAs), oxymatrine and matrine, that have a variety of therapeutic activities *in vitro*. Genetic selection is needed to adapt *S. flavescens* for cultivation and to improve productivity and product quality. Genetic diversity of *S. flavescens* was investigated using genotyping-by-sequencing (GBS) on 85 plants grown from seeds collected from 9 provinces of China. DArTSeq provided over 10,000 single nucleotide polymorphism (SNP) markers, 1636 of which were used in phylogenetic analysis to reveal clear regional differences for *S. flavescens*. One accession from each region was selected for PCR-sequencing to identify gene-specific SNPs in the first two QA pathway genes, lysine decarboxylase (LDC) and copper amine oxidase (CAO). To obtain *SfCAO* sequence for primer design we used a targeted transcript capture and assembly strategy using publicly available RNA sequencing data. Partial gene sequence analysis of *SfCAO* revealed two recently duplicated genes, *SfCAO1* and *SfCAO2*, in contrast to the single gene found in the QA-producing legume *Lupinus angustifolius*. We demonstrate high efficiency converting SNPs to Kompetitive Allele Specific PCR (KASP) markers developing 27 new KASP markers, 17 from DArTSeq data, 7 for *SfLDC*, and 3 for *SfCAO1*. To complement this genetic diversity analysis a field trial site has been established in South Australia, providing access to diverse *S. flavescens* material for morphological, transcriptomic, and QA metabolite analysis. Analysis of dissected flower buds revealed that anthesis occurs before buds fully open suggesting a potential for *S. flavescens* to be an inbreeding species, however this is not supported by the relatively high level of heterozygosity observed. Two plants from the field trial site were analysed by quantitative real-time PCR and levels of matrine and oxymatrine were assessed in a variety of tissues. We are now in a strong position to select diverse plants for crosses to accelerate the process of genetic selection needed to adapt kushen to cultivation and improve productivity and product quality.

## Introduction

Extracts from roots of the woody deciduous legume kushen (*Sophora flavescens* Ait.) have long been used in traditional Chinese medicine. The medicinal use of kushen root preparations was first documented in 200 A.D. in the Chinese book *Shen Nong Ben Cao Jing*^1^ and continues today for treatment of a variety of conditions, including cancers, hepatitis, and heart disease (Sun et al., 2012; He et al., 2015). Of particular importance is the use of kushen extracts in combination with extracts from *Smilax glabra* in compound kushen injection (CKI). CKI has been used in Chinese hospitals to treat cancer for over 20 years (Cui et al., 2020) and has been reported to boost immunity, decrease inflammation, and decrease metastasis (Wang et al., 2015).

More than 200 compounds have been isolated from kushen, including alkaloids, flavonoids, triterpenoids, and phenolics (He et al., 2015). Among these, the quinolizidine alkaloids (QAs) matrine and oxymatrine have been the main focus of pharmacological studies (Chen et al., 2004; He et al., 2015; Zhang et al., 2020). QA alkaloids are also produced by a subset of legumes, including narrow-leaf lupin (*Lupinus angustifolius*), and they are responsible for the bitter taste of some lupin varieties (Ohmiya et al., 1995; Bunsupa et al., 2016; Carvajal-Larenas et al., 2016). QA alkaloids are synthesised in leaves, stems, and seedpods, then transported to roots and seeds, where they are thought to provide protection from biotic attack (Lee et al., 2007; Otterbach et al., 2019). Levels of matrine and oxymatrine have been reported to be three to four times higher in kushen root and seed tissue than in leaf and stem tissue (Zhang et al., 2020).

**FIGURE 1:**
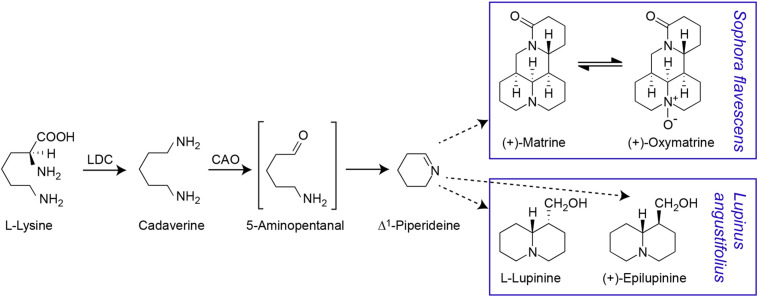
Proposed quinolizidine alkaloid biosynthesis pathway. The first two steps in the pathway have been confirmed. The gene for the first step, the conversion of L-lysine to cadaverine by a bifunctional lysine/ornithine decarboxylase (L/ODC), has been cloned from a variety of species including *S. flavescens* (Bunsupa et al., 2012). The conversion of cadaverine to 5-aminopentanal, requires a copper amino oxidase (CAO) and this intermediate is assumed to be spontaneously cyclised to Δ^1^-piperideine. The CAO gene involved in QA biosynthesis has been cloned from *L. angustifolius* (Yang et al., 2017) but not from *S. flavescens*. Δ^1^-piperideine is the precursor for different QAs in *S. flavescens* [including (+)-matrine and (+)-oxymatrine) and *L. angustifolius* (including (–)-lupinine and (+)-epilupinine] (Ohmiya et al., 1995; Sato et al., 2018). Dashed arrows indicate multiple reaction steps.

Consistent with early evidence that matrine can be synthesised from lysine, cadaverine, and Δ^1^-piperideine (Ohmiya et al., 1995), it is now known that the QA biosynthesis pathway begins with conversion of lysine to cadaverine, followed by oxidation of cadaverine to 5-aminopentanal, conversion of 5-aminopentanal to Δ^1^-piperideine and conversion of Δ^1^-piperideine to a piperideine dimer (Figure 1). The first step is catalysed by a bifunctional lysine/ornithine decarboxylase [L/ODC, hereafter lysine decarboxylase (LDC)], which is thought to have evolved from ornithine decarboxylase (ODC), enabling the production of lysine-derived alkaloids (Bunsupa et al., 2012, Bunsupa et al., 2016). As these alkaloids occur in legumes, basal angiosperms, and lycophytes (club mosses), the evolution of LDC from ODC is thought to have occurred independently in three phylogenetic clades of plants (Bunsupa et al., 2016). In legumes, LDC has been shown to be localised in the chloroplast, where lysine biosynthesis occurs (Wink and Hartmann, 1982). The oxidation of cadaverine to 5-aminopentanal is catalysed by a cadaverine oxidase/copper amine oxidase (CAO) (Yang et al., 2017). CAO seems to be a peroxisomal protein, indicating a need for intermediates in the QA pathway to be shuffled between subcellular compartments (Yang et al., 2017). The conversion of 5-aminopentanal to Δ^1^-piperideine is thought to occur spontaneously (Sato et al., 2018, 2019). The remaining steps in the QA pathway remain unknown. While transcriptional analysis in lupin has identified numerous genes that may be involved in QA synthesis, pathway regulation or transport, these candidate genes remain to be fully analysed and confirmed (Yang et al., 2017; Kroc et al., 2019).

In *L. angustifolius*, the genes encoding LDC (*LaLDC*) and CAO (*LaCAO*) have been isolated and found to be expressed in leaves and stems (Bunsupa et al., 2012; Yang et al., 2017). When *LaLDC* was overexpressed in *Arabidopsis thaliana* (which does not normally make cadaverine), both cadaverine and cadaverine-derived alkaloids were produced (Shimizu et al., 2019). In *S. flavescens*, an LDC gene (*SfLDC*) has been isolated and reported to be most abundantly expressed in leaf and stem tissues, with little or no expression detected in inflorescence tissues (pedicels, flower buds, and flowers) or in callus (Han et al., 2015).

Traditionally, kushen roots were harvested from wild populations in China, but with increasing demand for the product, kushen began to be cultivated, especially in the northwestern provinces Shanxi, Hubei, Henan, and Hebei (Weng et al., 2017). Agricultural practices for this new crop remain to be optimised. For example, it has been suggested that seed might be used instead of roots as a source of matrine and oxymatrine (Weng et al., 2017). Further, there may be a need for genetic selection to adapt kushen to cultivation and to improve productivity and product quality. Relatively little is known about the extent of genetic and phenotypic diversity in *S. flavescens*. In a study of plants from four locations (three in China and one in Taiwan), Lin et al. (2014) established that the plants were diploid (2*n* = 18) and observed statistically significant variation in biochemical composition (matrine, oxymatrine, flavonoids, and antioxidants) and 11 morphological traits among field-grown plants.

In the research reported here, genetic diversity of kushen was investigated by applying GBS to 85 accessions of *S. flavescens* obtained from nine provinces of China. Further, to extend understanding of QA synthesis in kushen, a full-length *SfCAO* coding sequence was isolated from publicly available transcriptome data. A kushen field nursery was established in southern Australia and the expression of *SfLDC* and *SfCAO* was investigated in seed-grown plants from that nursery.

## Materials and Methods

### Growth and Collection of Plant Material

**FIGURE 2:**
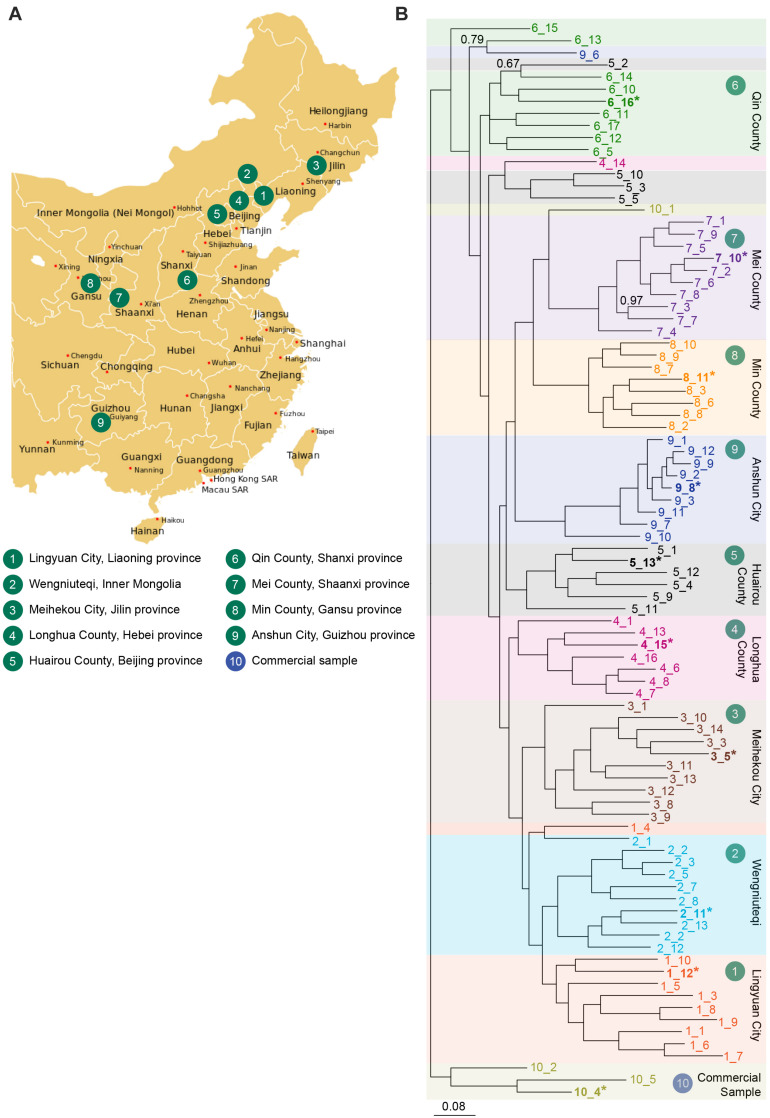
**(A)** Collection sites for *S. flavescens* seeds spanning the natural range of the species and representing nine different regions in China. A commercial seed sample was obtained from Internet retailer Alibaba. Regions 4–8 coincide with the silk road (Wang et al., 2019). **(B)** Phylogenetic analysis of SNP data generated using DArTSeq using a Bayesian inference method in MrBayes v3.2.6. A total of 89 samples were analysed, 85 samples grown from seeds collected from 9 provinces of China (10 accessions from most regions, except region 4, 6, and 8, with 8, 9, and 8 accessions, respectively), and 4 samples from commercial seed. Accessions from each population are labelled in a different colour, and are indicated by region number_sample number (e.g., 1_12). Samples in bold and indicated by an asterisk were used to sequence *SfLDC* and *SfCAO* gene fragments. A total of 1636 SNPs was used (those with less than 5% missing data and minor allele frequencies greater than 5%). Values reported on the nodes are Bayesian posterior probabilities and are 1.0 unless otherwise indicated. Scale bar represents the probability of nucleotide substitutions per site. Map image: https://upload.wikimedia.org/wikipedia/commons/d/d2/China_administrative_claimed_included.svg.

Seeds of *S. flavescens* were obtained from a collection maintained at Shanxi Agricultural University. They were collected from non-cultivated (natural) habitats in nine different provinces in China (counting inner Mongolia as a province), representing the natural distribution of the species. Seed were collected from a single region per province, ranging from Meihekou City, Jilin province in the northeast of China to Anshun City, Guizhou province in the southwest of China (Figure 2A). In each province, seed was collected from multiple plants at a single site and the seed pooled. A subsample of seeds from each population was imported into Australia. A “commercial” sample of *S. flavescens* seeds was also purchased over the Internet.^2^ Seeds were sown in 15 cm pots filled with coco-peat and supplied with slow-release fertiliser granules Scotts Osmocote^®^ Plus Trace Elements Landscape Formula (Scott’s Australia, Bella Vista, Australia), in a glasshouse under 12 h daylength. Young leaf tissue was harvested from up to 10 seedlings from each region for DNA analysis. Additional seedlings were transferred to a field trial site at the Waite Campus of the University of Adelaide, with five to seven plants per row. An initial planting was done in October 2017, and a second planting was done in October 2018, to replace plants that had died. Plants were drip irrigated daily in summer. Tissues for RNA extraction were harvested on 26 November 2018, except for seed and seedpod tissues, that were harvested on 12 February 2019.

### Genomic DNA Extraction for DArT Sequencing

Genomic DNA was extracted from freeze dried young leaf tissue using a modified cetyltrimethylammonium bromide (CTAB) protocol (Doyle, 1987), that was optimised for yield and quality in 2 mL tubes. DNA was sent to Diversity Arrays Technology (Bruce, ACT, Australia) for GBS using the DArTseq platform.^3^ Markers scores were provided as single-nucleotide polymorphisms (SNPs) (pairs of sequence tags differing only by 1 nt) or presence/absence polymorphisms detected in some accessions but not others. Two rounds of DArTseq were performed, a preliminary round on four samples and a full round on 89 samples.

### Identification of Cadaverine/Copper-Containing Amine Oxidase Transcripts From RNAseq Datasets

To obtain a full length cDNA sequence for *SfCAO*, mirabait transcript capture (Chevreux et al., 2004) was used to identify short reads that were subsequently assembled in Geneious (8.1.9) as follows. Two RNAseq datasets (DRR031281 and DRR031283) were selected from the nine datasets publicly available for *S. flavescens* (project PRJDB3906) (Han et al., 2015) as they had the highest number of sequences matching the QA pathway genes *SfLDC* (AB561138.1) and *LaCAO* (MF152953) (BLASTn, no filtering, word size *n* = 7, data not shown). The datasets were trimmed and error corrected using Quorum, version 1.1.1 (Marçais et al., 2015) or Trim Galore, version 0.4.2 (Krueger, 2015). Housekeeping genes were also obtained for designing PCR primers using *L. angustifolius* sequences as bait and the *S. flavescens* sequence, *SfLDC* (AB561138.1), was used as a positive control *Sophora* for the mirabait short-read capture method. The *L. angustifolius* bait sequences [full coding sequence plus up to 20 base pairs (bp) of the 5′ and 3′ untranslated regions (UTRs) (if available)] were as follows: *LaCAO* (MF152953), tubulin (XM_019596948.1), cyclophilin (XM_019595391), elongation faction-α (XM_019597475.1), and ubiquitin-60S ribosomal protein (XM_019577360.1). The first round of mirabait (mirabait1) used low stringency parameters {-k 21 [k = *k*-mer-length (default = 31)]; and –n 2 [n = minimum number of *k*-mers needed for a sequence to be selected (default = 1)]}. Sequences were assembled in Geneious and contigs evaluated by BLASTn to identify the best sequences. A second round of mirabait was performed (mirabait2) at high stringency (default parameters) using the best contigs from mirabait1, short reads assembled in Geneious, evaluated by BLASTn and the best contigs used for further analysis.

### Phylogenetic Analysis

Phylogenetic analysis was performed using the DArTSeq SNP data generated on the 89 DNA samples using MrBayes v3.2.6 (Huelsenbeck and Ronquist, 2001) with the following parameters: a general time reversible model and a gamma shaped distribution of rates across sites function were used. The Markov Chain Monte Carlo (MCMC) was set to two million generations with sample frequency of 100 and 5000 burn-in. The analysis used all SNPs with less than 5% missing data and for which the minor allele frequency was at least 5%. The resulting phylogenetic tree was visualised with FigTree v.1.4.4 (Rambaut, 2018).

Phylogenetic analysis of CAO proteins was performed using a maximum likelihood method with MEGA 10.0.5 (Kumar et al., 2018). Sequences (untrimmed) were first aligned using MUSCLE (Edgar, 2004) using the default settings. The best model (JTT + G) was identified using the find best protein model (ML) option and used to generate a maximum likelihood tree (with 1000 bootstrap replications) using all sites in the alignment.

### DNA Sequencing of Genic SfLDC and SfCAO PCR Products

To identify SNPs in the two QA pathway genes, *SfLDC* and *SfCAO* PCR products were amplified from genomic DNA using Phusion^TM^ High-Fidelity DNA Polymerase (ThermoFisher, Australia). *SfLDC* (full length) gene sequences were amplified using SfLDC_F_5pr and SfLDC_R1. Two partial gene fragments were amplified for *SfCAO*: a 5′ fragment using SfCAO_5p_Fd and SfCAO_e1_R, and a 3′ fragment SfCAO_e10_F and SfCAO_R1. Primer information is listed in Supplementary Table 1. PCR reactions were prepared (50 μL) using 100 ng of genomic DNA. Thermocycling conditions were: 98°C for 30 s, followed by 30 cycles of 98°C for 10 s, annealing at 65°C for 30 s, with extension at 72°C for 15–25 s per kilobase (kb) of sequence, with a final extension at 72°C for 5 min. For accessions where standard PCR was unsuccessful, presumably due to divergent sequence at one or more primer binding sites, touchdown PCR was used (Korbie and Mattick, 2008) with modified cycling conditions after the initial denaturing, Phase 1 (15 cycles, with annealing temperature starting at 66°C and reducing 0.5°C per cycle), followed by Phase 2 (20 cycles, with annealing at 59°C).

For Sanger sequencing,^4^ PCR products were purified using the NucleoSpin gel kit from Macherey-Nagel (Bethlehem, PA, United States), or by gel isolation when two or more PCR products were produced (see section “Results”), as per manufacturer’s instructions. PCR products were sequenced using both forward and reverse primers. Sequencing chromatograms were analysed using Geneious 8.1.9. The following additional primers were used to sequence the genomic fragments: SfLDC_F314, SfLDC_F752, SfLDC_R596, SfLDC_R1026 and in some cases SfCAO_e11_F and SfCAO_e11_R (Supplementary Table 1).

### Development of KASP Markers

Kompetitive Allele Specific PCR (KASP) uniplex assays (hereafter KASP markers) (He et al., 2014) were developed and tested using the sequences derived from 19 randomly selected DArTseq-derived GBS tags and two QA pathway genes, *SfLDC* and *SfCAO1*. Primer sets were designed using Kraken^TM^ software (LGC Limited, Teddington, United Kingdom). The KASP markers for GBS-derived SNPs were assayed on the four DNA samples that were used in the preliminary round of GBS analysis. The KASP markers for SNPs in *SfLDC* and *SfCAO1* were assayed on 86 of the DNA samples used in the second round of GBS analysis. KASP genotyping was conducted using an automated SNPLine system (LGC Limited, Teddington, United Kingdom).

### RNA Extraction, cDNA Synthesis, and Quantitative Real-Time PCR

Tissues for RNA were harvested, immediately frozen in liquid nitrogen and stored at −80°C until required. Tissues (leaf, stem, flower buds, and open flowers and roots) were sampled from two field grown plants, 1_LC1 [Lingyuan City (LC), Liaoning Province] and 9_AC1 [Anshun City (AC), Guizhou Province] in triplicate, without destroying the plants (Supplementary Figures 1, 2). Seedpods containing seeds were collected from another plant, 1_LC4 (Supplementary Figure 3). Frozen tissue was ground to a fine powder in the presence of liquid nitrogen, either in a mortar and pestle or an IKA A11 grinder [IKA^®^ Works (Asia) Sdn Bhd, Selangor, Malaysia]. Total RNA was extracted from 100 mg of frozen ground tissue using the Spectrum^TM^ Plant Total RNA Kit according to manufacturer’s instructions. Total RNA was treated with DNase I (New England Biolabs, M0303S, Ipswich, MA, United States), then 750 ng of RNA was used to prepare cDNA using Superscript^TM^ III reverse transcriptase (ThermoFisher) using oligodT_18_ as primer (50°C for 1 h).

Transcript profiling of *SfLDC* and *SfCAO* was carried out with all cDNA samples using quantitative real-time PCR (qPCR) methods as described (Burton et al., 2011), and gene-specific primers listed in Supplementary Table 1. Data was normalised against the geometric mean of the four control genes: cyclophilin (CYC), tubulin (TUB), elongation factor-α (EFa), and ubiquitin-60S ribosomal protein (UBQ) (Burton et al., 2011).

Full length *SfCAO* cDNA sequences were obtained by PCR from one of three stem cDNA samples each for 1_LC and 9_AC. PCR was performed with Phusion as described above except that a longer extension (60 s) was used with primers SfCAO_5p_Fd and SfCAO_R1. PCR products were sequenced (Sanger) with the PCR primers and additional internal primers: SfCAO_e5_F, SfCAO_ex8_F, SfCAO_ex7_R, and SfCAO_ex4_R (Supplementary Table 1).

### Extraction and Quantification of Oxymatrine and Matrine

Protocols for extraction of oxymatrine and matrine from *S. flavescens* tissues and a reversed-phase high performance liquid chromatography (HPLC) quantification method were based on the Hong Kong Chinese Materia Medica Standards (HKCMMS) (HKCMMS, 2012), with modifications for 50 ± 5 mg tissue (exact weight recorded) in 2 mL tubes. Samples were extracted in 600 μL 30% ethanol (v/v) + 20 μL ammonium hydroxide solution (28%, v/v), with a 10 s vortex to resuspend the pellet followed by 30 min in a 65W sonication bath (at room temperature). After centrifugation (13,000 × *g* for 5 min), 500 μL of supernatant was transferred to a fresh tube and placed on ice. The pellet was re-extracted (twice) with 600 μL 30% ethanol (v/v) + 20 μL ammonium hydroxide solution (28%, v/v) as before, and the supernatants combined. An additional 500 μL of 30% ethanol (v/v) was added to the pooled supernatants (total 2 mL of extract). Insoluble material was removed from the soluble extracts by centrifugation (20,000 × *g* for 5 min) and 100 μL of supernatant was removed and fully dried in a SpeediVac (Savant). The remaining supernatant was removed and stored at −20°C, and subsequently used to prepare concentrated extracts from some samples by drying down 1 mL of soluble extract. Dried samples were resuspended in 30–50 μL 30% ethanol (v/v) and clarified by centrifugation (20,000 × *g*) prior to transferring to HPLC vials for use and/or short-term storage at −20°C. QAs were extracted from additional aliquots of tissues used for qPCR and two control samples [dried kushen root purchased from a traditional Chinese medicine store in Adelaide (September 2019) and dry seeds, purchased online from footnote 1].

Quinolizidine alkaloids were analysed using an Agilent 1200 HPLC unit, with a diode array detector (set to record at 220 nm) and a Zorbax Eclipse Plus C18 rapid resolution column (HD 2.1 × 50 mm, 1.8 μ, Agilent, CA, United States). HPLC was performed using a mobile phase composed of A (0.3% phosphoric acid and 0.912% triethylamine, pH 6.0 in milliQ water) and B (0.3% phosphoric acid and 0.912% triethylamine, pH 6.0 in 70% acetonitrile) with a gradient elution from 4% A to 10% B over 10 min, flow rate 0.4 mL/min, column temperature 35°C. The injection volume was 2 μL. Sigma standards were used to establish identity of oxymatrine (O0891) and matrine (M5319) based on their relative retention times and published UV spectra (Ling et al., 2007). A standard curve was produced from a mixture of oxymatrine and matrine, with oxymatrine concentrations of 50, 100, 200, 400, and 800 ng/μL and matrine of 2.5, 5, 10, 20, and 40 ng/μL in 30% ethanol (from low to high, respectively), reflecting the higher levels of oxymatrine to matrine expected in root tissues.

## Results

### DArT Sequencing of Nine Populations of *S. flavescens* From China and Genetic Diversity Analysis

An initial round of DArTseq using four DNA samples (one from each of Liaoning, Jilin, Shaanxi, and Guizhou provinces: regions 1, 3, 7, and 9, respectively, Figure 2A) yielded 2212 SNP-bearing tag pairs, 1392 of which were scored for all four plants. Of the genotypes scored for these 1392 SNPs, 78.7% were homozygous and 21.3% were heterozygous. Nineteen SNPs were selected for which all four plants exhibited homozygosity (two of each genotype or three of one genotype and two of the other). KASP primer sets (He et al., 2014) were designed for these SNPs and applied to the same four DNA samples. Fifteen of these markers clearly distinguished the two alternative homozygous genotypes (Supplementary Table 2). Of the 60 possible genotypes (15 markers × 4 plants), 58 were scored based on the KASP markers (exceptions: Sf17 and Sf19 for the plant from region 1). Of these, 52 (90%) were identical to the homozygous genotypes from the DArTseq analysis, five (Sf5, Sf7, Sf10, and Sf15 for the plant from region 1; Sf17 for the plant from region 3) were heterozygous and only one (Sf6 for the plant from region 9) contradicted the result from the DArTseq analysis (A:A vs. T:T). Failure of the DArTseq analysis to detect heterozygosity may be due to sequencing depth differing between alleles, with one allele failing to meet the threshold required to call a heterozygote.

DArTseq analysis performed with 89 DNA samples produced 10,599 SNPs, of which 1636 had less than 5% missing data and minor allele frequencies greater than 5%. With phylogenetic analysis of the data for the 1636 SNPs, most plants from each province grouped together and plants from neighbouring provinces clustered together (Figure 2B). There were, however, some exceptions to the tight clustering of samples from the same region. For example, the 10 plants from region 5 (Huairou County in Beijing province) were distributed across three clades: one of six plants, another of three plants, and there was a single plant that grouped with most of the accessions from region 6 (Qin County in Shanxi province). These exceptions could be due to human movement of plants or seeds and/or selection of specific characteristics. Overall, it seems that accessions from Qin County (Shanxi province, region 6) may have diverged early and that different changes occurred in the north–east (regions 1–4) and south–west (regions 7–9) of China.

**FIGURE 3:**
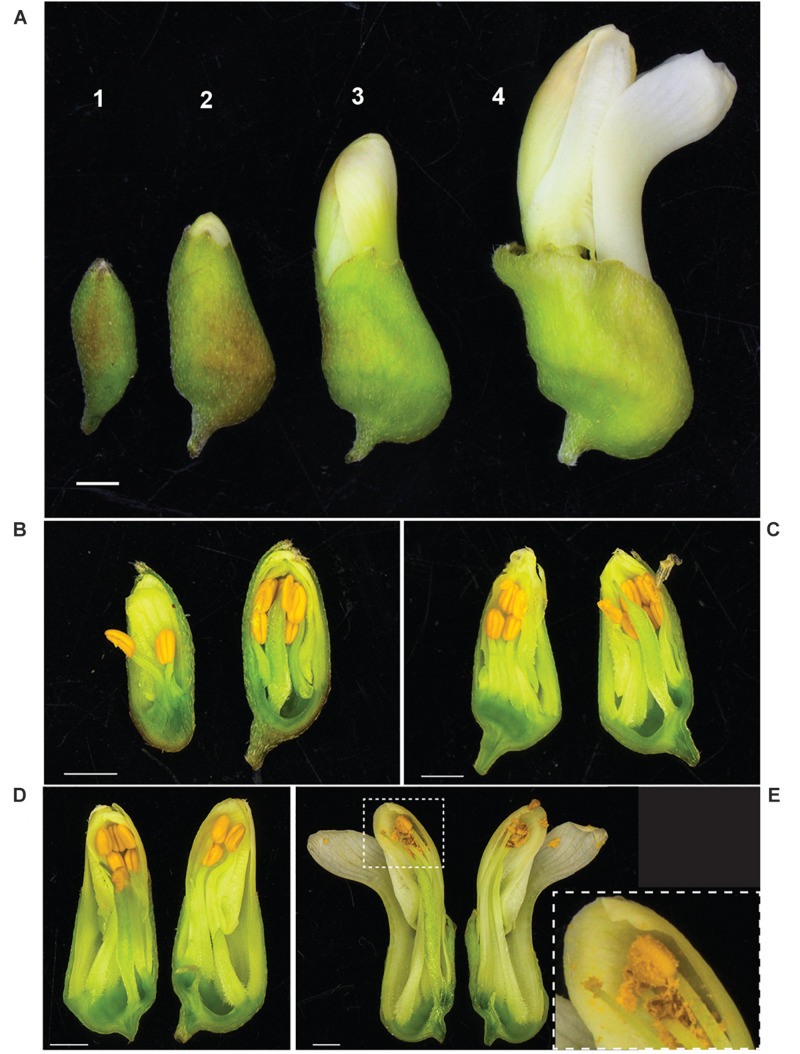
Anther dehiscence occurs before flowers fully open in *S. flavescens*. **(A)** Four discernible stages of flower development (1–4) prior to fully opened flowers. Longitudinal sections of representative buds at each stage **(B–E)** shows that by stage 4 **(E)** most of the pollen has dehisced, the keel petal has “opened,” but the other four petals are still together at the apex of the bud. Inset panel **(E)** shows a close up of the style, stigma, and one anther with released pollen. *S. flavescens* is in subfamily Papilionoideae and has a typical papilionoid-type flower with a forward facing petal, called a keel petal, and four other petals (Tucker, 2003). Scale bars, 1 mm.

**FIGURE 4:**
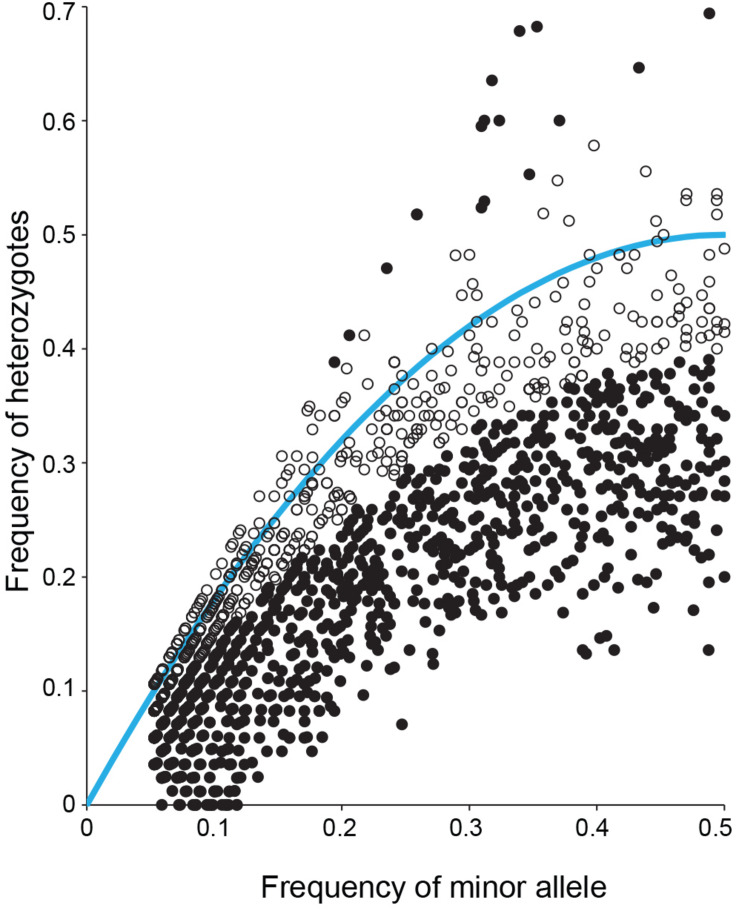
Observed frequency of heterozygotes and minor allele frequency for each of 1636 SNPs detected by DArTseq genotyping by sequencing of 85 *Sophora flavescens* plants from nine regions in China. Solid black symbols represent markers for which observed genotype frequencies deviated significantly (*p* < 0.05) from Hardy–Weinberg equilibrium. Open black symbols represent markers for which observed genotype frequencies did not deviate significantly from Hardy–Weinberg equilibrium. The blue curve represents the expected frequency of heterozygotes given the minor allele frequency.

It was observed that anthesis can occur before flowers open (Figure 3), indicating that *S. flavescens* could be capable of self-pollination. Among 1636 SNPs with less than 5% missing data and with minor allele frequencies greater than 0.05 (Supplementary Table 3), the overall frequency of heterozygosity was 0.21. Among these SNPs, 1070 (65.4%) had genotypic frequencies that deviated significantly (*p* < 0.05) from Hardy–Weinberg expectations, according to Chi-square tests conducted with one degree of freedom. For 1053 of these markers (98.4%), the observed frequency of heterozygotes was lower than the expected frequency (Figure 4).

### Obtaining Full Length Sequence for *SfCAO*

**FIGURE 5:**
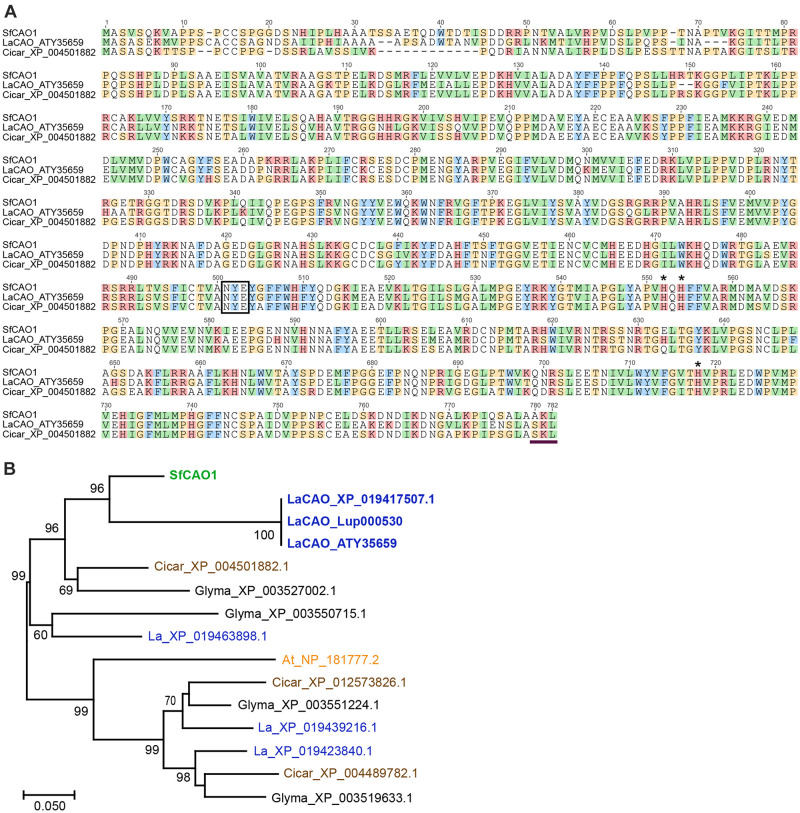
Sequence and analysis of deduced SfCAO protein sequence derived from the targeted capture and assembly of *S. flavescens* short read sequences matching *LaCAO*. **(A)** SfCAO was aligned with LaCAO (XP _019417507.1) and Cicar_XP_004501882.1 using Muscle (https://www.ebi.ac.uk/Tools/msa/muscle/) and the resulting alignment formatted in Geneious. The box indicates the conserved NYE/X motif and the three histidines (*) indicate the residues that interact with the catalytic copper ion (Yang et al., 2017). The horizontal line at the C-terminus indicates a predicted peroxisomal targeting signal peptide (Reumann et al., 2012). **(B)** Maximum likelihood tree (MEGA) of *CAO* genes from *S. flavescens* (Sf, green), *L. angustifolius* (La, blue), *C. arietinum* (Cicar, chickpea, brown), *Glycine max* (Glyma, black), and *Arabidopsis thaliana* (At, orange). Species known to produce QAs (Sf and La) are in bold. Nodes with ≥60% support (1000 bootstrap replicates) are indicated. The three sequences that are labelled LaCAO protein represent the CAO protein involved in QA biosynthesis from three different sources: ATY35659 [protein encoded by the cDNA sequence reported in Yang et al. (2017)], XP_019417507.1 (NCBI), and the protein predicted from the gene, *Lup000530* as used to predict intron positions (legumeinfo.org, with some manual reannotation, see Supplementary Figure 8). One of 10 *Arabidopsis thaliana* CAO proteins (NP_181777.2 (= AtCuAO3), encoded by *At2g42490*) was selected based on data showing that the protein groups with other legume CAOs and the gene encoding this protein is the only *Arabidopsis* CAO gene that has 11 introns as found in lupin (Tavladoraki et al., 2016). The scale represents the number of amino acid substitutions per site.

To develop gene-specific SNP markers for *LDC* and *CAO* we needed to sequence both genes, however, there was no full-length sequence for *SfCAO* in the NCBI databases. We used mirabait (Chevreux et al., 2004) at low stringency to identify short read RNA sequences matching *SfCAO* from two publicly available but not assembled datasets for *S. flavescens* (Han et al., 2015). After assembly and analysis, the best contig was used in a second round of mirabait to obtain a full length *SfCAO* cDNA sequence from dataset DRR031281 (Supplementary Figure 4A). It was 86.9% identical to the coding sequence of *LaCAO* (MF152953) and consisted of 104 bp of 5′ UTR, 2343 bp of coding sequence, and 169 bp of 3′ UTR (Supplementary Figure 4A). Alignment of the encoded protein with LaCAO and a CAO protein from *Cicer arietinum* (chickpea) shows that the protein encoded by the *SfCAO* cDNA contains the important catalytic residues and a C-terminal peroxisomal targeting signal as expected (Reumann et al., 2012; Figure 5A). Phylogenetic analysis provides strong support that *SfCAO* is the orthologue of *LaCAO* involved in QA biosynthesis (Yang et al., 2017; Figure 5B).

Full length transcripts were also obtained by mirabait for *SfLDC* and different variants (1 and 2) were obtained from the two datasets, DRR031281 and DRR031283, respectively. The variants were greater than 98% identical to the *SfLDC* coding sequence at NCBI (AB561138.1) and differ from each other only at 4 positions (data not shown).

### SNPs Identified for the *SfLDC* Gene

Ten DNA samples were selected for SNP discovery in *SfLDC*, one from each regional clade and one commercial sample (Figure 2B). For *SfLDC*, in which no introns were expected, PCR primers were designed in the 5′ and 3′UTRs of *SfLDC* to enable sequencing of the full length *SfLDC* gene (SfLDC_F_5pr and SfLDC_R1, respectively, Supplementary Table 1). The forward primer SfLDC_ F_5pr was designed based on sequence alignment between *SfLDC* AB561138.1, several short reads from *S. flavescens* dataset DRR031281 and a 5′ UTR sequence from *S. alopecuroides LDC* (KY038928.1) (Supplementary Figure 5). The reverse primer *SfLDC*_R1 was designed based on *SfLDC* AB561138.1. For eight of 10 samples, PCR products of the expected size were obtained with standard PCR cycling conditions (annealing at 65°C). For plants 4_15 and 9_8, standard cycling at a reduced annealing temperature of 55°C resulted in multiple bands (not shown) whereas touchdown PCR (Korbie and Mattick, 2008) provided a single amplification product of the expected size. There may be one or more mismatches between the primer(s) and the template DNA for these two samples.

The SNPs identified in *SfLDC* by Sanger sequencing are detailed in Supplementary Table 4, with example chromatograms in Supplementary Figure 6A. *SfLDC* genomic DNA sequences of the 10 samples are reported in Supplementary Figure 4B and aligned in Supplementary Figure 7A. A total of 14 SNPs were identified, of which six are predicted to result in amino acid changes in the encoded protein (Supplementary Figure 7B). There were several highly polymorphic SNPs where both homozygotes and the heterozygote were observed (positions 41, 381, 459, and 1341, Supplementary Figure 7A).

### PCR Identifies Two Recently Duplicated Genes, *SfCAO1* and *SfCAO2*

The second gene in the QA pathway, *CAO*, is expected to have 12 exons (11 introns), based on the 6520 bp lupin *CAO* gene sequence (*Lup000530*), with one very long intron (intron 1, 1931 bp) (Supplementary Figure 8). For this gene, PCR primers were designed to amplify a short partial 5′ sequence (5′ UTR and most of exon 1) and a 3′ partial sequence (part of exon 10 through to the 3′ UTR).

A 5′ product of the expected size (247 bp) was obtained and sequenced, revealing two polymorphic positions (Supplementary Figures 6B,C and Supplementary Table 5). Surprisingly, all samples were heterozygous at the first SNP (position 81) and 9 of 10 samples were heterozygous at the second polymorphic position (position 220). A plausible explanation for the high heterozygosity became apparent when the 3′ partial fragment was amplified, as two strong products were produced in most samples (Supplementary Figure 9A), indicating that there may be two closely related *CAO* genes, hereafter called *SfCAO1* and *SfCAO2*, that share the same primer binding sites. Most samples produced a lower band of the expected size ≈ 720 bp (compared to the lupin gene *Lup000530*, ≈750 bp) and all samples produced an upper band (≈1200 bp). Sample 4_15 did not produce the 720 bp band and sample 9_8 produced a smaller PCR product, ≈550 bp) (Supplementary Figure 9A). Sequencing revealed that the difference between the 1200 and 720 bp PCR products is due to longer introns in *SfCAO2* compared to *SfCAO1*. For example, in sample 1_12, *SfCAO2* has the same exon sequence as *SfCAO1*, however, intron 10 is 40 bp longer (at 281 bp) than for *SfCAO1* and intron 11 is 399 bp longer (at 546 bp) (Supplementary Figures 9B–D).

**FIGURE 6:**
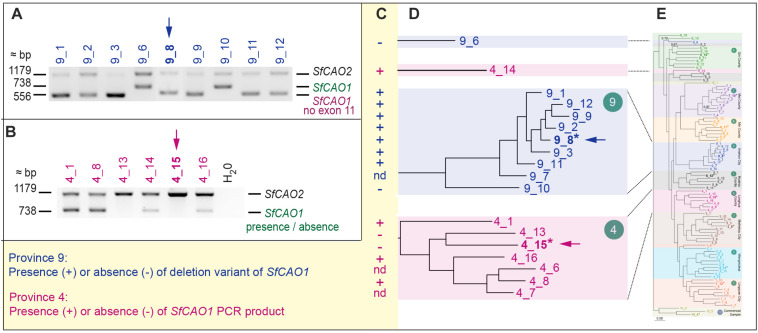
Selected samples from regions 4 and 9 have consistent but different changes in *SfCAO1*. **(A)** Amplification of 3′ *SfCAO* gene fragments from nine individuals from Anshun Province (region 9) reveals diversity in *SfCAO1* for the 181 bp deletion from intron 10 to intron 11 (bases 271–451, including exon 11). This deletion was originally identified by Sanger sequencing of sample 9_8 (*, blue arrow). **(B)** Amplification of 3′ *SfCAO* from six individuals from Hebei Province (region 4) reveals diversity for presence/absence of *SfCAO1* with primers SfCAO_5p_Fd and SfCAO_e1_R. Sample 4_15 (*, pink arrow) was the sample selected for sequencing and thus only *SfCAO2* could be sequenced from this sample. **(C)** Summary of presence or absence of: in samples from region 9, the deletion variant allele (containing a 181 bp deletion that codes for exon 11 and flanking intron sequence); or in samples from region 4, the *SfCAO1* PCR product, based on the position of samples in the relevant clades **(D)** of the phylogenetic tree produced from 2600 DArTSeq SNPs. **(E)** A scaled down copy of the entire tree from Figure 2B (to provide context).

Sequencing of the smaller than expected PCR product for *SfCAO1* in sample 9_8 (Supplementary Figure 9A) showed that the reduced size is due to a 181 bp deletion (from position 271 in intron 10 to position 451 in intron 11) that results in loss of exon 11 (Supplementary Figure 7D). Exon 11 is expected to be critical for CAO activity as it contains the codon for one of three catalytic His residues in the CAO domain (Pfam PF01179) (Supplementary Figure 8B). A PCR check showed that seven of nine other plants from region 9 also have the 181 bp deletion (Figures 6A–C). The two samples that do not have the deletion (that is they contain the full length product including exon 11 sequence) are sample 9_10, the more ancestral sample of the main group from region 9, and an “outlier” sample, sample 9_6, which grouped with 6_13 in the phylogenetic tree produced with 1636 DArTSeq SNPs (Figures 2B, 6D,E).

Two of six samples tested from region 4 did not produce a PCR product for the 3′ fragment of *SfCAO1* with the primers used (Figure 6B). These two samples, 4_15 and 4_13, are more closely related to each other than the other four samples tested from region 4 based on phylogenetic analysis (Figures 2B, 6D,E).

### SNPs Identified for *SfCAO1* and *SfCAO2* Genes

Single nucleotide polymorphism were identified in both *SfCAO1* and *SfCAO2* (Supplementary Figures 4D,E, 7D,E and Supplementary Tables 6, 7). Both genes contained a simple sequence repeat (SSR) polymorphism (CT)_n_ near the start of intron 10, for which the number of CT repeats ranged from 6 to 8 in *SfCAO1* and from 7 to 9 in *SfCAO2* (Supplementary Figure 10 and Supplementary Table 8). Samples that were heterozygous for repeat length could not be fully sequenced due to the frameshift created (Supplementary Figures 7D,E, 10). More SNPs were identified in *SfCAO1* than in *SfCAO2*, including three in the coding sequence of *SfCAO1* (Supplementary Tables 6C,D). One of these changes, [G/A]AG, at position 335 results in a non-conservative change from Glu to Lys in the encoded protein.

Single nucleotide polymorphisms were also identified between two full length cDNAs for *SfCAO*. Prior to our discovery of two putative *SfCAO* genes involved in QA metabolism, we had collected tissue from two field grown plants, 1_LC1 and 9_AC1, for expression analysis (qPCR) and quantification of secondary metabolites (see section “Tissue-Specific Expression of Two QA Pathway Genes and Accumulation of Oxymatrine”). One of these plants, 9_AC1 was grown from seeds collected in region 9, and therefore potentially contained the deletion variant of *SfCAO1* missing exon 11 (Figure 6A), so may not produce a full-length cDNA. Genomic DNA was extracted from 9_AC1 and PCR showed that this sample has the deletion variant of *SfCAO1* (Supplementary Figure 11), however, both 1_LC1 and 9_AC1, produced full length *SfCAO* cDNA sequences. In both cases, only a single PCR product of the expected full-length size was produced (≈2350 bp, Supplementary Figure 11). Sequencing confirmed a full-length *CAO* cDNA in stem tissue from sample 9_AC1 (Supplementary Figure 4F), indicating that *SfCAO2* is expressed. Absence of a smaller cDNA PCR product in 9_AC1 indicates that the deletion variant of *SfCAO1* is not expressed at detectable levels in stem tissue. A total of five SNPs were identified between 1_LC1 and 9_AC1 cDNA sequences and a further five were identified compared to the *SfCAO* sequence assembled from the RNASeq data (Supplementary Table 9).

### Gene-Specific KASP Markers for *SfLDC* and *SfCAO*

To confirm the utility of the *SfLDC* and *SfCAO* SNP data we designed gene specific KASP markers. Seven *SfLDC*-specific markers and three *SfCAO1*-specific markers were successfully designed and used to genotype 87 of the 89 samples analysed by DArTSeq (Supplementary Table 3). All of the relevant polymorphisms identified by Sanger sequencing (Supplementary Tables 4, 6) were confirmed by the KASP markers, bringing the total number of KASP markers developed to 27 (Supplementary Tables 2, 10).

### Tissue-Specific Expression of Two QA Pathway Genes and Accumulation of Oxymatrine

**FIGURE 7:**
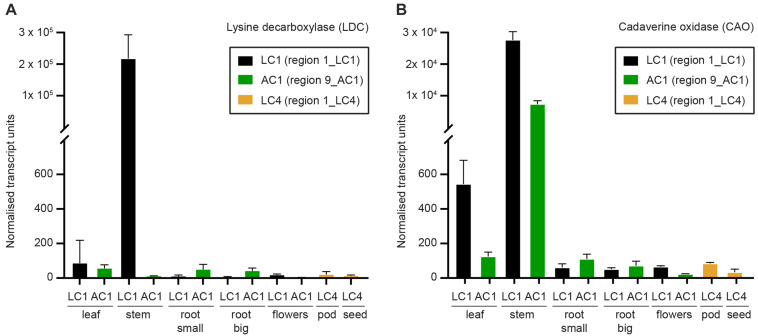
Expression of the first two genes in the quinolizidine alkaloid pathway as determined by quantitative real time PCR (qPCR) as described by Burton et al. (2011). **(A)** Lysine decarboxylase (*SfLDC*) and **(B)** cadaverine oxidase (*SfCAO*) normalised transcript levels (arbitrary units). Error indicates standard deviation of three replicate tissues samples from the same plant.

To determine where in *S. flavescens* plants *SfLDC* and *SfCAO* are expressed, a tissue series of cDNA samples was developed from tissues growing at the field trial site at Waite Campus, South Australia. Quantitative PCR analysis revealed that both *SfLDC* and *SfCAO* were highly expressed in stem tissue in plant 1_LC1, at 2.2 ± 0.8 × 10^5^ units and 2.8 ± 0.3 × 10^4^ units, respectively, whereas *SfCAO*, but not *SfLDC*, was highly expressed in stems of 9_AC1, at 7.3 ± 1.1 × 10^3^ units (Figure 7). Surprisingly, the expression level of *SfLDC* in stems of 9_AC1 plants was over 500-fold lower at 11 ± 2 units. Expression of the genes in the other tissues was generally low (<100 units), with the exception of expression of *SfCAO* in leaves of both 1_LC1 and 9_AC1 (at 544 ± 138 units and 125 ± 26 units, respectively), and root (small) 9_AC1 (110 ± 28 units).

**FIGURE 8:**
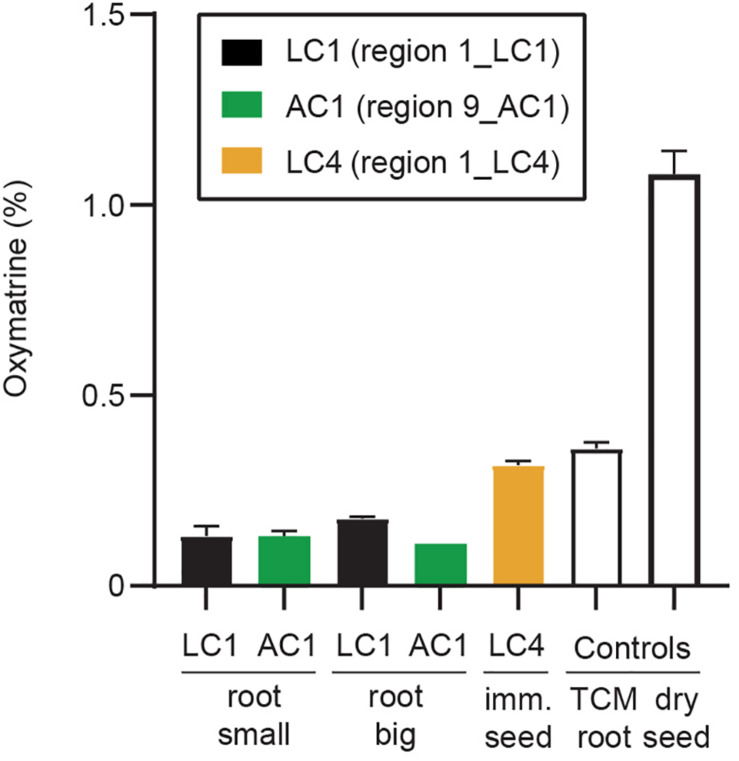
Quantification of oxymatrine. Oxymatrine (%) was determined by HPLC based on peak area compared to a standard curve produced by a commercial standard (Sigma). Oxymatrine was detected in root samples (both large and small root) (Supplementary Figure 1), immature seeds (Supplementary Figure 3), and the two control samples (kushen root from a TCM store, and dry commercial seed). Error bars indicate standard deviation of three replicate tissues samples from the same plant (except for sample AC1, small root, *n* = 2). Example chromatograms (concentrated samples) are shown in Supplementary Figure 12 and show that low levels of matrine were detected in the immature seeds (LC4), and the two control samples.

Quinolizidine alkaloid metabolites were extracted, and their levels were quantified to determine if oxymatrine and matrine were produced in young plants growing in Australia. Samples used were subsamples of the tissues used for qPCR. Oxymatrine was detected at low levels in samples from both large and small roots (ranging from 0.11 to 0.18% fresh weight) and at higher levels in immature seeds (0.32% fresh weight) (Figure 8). Matrine was only detected in one of the field samples, the immature seeds (0.007 ± 0%), at similar levels to that found in the kushen root (dried) purchased from a TCM store (0.004 ± 0%). The highest level of matrine was found in the commercial sample of dry *S. flavescens* seeds (0.26 ± 0.02%) (Supplementary Figure 12). No oxymatrine or matrine were detected in flower, leaf, stem, or seedpod tissues (data not shown).

## Discussion

Our research provides much needed information on the molecular diversity of *S. flavescens* plants from a large part of its observed range within China. A combination of techniques has provided resources for the future, with over 10,000 SNPs identified and 27 of these validated through the development of high throughput KASP markers. These and the other results are discussed below in the context of the existing literature and future plans to achieve more control of secondary metabolite production in *S. flavescens* through the selection of diverse and genetically defined germplasm.

New information was provided for *SfCAO*, the second gene in the QA pathway, with its cDNA sequence obtained using targeted capture and assembly from publicly available RNASeq data using the lupin *LaCAO* sequence (Han et al., 2015; Figure 5). *LaCAO* was recently identified as one of several genes involved in the domestication of sweet lupin varieties, with low *LaCAO* gene expression contributing to the sweet/low alkaloid phenotype (Plewiński et al., 2019). We have identified a key difference between lupin and *S. flavescens* with the duplication of the *CAO* gene. We found no evidence for another lupin gene (data not shown) that groups with the *LaCAO* gene QA pathway (Figure 5B) in the available lupin genomes [at NCBI^5^, Legume Information System^6^ and the recently published lupin genome assembly (Wang et al., 2021)].

Gene duplication is a key process in the evolution and diversification of secondary metabolite production by providing opportunities for either sub-functionalization (greater substrate specificity or more restricted range of substrates) and/or neo-functionalisation (new activities) (Michael, 2017). Most of the plants from region 9 (Anshun City, Guizhou Province) have a 181 bp deletion in *SfCAO1* that results in the loss of exon 11 sequence (and part of the flanking intron sequences) (Figure 6A) which encodes one of three catalytic His residues (Figure 3 and Supplementary Figure 8B). This deletion would almost certainly abolish CAO activity, possibly making this change an evolutionary “dead-end.” We did not detect expression of the deletion variant of *SfCAO1* but we found that a full-length cDNA, presumably from *SfCAO2*, was expressed in stem tissue of sample 9_AC1 (Supplementary Figure 11). If the expression of the deletion variant of *SfCAO1* is expressed in any accessions (e.g., in other tissues or with different primers), comparison of the metabolites produced by plants from region 9, with and without the deletion (e.g., 9_8 and 9_10) would be warranted. The similar levels of oxymatrine in the roots of 1_LC1 and 9_AC1, demonstrate that the QA pathway is not compromised by the 181 bp deletion in *SfCAO1*, indicating that *SfCAO2* is functional in these plants.

It was not possible to amplify the *SfCAO2* gene from plant 1_LC1 (Supplementary Figure 11) or the *SfCAO1* gene from two plants from region 4 (Figure 6B). This could be due to divergent sequence at one or both primer binding sites, rather than loss of one of the duplicated genes. Although touchdown PCR worked to amplify *SfLDC* from 4_15, it was not successful at amplifying the 3′ fragment of *SfCAO1* from 4_15 (Supplementary Figure 9A) or *SfCAO2* from 1_LC1 (Supplementary Figure 11A). New *SfCAO* primers can be designed and tested and if that is not successful, other techniques would have to be explored to determine the presence or absence of *SfCAO1* such as Southern blot analysis (Southern, 2000), genome sequencing (Wang et al., 2021), and/or isoform sequencing (Hardwick et al., 2019).

Full genome sequencing of a variety of accessions would have obvious advantages including obtaining full length genomics sequences for *SfCAO1* and *SfCAO2* (expected size ≈ 6500 bp). The coding sequences obtained in this work, part of exons 1 and 10 and all of exons 11 and 12, were identical between the two genes in most varieties, noting that in some samples not all of exon 11 could be sequenced due to frameshifts created by SSRs in the introns (Supplementary Figures 4D,E, 7D,E, 10). Full length genomic sequence would also allow us to determine: (1) whether the apparent SNPs in exon 1 (Supplementary Figures 6B,C) are actually genic differences in *SfCAO1* and *SfCAO2* and (2) whether there are sequence differences between the two full length coding sequences that could lead to functional or regulatory differences between the encoded proteins. The second question in particular could have important implications for QA metabolite production.

The utility of the DArTSeq GBS approach was clearly demonstrated with the finding of over SNPs (1636 SNPs with <5% missing data and minor allele frequencies >5%) providing robust data for phylogenetic analysis (Figure 2B). By selecting one sample from the main cluster of each region for more detailed sequencing of *SfLDC* and *SfCAO* genic sequences we were able to identify 14 SNPs in *SfLDC* and even more (16) in *SfCAO1* by sequencing just 4 of the 11 exons. Fewer SNPs (8) were identified in the corresponding region of *SfCAO2* despite its longer introns.

High heterozygosity is one of many recognised challenges facing plant breeding of medicinal plants (Wang et al., 2020), and one expected for *S. flavescens* based on anecdotal evidence that it was an outbreeding species. However, in attempting to make crosses, we noted that anthesis can occur before flowers open (Figure 3), raising the possibility that the species might be self-pollinated. Analysis of SNP genotypic data for 85 plants from nine regions in China revealed many significant deviations from Hardy–Weinberg equilibrium and confirmed that heterozygosity was often lower than expected based on allele frequencies. However, the overall frequency of heterozygosity (0.21) was much higher than would be expected with complete or nearly complete self-pollination and the deviations from Hardy–Weinberg equilibrium are likely to be mainly due to population structure, with allele frequencies varying among regions due to selection or genetic drift, in combination with some degree of reproductive isolation. Nevertheless, it would be interesting to investigate whether self-pollination occurs and whether it leads to inbreeding depression. To determine if self-pollination occurs would require knowledge of whether the stigma is receptive before and/or after the flowers fully open. This information could help determine which breeding methods could be implemented for genetic improvement of *S. flavescens*.

A reasonable number of the SNPs detected in coding sequences result in amino acid changes [6 of 13 from *SfLDC* gDNA (Supplementary Table 4) and 3 of 5 from *SfCAO1* gDNA (Supplementary Table 5) and 6 of 15 from *SfCAO* cDNA sequences (Supplementary Table 9)]. Not all the amino acid changes are conservative and therefore any future work should consider molecular modelling to determine the impact of these changes on enzyme function.

To the best of our knowledge, this is the first published report of qPCR based expression data for QA pathway genes in *S. flavescens*. Semi-quantitative RT-PCR was performed by Han et al. (2015) for *SfLDC* comparing three tissues (leaf, stem, and root) and they observed about twofold higher *SfLDC* levels in leaves compared to stems, which was consistent with their RNAseq data. In contrast, we observed higher levels in stems than in leaves. For a plant from region 1 (1_LC1) we observed over 2 × 10^5^ normalised transcript levels for both *SfLDC* and *SfCAO* in stem tissue, whereas for the sample from region 9 (9_AC1) there were high levels of *SfCAO* expression but almost non-detectable levels of *SfLDC* expression. With the exception of the low *SfLDC* transcript expression in the stems of 9_AC1 all of the other data is as expected and consistent with the expectation that *LDC* and *CAO* are expressed in green tissues, predominantly leaves, and stems with generally lower expression in seedpods, with very low levels in roots, flowers, and seeds (Han et al., 2015; Yang et al., 2017; Otterbach et al., 2019).

The tissue series we developed will provide a quick way to evaluate other candidate genes involved in control of QA metabolism. Co-expression studies with *LDC/CAO* in lupin (and *LDC* in *S. flavescens*) have identified a growing number of candidate genes involved in QA accumulation in as yet unknown ways. The candidate genes include transcription factors, transporters, and a range of potential genes likely involved in metabolite modification (Han et al., 2015; Yang et al., 2017; Kroc et al., 2019; Plewiński et al., 2019; Kamphuis et al., 2020). For candidate genes identified in lupin, we have demonstrated that a mirabait transcript capture (Chevreux et al., 2004) and assembly strategy is an efficient way to use existing resources to obtain transcripts for candidate genes.

For kushen products to be acceptable for use as a TCM, it is required by the Chinese Pharmacopoeia Commission to contain a 1.2% total level of matrine and oxymatrine [reviewed in He et al. (2015)]. The kushen root (TCM-root) we purchased from a reputable TCM store in Australia had barely detectable levels of matrine (0.004%), and the measured oxymatrine level of 0.36 ± 01% is about threefold lower than the expected minimum level, highlighting the need for a better understanding of the genetic and environmental factors controlling QA metabolism. The relatively young field grown plants (≈1.5 years old) had even lower levels of oxymatrine (and no matrine) (Figure 8). It is widely believed that young *S. flavescens* plants do not produce as much matrine and oxymatrine as older plants, although we could not find any English scientific literature that documents the actual timing of increased production. A recent paper on the flavonoids produced by *S. flavescens* showed statistically significant differences from year 1 to year 6 (Chen et al., 2020).

We are confident that if matrine was present in the Australian-grown root samples, it would have been detected as we could detect it in the commercial dried seeds and the immature seeds from plant 1_LC4. We had no issues with the stability or oxidation of the matrine control (Sigma) even after multiple freeze thaw cycles (−20°C) and we had good recovery of matrine in a mock extraction control with the QA extraction and storage protocols (data not shown). Our preliminary results showed little difference in oxymatrine concentration between big tuberous roots and small roots (Figure 8). If this can be confirmed with more samples, it will make future phenotyping for QAs more efficient and less disruptive for the plant as it will be possible to harvest smaller (lateral) roots rather than the initial strategy used here of sawing the large root mass in half (Supplementary Figure 1).

We are now well positioned to perform more detailed phenotyping of field grown plants in future research. While evaluation of plant growth, metabolite profiling and transcript analysis can be time consuming and expensive, they have the potential to provide vital information to underpin successful production of pharmacologically active compounds, whether from roots or from seeds.

With the development of mapping populations for *S. flavescens*, it will be possible to investigate the genetic control of important traits. We are now in a strong position to make informed decisions about the most informative plants to select as parental combinations to maximise diversity. GBS could be applied to mapping populations and SNPs can readily be converted to KASP markers for high-throughput application in selective breeding. It would be highly desirable to have a complete genome sequence, as well as isoform sequencing. All of this is achievable with current technology, indicating that rapid progress is possible despite the constraints of working with a species that takes years to reach harvest potential.

## Data Availability Statement

The datasets presented in this study can be found in online repositories. The names of the repository/repositories and accession number(s) can be found in the article/Supplementary Material.

## Author Contributions

KC and JLi planned and executed the collection of seeds in China. KC established and maintained the field trial site. KC and DM developed the strategy for DArTSeq and KASP marker design and supervised its implementation. SNG prepared the DNA samples for DArTSeq, designed and assayed the KASP markers (designed from DArTSeq SNPs), and conducted the SNP based phylogenetic analysis. KC, RB, TB-M, and CS designed the strategy for analysis of QA genes, qPCR and QA metabolite analysis. KL performed the microscopy on the developing flower buds. CS obtained the *SfCAO* cDNA sequences (*in silico* and experimentally) and, performed and analysed the Sanger sequencing of *SfLDC* and *SfCAO* gene fragments. CS performed the purification and quantification of oxymatrine and matrine with significant protocol development assistance from JLa. CS, DM, and KC wrote the manuscript, with input from all authors. All authors have read and approved the final manuscript.

## Conflict of Interest

The authors declare that the research was conducted in the absence of any commercial or financial relationships that could be construed as a potential conflict of interest.

## Publisher’s Note

All claims expressed in this article are solely those of the authors and do not necessarily represent those of their affiliated organizations, or those of the publisher, the editors and the reviewers. Any product that may be evaluated in this article, or claim that may be made by its manufacturer, is not guaranteed or endorsed by the publisher.

## Footnotes

1 https://en.wikipedia.org/wiki/Shennong_Ben_Cao_Jing (accessed March 12, 2021).

2
Alibaba.com

3
www.diversityarrays.com/technology-and-resources/dartseq/

4
AGRF.com.au

5
https://www.ncbi.nlm.nih.gov/

6
legumeinfo.org

## Supplementary Material

Supplementary Figure 1 Plants prior to harvest and photographs of the harvested root pieces.

Supplementary Figure 2 Photos of a test plant used to develop a consistent harvesting strategy.

Supplementary Figure 3 Developing seedpods on plant 1_LC4 (grown from seeds collected in Lingyuan City, Liaoning Province).

Supplementary Figure 4 *Sophora flavescens* DNA sequences obtained during this study.

Supplementary Figure 5 Alignment (muscle) for *SfLDC* 5′ UTR primer design.

Supplementary Figure 6 Example chromatograms showing SNPs identified in *SfLDC* **(A)**, exon 1 of *SfCAO* **(B,C)**, and intron 11 of *SfCAO1* **(D)**.

Supplementary Figure 7 Alignments of sequences generated from Sanger sequencing of PCR products of genomic DNA (gDNA). *SfLDC* **(A)**, and its encoded protein **(B)**, and *SfCAO* **(B–E)**.

Supplementary Figure 8 **(A)** Genomic DNA sequence (6520 bp) of *LaCAO* with translation (above the DNA sequence). **(B)** Alignment of predicted protein sequences of *S. flavescens*, SfCAO and *L. angustifolius*, LaCAO.

Supplementary Figure 9 PCR suggests two *SfCAO* genes related to *LaCAO* cDNA (MF152953.1) involved in QA metabolism. *SfCAO1* and *SfCAO2* differ mostly in the length of their introns.

Supplementary Figure 10 Chromatograms showing different length SSR alleles for *SfCAO1* and *SfCAO2*.

Supplementary Figure 11 PCR comparison of *CAO* products from genomic DNA and cDNA.

Supplementary Figure 12 Selected HPLC chromatograms showing the elution of oxymatrine and matrine in a variety of tissues.

Supplementary Table 1 Primer names, sequences, T_m_, and their use.

Supplementary Table 2 Comparison of DArTSeq and SNP genotype results for one *S. flavescens* plant from each of four regions of China.

Supplementary Table 3 Genotypes from DArTseq analysis of 85 plants from nine regions in China, for each of 1,636 single nucleotide polymorphisms (SNPs) with no more than 5% missing data and for which the frequency of the minor allele was at least 5%.

Supplementary Table 4 Genotype information for single nucleotide polymorphisms (SNPs) identified in the *SfLDC* gene of 10 *S. flavescens* samples.

Supplementary Table 5 Genotype information for SNPs identified in *SfCAO* 5′ gene fragment amplified from 10 *S. flavescens* samples.

Supplementary Table 6 Genotype information for SNPs identified in *SfCAO1* 3′ gene fragment amplified from 10 *S. flavescens* samples.

Supplementary Table 7 Genotype information for SNPs identified in *SfCAO2* 3′ gene fragments amplified from 10 *S. flavescens* samples.

Supplementary Table 8 Lengths of the simple sequence repeat (SSR) polymorphisms in intron 10 are different in *SfCAO1* and *SfCAO2*.

Supplementary Table 9 Genotype information for SNPs identified in *SfCAO* cDNA fragments amplified from the two *S. flavescens* plants used for qPCR (Figure 7).

Supplementary Table 10 Gene-specific KASP markers for *SfLDC* and *SfCAO1*.

## References

[B1] Bunsupa S. Hanada K. Maruyama A. Aoyagi K. Komatsu K. Ueno H. (2016). Molecular evolution and functional characterization of a bifunctional decarboxylase involved in *Lycopodium alkaloid* biosynthesis. *Plant Physiol.* 171 2432–2444. 10.1104/pp.16.00639 27303024 PMC4972286

[B2] Bunsupa S. Katayama K. Ikeura E. Oikawa A. Toyooka K. Saito K. (2012). Lysine decarboxylase catalyzes the first step of quinolizidine alkaloid biosynthesis and coevolved with alkaloid production in leguminosae. *Plant Cell* 24 1202–1216. 10.1105/tpc.112.095885 22415272 PMC3336119

[B3] Burton R. A. Collins H. M. Kibble N. A. J. Smith J. A. Shirley N. J. Jobling S. A. (2011). Over-expression of specific *HvCslF* cellulose synthase-like genes in transgenic barley increases the levels of cell wall (1,3;1,4)-β-D-glucans and alters their fine structure. *Plant Biotechnol. J.* 9 117–135. 10.1111/j.1467-7652.2010.00532.x 20497371

[B4] Carvajal-Larenas F. E. Linnemann A. R. Nout M. J. R. Koziol M. van Boekel M. (2016). *Lupinus mutabilis*: composition, uses, toxicology, and debittering. *Crit. Rev. Food Sci. Nutrit.* 56 1454–1487. 10.1080/10408398.2013.772089 26054557

[B5] Chen L. Huang X. B. Wang H. Shao J. Luo Y. Zhao K. R. (2020). Integrated metabolomics and network pharmacology strategy for ascertaining the quality marker of flavonoids for *Sophora flavescens*. *J. Pharm. Biomed. Analysis* 186:113297. 10.1016/j.jpba.2020.113297 32325403

[B6] Chen X. Yi C. Q. Yang X. Q. Wang X. R. (2004). Liquid chromatography of active principles in *Sophora flavescens* root. *J. Chromatogr. B Analyt. Technol. Biomed. Life Sci.* 812 149–163. 10.1016/s1570-0232(04)00679-8 15556494

[B7] Chevreux B. Pfisterer T. Drescher B. Driesel A. J. Müller W. E. G. Wetter T. (2004). Using the miraEST assembler for reliable and automated mRNA transcript assembly and SNP detection in sequenced ESTs. *Genome Res.* 14 1147–1159. 10.1101/gr.1917404 15140833 PMC419793

[B8] Cui J. Qu Z. P. Harata-Lee Y. Shen H. Y. Aung T. N. Wang W. (2020). The effect of compound kushen injection on cancer cells: integrated identification of candidate molecular mechanisms. *PLos One* 15:e0236395. 10.1371/journal.pone.0236395 32730293 PMC7392229

[B9] Doyle J. (1987). A rapid procedure for DNA purification from small quantities of fresh leaf tissue. *Phytochem. Bull.* 19 11–15.

[B10] Edgar R. C. (2004). MUSCLE: multiple sequence alignment with high accuracy and high throughput. *Nucleic Acids Res.* 32 1792–1797. 10.1093/nar/gkh340 15034147 PMC390337

[B11] Han R. C. Takahashi H. Nakamura M. Bunsupa S. Yoshimoto N. Yamamoto H. (2015). Transcriptome analysis of nine tissues to discover genes involved in the biosynthesis of active ingredients in *Sophora flavescens*. *Biol. Pharm. Bull.* 38 876–883. 10.1248/bpb.b14-00834 26027827

[B12] Hardwick S. A. Joglekar A. Flicek P. Frankish A. Tilgne H. U. (2019). Getting the entire message: progress in isoform sequencing. *Front. Genet.* 10:709. 10.3389/fgene.2019.00709 31475029 PMC6706457

[B13] He C. Holme J. Anthony J. (2014). SNP genotyping: the KASP assay. *Methods Mol. Biol.* 1145 75–86. 10.1007/978-1-4939-0446-4_7 24816661

[B14] He X. R. Fang J. C. Huang L. H. Wang J. H. Huang X. Q. (2015). *Sophora flavescens* ait.: traditional usage, phytochemistry and pharmacology of an important traditional Chinese medicine. *J. Ethnopharmacol.* 172 10–29. 10.1016/j.jep.2015.06.010 26087234

[B15] HKCMMS. (2012). *Sophora Flavecentis Radix. Hong Kong Chinese Materia Medica Standards.* Available online at: https://www.cmro.gov.hk/hkcmms/vol4/pdf_e/Sophorae_Flavescentis_Radix_v4_e.pdf (accessed November 23, 2019)

[B16] Huelsenbeck J. P. Ronquist F. (2001). MRBAYES: bayesian inference of phylogenetic trees. *Bioinformatics* 17 754–755. 10.1093/bioinformatics/17.8.754 11524383

[B17] Kamphuis L. G. Foley R. C. Frick K. M. Garg G. Singh K. B. (2020). “Transcriptome resources paving the way for lupin crop improvement,” in *The Lupin Genome. Compendium of Plant Genomes*, eds Singh K. Kamphuis L. Nelson M. (Cham: Springer), 53–71. 10.1007/978-3-030-21270-4_5

[B18] Korbie D. J. Mattick J. S. (2008). Touchdown PCR for increased specificity and sensitivity in PCR amplification. *Nat. Protocols* 3 1452–1456. 10.1038/nprot.2008.133 18772872

[B19] Kroc M. Koczyk G. Kamel K. A. Czepiel K. Fedorowicz-Strońska O. Krajewski P. (2019). Transcriptome-derived investigation of biosynthesis of quinolizidine alkaloids in narrow-leafed lupin (*Lupinus angustifolius* L.) highlights candidate genes linked to iucundus locus. *Sci. Rep.* 9:2231. 10.1038/s41598-018-37701-5 PMC6381137 30783128

[B20] Krueger F. (2015). *A Wrapper Tool Around Cutadapt and FastQC to Consistently Apply Quality and Adapter Trimming to FastQ Files.* Available online at: http://www.bioinformatics.babraham.ac.uk/projects/trim_galore/ (accessed November 30, 2020).

[B21] Kumar S. Stecher G. Li M. Knyaz C. Tamura K. (2018). MEGA X: molecular evolutionary genetics analysis across computing platforms. *Mol. Biol. Evolu.* 35 1547–1549. 10.1093/molbev/msy096 29722887 PMC5967553

[B22] Lee M. J. Pate J. S. Harris D. J. Atkins C. A. (2007). Synthesis, transport and accumulation of quinolizidine alkaloids in *Lupinus albus* L. and *L. angustifolius* L. *J. Exp. Bot.* 58 935–946. 10.1093/jxb/erl254 17189595

[B23] Lin T. C. Sung J. M. Yeh M. S. (2014). Karyological, morphological and phytochemical characteristics of medicinal plants *Sophora flavescens* Aiton grown from seeds collected at different localities. *Bot. Stud.* 55:5. 10.1186/1999-3110-55-5 PMC5432817 28510911

[B24] Ling J. Y. Zhang G. Y. Cui Z. J. Zhang C. K. (2007). Supercritical fluid extraction of quinolizidine alkaloids from *Sophora flavescens* Ait. and purification by high-speed counter-current chromatography. *J. Chromatogr.* 1145 123–127. 10.1016/j.chroma.2007.01.080 17289059

[B25] Marçais G. Yorke J. A. Zimin A. (2015). QuorUM: an error corrector for Illumina reads. *PLos One* 10:e0130821. 10.1371/journal.pone.0130821 26083032 PMC4471408

[B26] Michael A. J. (2017). Evolution of biosynthetic diversity. *Biochem. J.* 474 2277–2299. 10.1042/bcj20160823 28655863

[B27] Ohmiya S. Saito K. Murakoshi I. (1995). Lupine alkaloids. *Alkaloids Chem. Pharmacol.* 47 1–114. 10.1016/s0099-9598(08)60153-4

[B28] Otterbach S. L. Yang T. Kato L. Janfelt C. Geu-Flores F. (2019). Quinolizidine alkaloids are transported to seeds of bitter narrow-leafed lupin. *J. Exp. Bot.* 70 5799–5808. 10.1093/jxb/erz334 31328235 PMC6812715

[B29] Plewiński P. Książkiewicz M. Rychel-Bielska S. Rudy E. Wolko B. (2019). Candidate domestication-related genes revealed by expression quantitative trait loci mapping of narrow-leafed lupin (*Lupinus angustifolius* L.). *Int. J. Mol. Sci.* 20:5670. 10.3390/ijms20225670 31726789 PMC6888189

[B30] Rambaut A. (2018). *FigTree v1.4.2, A Graphical Viewer of Phylogenetic Trees.* Available online at: http://tree.bio.ed.ac.uk/software/figtree/ (accessed November 30, 2020).

[B31] Reumann S. Buchwald D. Lingner T. (2012). PredPlantPTS1: a web server for the prediction of plant peroxisomal proteins. *Front. Plant Sci.* 3:194. 10.3389/fpls.2012.00194 22969783 PMC3427985

[B32] Sato H. Saito K. Yamazaki M. (2019). Acceleration of mechanistic investigation of plant secondary metabolism based on computational chemistry. *Front. Plant Sci.* 10:802. 10.3389/fpls.2019.00802 31293608 PMC6606707

[B33] Sato H. Uchiyama M. Saito K. Yamazaki M. (2018). The energetic viability of Δ^1^-piperideine dimerization in lysine-derived alkaloid biosynthesis. *Metabolites* 8:48. 10.3390/metabo8030048 30200334 PMC6161264

[B34] Shimizu Y. Rai A. Okawa Y. Tomatsu H. Sato M. Kera K. (2019). Metabolic diversification of nitrogen-containing metabolites by the expression of a heterologous lysine decarboxylase gene in Arabidopsis. *Plant J.* 100 505–521. 10.1111/tpj.14454 31364191 PMC6899585

[B35] Southern E. M. (2000). Blotting at 25. *Trends Biochem. Sci.* 25 585–588. 10.1016/s0968-0004(00)01702-3 11116181

[B36] Sun M. Y. Cao H. Y. Sun L. Dong S. Bian Y. Q. Han J. (2012). Antitumor activities of Kushen: literature review. *Evid. Based Complementary Alternat. Med.* 2012:373219. 10.1155/2012/373219 PMC3434675 22969826

[B37] Tavladoraki P. Cona A. Angelini R. (2016). Copper-containing amine oxidases and FAD-dependent polyamine oxidases are key players in plant tissue differentiation and organ development. *Front. Plant Sci.* 7:824. 10.3389/fpls.2016.00824 27446096 PMC4923165

[B38] Tucker S. C. (2003). Floral development in legumes. *Plant Physiol.* 131 911–926. 10.1104/pp.102.017459 12644644 PMC1540291

[B39] Wang P. H. Zhou G. F. Jian J. B. Yang H. Renshaw D. Aubert M. K. (2021). Whole-genome assembly and resequencing reveal genomic imprint and key genes of rapid domestication in narrow-leafed lupin. *Plant J.* 105 1192–1210. 10.1111/tpj.15100 33249667

[B40] Wang W. You R. L. Qin W. J. Hai L. N. Fang M. J. Huang G. H. (2015). Anti-tumor activities of active ingredients in compound kushen injection. *Acta Pharmacol. Sin.* 36 676–679. 10.1038/aps.2015.24 25982630 PMC4594177

[B41] Wang W. L. Xu J. F. Fang H. Y. Li Z. J. Li M. H. (2020). Advances and challenges in medicinal plant breeding. *Plant Sci.* 298:110573. 10.1016/j.plantsci.2020.110573 32771174

[B42] Wang Y. Zhou T. T. Li D. H. Zhang X. H. Yu W. W. Cai J. F. (2019). The genetic diversity and population structure of *Sophora alopecuroides* (Faboideae) as determined by microsatellite markers developed from transcriptome. *PLos One* 14:e0226100. 10.1371/journal.pone.0226100 31805153 PMC6894834

[B43] Weng Z. B. Guo S. Qian D. W. Zhu Z. H. Zhang S. Li A. (2017). *Sophora flavescens* seed as a promising high potential by-product: Phytochemical characterization and bioactivity evaluation. *Industrial Crops Produ.* 109 19–26. 10.1016/j.indcrop.2017.08.005

[B44] Wink M. Hartmann T. (1982). Localization of the enzymes of quinolizidine alkaloid biosynthesis in leaf chloroplasts of *Lupinus polyphyllus*. *Plant Physiol.* 70 74–77. 10.1104/pp.70.1.74 16662483 PMC1067088

[B45] Yang T. Nagy I. Mancinotti D. Otterbach S. L. Andersen T. B. Motawia M. S. (2017). Transcript profiling of a bitter variety of narrow-leafed lupin to discover alkaloid biosynthetic genes. *J. Exp. Bot.* 68 5527–5537. 10.1093/jxb/erx362 29155974 PMC5853437

[B46] Zhang H. Chen L. L. Sun X. P. Yang Q. J. Wan L. L. Guo C. (2020). Matrine: a promising natural product with various pharmacological activities. *Front. Pharmacol.* 11:588. 10.3389/fphar.2020.00588 32477114 PMC7232545

