# Supplementary material for: Analysis of Genetic Diversity in the Traditional Chinese Medicine Plant ‘Kushen’ (Sophora flavescens Ait.)
Source: Front Plant Sci. 2021 Aug 3;12:704201. doi: 10.3389/fpls.2021.704201 (PMC8369264; doi:10.3389/fpls.2021.704201)
Supplement: Supplementary Figure 1 — Plants prior to harvest and photographs of the harvested root pieces. [file Data_Sheet_1.zip › Supplementary Figure S4.DOCX]

**Figure S4 |** *S. flavescens* DNA sequences obtained during this study*.*

(**A**) *S. flavescens* sequences assembled from short read RNA-seq data after two rounds of mirabait (*SfCAO*, *SfLDC* and 5 control genes). Two RNAseq datasets were used from *S. flavescens* project (PRJDB3906, DRR031281 and DRR031283, referred to as 81 and 83, respectively in the sequencing header line) (Han et al., 2015). For *SfLDC* two variants were obtained variant 1 (from dataset DRR031281) and variant 2 (from dataset DRR031283). There are 5 SNP between the two sequences and three of them correspond to the highly polymorphic SNPs observed from sequencing the 10 *SfLDC* genes (positions 41, 381 and 459, **Figure S7**). Some of the cDNA sequences for the control genes for qPCR are partial sequences that include the 3' untranslated region. Selected partial sequences start with N or NN to give the correct reading frame for translation of uppercase text. Includes a total of 8 sequences.

**(B)** *SfLDC* full length genomic DNA sequence derived from Sanger sequenced PCR products amplified using SfLDC_F_5pr and SfLDC_R1, 10 sequences, one from each region 1 to 10).

**(C)** *SfCAO* 5' partial genomic DNA sequence derived from Sanger sequenced PCR products amplified using SfCAO_5p_Fd and SfCAO_e1_R, 10 sequences, one from each region 1 to 10).

**(D)** *SfCAO1* 3' partial genomic DNA derived from (Sanger sequenced PCR products amplified using SfCAO_e10_F and SfCAO_R1, 9 sequences, one from each region 1 to 10, except region 4 where no product was amplified from sample 4_15).

**(E)** *SfCAO2* 3' partial genomic DNA derived from Sanger sequenced PCR products amplified using SfCAO_e10_F and SfCAO_R1, 10 sequences, one from each region 1 to 10).

**(F)** *SfCAO* full length cDNA sequences derived from Sanger sequenced PCR products amplified using SfCAO_5p_Fd and SfCAO_R1, 2 sequences from samples collected from the field trial site, 1_LC1 and 9_AC1 respectively).

Upper case text is coding sequence, lowercase text is non-coding sequence (eg untranslated regions (UTRs) or introns. Unterlined text in introns indicates a region that is polymorphic in this individual for an SSR or polyN tract, creating a frameshift. Primer sequences are listed in **Table S1**.

Sequences from the “wet-bench experiments” (Fig. S4B Fig. S4F) were submitted to GenBank, NCBI accession numbers, MW960974-MW961014 (MW960974- MW960975 (Fig. S4F), MW960976- MW960985 (Fig. S4F), MW960986- MW960994 (Fig. S4F), MW960995- MW961004 (Fig. S4F), MW961005- MW961014 (Fig. S4F).

**(A)**

>SfLDC_cDNA_variant_1_mirabait2_81_c1rc

cccagttgaaatcacagtccttcacccaaaattaataaaa

ATGCCTACACTAGTAACTGA

GGCATTCCATGCCAAGGGTGCAGGACCTTTGAGCCTGAAGCCACTATTTAGTGCTTCAGG

GGTTAAGGGCAAAAGAGTCACTGCATTATCTGCAAAAGAAGAAGGTGGCATCTCTGGTTT

CATCCAATCAATCATTCACGACAAACCAGAGATGGATTCACCGTTTTTGGTGCTTGATCT

TGGGGTCGTCATGGACCTCATGGACAATTGGACCAACAACCTTCCCACAGTTCAACCTTT

CTATGCAGTTAAGTGCAACCCTAACCCATGCTTGCTGGGAGCACTGGCAGCACTCGGTTC

CAGCTTCGACTGCGCCAGTCGAGCTGAGATCGAATCCGTTTTGTCACTGGGAGTCTCACC

GGACAAAATCATCTACGCCAATCCATGCAAATCAGAGTCTCACATCAAATACGCTGCCAG

TGTGGGTGTCAACGTTACAACGTTTGACTCCAAAGAAGAGATCGACAAGATTCGAAAATG

GCACCCGAAATGTGAGCTTCTCATCCGCATCAAACCCCCAGGAGACAGCGGAGCACGAAA

TGCGTTGGGCCTCAAATACGGTGCGCTTCCTGAAGAAGTCATGCCTCTCCTCCAAGCTGC

TCAAAACGCGGGGTTGAAGGTCACCGGCGTGTCGTTTCATATCGGAAGTGGAGGAGCTGA

TTCTCAAACCTATCACGGAGCTATCGCTGCTGCTAAGAGAGTTTTCGACATGGCTTCTTC

TGAGCTAAACATGCCTAGAATGAAAGTACTGGACATTGGTGGCGGTTTCACATGTGGGAA

GCAGTTTGAGGCTGCTGCATTGCACGTGAACGAGGCTCTTCAAGTTCACTTCGGAGATGA

AGAGGGTGTCGTGGTTATTGGAGAACCGGGTCGTTATTTTGCTGAGTCAGCTTTTACGTT

GGCCAGTAAAGTTATTGGGAAGCGTGTAAGAGGCGAGGTGAGGGAGTATTGGATCGACGA

TGGGATCTACGGTTCCCTGAATTGCATAATGTTTGATTTCGCGACAGTCACGTGCTCACC

ACTCGCGTGCAGCTCAAAGCCTGAGAATCCCAGATGCAGAGACTCGAAAACGTACCCTTC

AACTGTGTTTGGTCCCACTTGCGATTCATTAGATACTATTTTCAGAGATTACCAGCTCCC

GGAACTGGGACTTAACGATTGGCTTGTCTTCCCGAATATGGGTGCTTATACGACGTCGTC

AGGGACCAACTTCAATGGCTTTAGCACTTCAGCTATCGCTACCTACCTTGCATGTTCCAG

TCCCATTGCGCGGGAACAAGCTATGATCGAATCAGCTGCGATGTTCGCTAATAGTATGTT

TAGTAGTTTTGCAACACCTAAACCAATAGTTTGA

gagaaagcttgtctatcttttggtttaggaaactcaataatagctgcatgggtgggggg

>SfLDC_variant_2_mirabait2_83_c3rc

aatcacagtccttcacccaaaattaaaaaaa

ATGCCTACACTAGTAACTGAGGCATTCCA

TGCCAAGGGTGCAGGACCTTTGAGCCTGAAGCCACTATTTAGTGCTTCAGGGGTTAAGGG

CAAAAGAGTCACTGCATTATCTGCAAAAGAAGAAGGTGGCATCTCTGGTTTCATCCAATC

AATCATTCACGACAAACCAGAGATGGATTCACCGTTTTTGGTGCTTGATCTTGGGGTCGT

CATGGACCTCATGGACAATTGGACCAACAACCTTCCCACAGTTCAACCTTTCTATGCAGT

TAAGTGCAACCCTAACCCATGCTTGCTGGGAGCACTGGCAGCACTCGGTTCCAGCTTCGA

CTGCGCTAGTCGAGCTGAGATCGAATCCGTTTTGTCACTGGGAGTCTCACCGGACAAAAT

CATCTACGCCAATCCATGCAAATCCGAGTCTCACATCAAATACGCTGCCAGTGTGGGTGT

CAACGTTACAACGTTTGACTCCAAAGAAGAGATCGACAAGATTCGAAAATGGCACCCGAA

ATGTGAGCTTCTCATCCGCATCAAACCCCCAGGAGACAGCGGAGCACGAAATGCGTTGGG

CCTCAAATACGGTGCGCTTCCTGAAGAAGTCATGCCTCTCCTCCAAGCTGCTCAAAACGC

GGGGTTGAAGGTCACCGGCGTGTCGTTTCATATCGGAAGTGGAGGAGCTGATTCTCAAAC

CTATCACGGAGCTATCGCTGCTGCTAAGAGAGTTTTCGACATGGCTTCTTCTGAGCTAAA

CATGCCTAGAATGAAAGTACTGGACATTGGTGGCGGTTTCACATGTGGGAAGCAGTTTGA

GGCTGCTGCATTGCACGTGAACGAGGCTCTTCAAGTTCACTTCGGAGATGAAGAGGGTGT

CGTGGTTATTGGAGAACCGGGTCGTTATTTTGCTGAGTCAGCTTTTACGTTGGCCAGTAA

AGTTATTGGGAAGCGTGTAAGAGGCGAGGTGAGGGAGTATTGGATCGACGATGGGATCTA

CGGTTCCCTGAATTGCATAATGTTTGATTTCGCGACAGTCACGTGCTCACCACTCGCGTG

CAGCTCAAAGCCTGAGAATCCCAGATGCAGAGACTCGAAAACGTACCCTTCAACTGTGTT

TGGTCCCACTTGCGATTCATTAGATACTATTTTCAGAGATTACCAGCTCCCGGAACTGGG

ACTTAACGATTGGCTTGTCTTCCCGAATATGGGTGCTTATACGACGTCGTCAGGGACCAA

CTTCAATGGCTTTAGCACTTCAGCTATCGCTACCTACCTTGCATGTTCTAGTCCCATTGC

GCGGGAACAAGCTGTGATCGAATCAGCTGCGATGTTCGCTAATAGTATGTTTAGTAGTTT

TGCAACACCTAAACCAATAGTTTGA

gagaaagcttgtctatcttttggtttaggaaactc

aataatagctgcatgggggggggagggatatgcacgcatgtttttctttttgtctgtccc

tttctatctgctactttcggaacaccwacgttc

>SfCAO_cDNA_mirabait2_81_c5

ggaaataactcataggaagaagagtaagagtgagtttccactgtatgtca

gtggattgctgtgacacgttgtggtggaattagaaagttctctcacgctt

tata

ATGGCATCAGTTTCACAAAAGGTGGCGCCACCTTCTCCTTGCTGTT

CCCCCGGCGGCGACTCAAATCACATTCCACTCCATGCTGCCGCCACTTCC

TCTGCCGAGACTCAAGACTGGACTGATACCATCTCTGACGACCGCCGCCC

CAACACGGTGGCCCTCGTTCGCCCCGTCGACTCCCTTCCTGTGCCTCCCA

CCAATGCTCCCACTGTCAAAGGAATCACTACAATGCCAAGGCCCCAGTCA

AGCCACCCTTTGGACCCTTTATCTGCTGCTGAAATCTCTGTGGCAGTGGC

AACTGTGAGGGCTGCTGGTTCCACTCCTGAGCTGAGAGACAGTATGCGCT

TCCTTGAAGTAGTTTTGGTGGAACCAGATAAACATGTTATTGCACTTGCA

GATGCTTATTTCTTCCCACCATTCCAACCATCATTACTTCATAGAACTAA

AGGAGGGCCTCTCATTCCAACTAAACTCCCTCCAAGATGTGCTAAACTTG

TTGTTTACAGTAGGAAGACAAATGAGACTTCTATATGGATTGTTGAATTA

TCACAAGCACATGCAGTAACTAGAGGTGGTCATCATAGAGGAAAAGTAAT

TGTATCACATGTTATTCCTGAGGTTCAGCCTCCAATGGATGCTGTGGAGT

ATGCAGAATGTGAGGCTGCTGTTAAAAGTTTTCCTCCATTTATAGAGGCT

ATGAAGAAAAGGGGTGTTGAAGACATGGACCTTGTAATGGTTGATCCCTG

GTGTGCAGGTTATTTCAGTGAAGCTGATGCTCCCAAGAGAAGACTTGCTA

AACCACTAATATTTTGTCGATCCGAGAGTGACTGCCCTATGGAAAATGGC

TATGCACGCCCCGTTGAGGGTATCTTTGTTCTTGTTGATATGCAAAACAT

GGTGGTGATAGAGTTTGAAGACCGCAAGCTTGTTCCTCTGCCTCCAGTTG

ATCCATTAAGGAACTATACTCGCGGTGAAACAAGAGGTGGCACTGATAGA

AGTGATGTAAAACCTTTGCAAATTATTCAACCCGAAGGTCCAAGCTTTCG

TGTCAATGGATATTATGTTGAATGGCAGAAGTGGAATTTTCGTGTTGGAT

TCACACCCAAAGAAGGTTTGGTTATATATTCTGTTGCGTATGTTGATGGT

AGTCGAGGGCGAAGACCCGTAGCTCATAGGCTGAGTTTCGTGGAGATGGT

TGTACCCTATGGAGATCCAAATGATCCACATTACAGGAAAAATGCTTTTG

ATGCCGGGGAAGATGGTTTAGGAAGAAATGCACATTCCCTCAAGAAGGGA

TGTGATTGTTTGGGTTTCATCAAATATTTTGATGCTCACTTTACAAGTTT

CACTGGTGGAGTGGAGACAATTGAGAATTGTGTGTGTATGCATGAAGAAG

ATCATGGAATTCTGTGGAAGCATCAAGATTGGAGAACTGGCTTAGCAGAA

GTCAGAAGGTCTAGAAGACTTACAGTGTCTTTTATATGTACTGTGGCTAA

CTATGAGTATGGATTTTTTTGGCACTTTTATCAGGATGGAAAGATTGAAG

CTGAAGTTAAGCTAACTGGAATCCTCAGCTTAGGAGCCTTGATGCCTGGA

GAGTATCGAAAATATGGAACCATGATTGCCCCAGGTCTGTATGCTCCAGT

TCATCAGCACTTTTTTGTTGCTCGTATGGACATGGCTGTTGATTCTAAAC

CTGGTGAAGCTTTGAATCAGGTTGTGGAGGTAAATGTGAAAATTGAGGAA

CCTGGTGAGAATAATGTTCACAATAATGCATTCTATGCTGAAGAAACTTT

GCTCAGATCTGAATTGGAAGCCGTGCGCGATTGCAATCCCATGACTGCTC

GGCATTGGATTGTAAGGAACACAAGATCAAGCAATAGAACTGGAGAGTTA

ACAGGCTACAAGCTAGTACCAGGCTCAAACTGCTTACCATTAGCAGGTTC

CGATGCCAAGTTTTTAAGAAGAGCTGCTTTCTTGAAGCATAATCTTTGGG

TAACAGCTTATTCACCTGATGAGATGTTTCCTGGAGGAGAATTTCCTAAT

CAAAATCCACGTATTGGCGAAGGATTACCTACATGGGTTAAGCAGAACCG

GTCTTTAGAAGAGACTAATATAGTTCTTTGGTATGTATTTGGAGTCACAC

ATGTTCCTCGTTTAGAAGACTGGCCTGTTATGCCAGTAGAGCACATTGGT

TTTATGCTCATGCCTCATGGATTCTTCAATTGTTCCCCTGCGATAGATGT

GCCACCTAATCCATGTGAATTGGATTCTAAAGATAATGACATCAAGGACA

ATGGTGCTTTGAAGCCAATTCAGAGTGCGTTAGCGGCAAAGCTTTAG

gaacctttcgcaccaaaagttatggcaatgtgctgccgagagaaacacgaata

ctattttggcatccacttgagcagattatctttttcaatatagaataata

atcaagctatggacttaaataaagcgagcatgtattgttcattcgatttg

cctccgaaccttggag

>SfTUB_mirabait2_81_83_c5_c4_confident

NCCAAGCGCACCATTCAGTTTGTGGATTGGTGCCC

AACTGGTTTCAAGTGTGGTATCAACTACCAACCACCTACTGTTGTTCCTGGGGGTGACCT

TGCCAAGGTGCAGAGGGCAGTTTGCATGATTTCGAACTCCACTAGTGTGGCTGAGGTGTT

TTCTCGCATTGACCACAAGTTTGACCTCATGTATTCCAAACGTGCTTTTGTTCACTGGTA

TGTGGGTGAGGGTATGGAAGAAGGTGAATTTTCTGAAGCTCGTGAGGATCTTGCTGCCCT

CGAAAAGGATTATGAAGAAGTGGGTGCTGAGTCCACTGAAGGTGGAGAGGATGATGAAGA

CTACTAGattttccattccaattctggtggcctttgttttatcgtatcgatggttatggtgtg

tgtagatcggcatggttgtgagcttttatcggtttgttcatttgtacgggacattgtttt

gatgccccgtggttgtttgtgttgtatttttaaaccttgacgtaatttctattttctaat

tacatccctcctattcgcgttctct

>SfCYC_mirabait2_81_83_onemismatch_qPCRdesign

NTGTGCCAGGGCGGCGACTTCACCGCCGGAAACGGCACCGGAGGCGAATCGATCTACGGAG

CGAAGTTCGCCGACGAGAACTTCATCAAGAAGCACACCGGTCCCGGCATCTTGTCGATGG

CGAATGCTGGACCTGGGACCAATGGATCTCAGTTCTTCATCTGCACGACGAAGACGGAGT

GGCTCGACGGAAAGCACGTGGTGTTCGGTCAGGTGGTTGATGGAATCAATGTGGTGAAAG

AGATCGAGAAGGTTGGATCCAGCTCCGGCAAGACCTCGAGGCCTGTGGTGGTTGCCGATT

GCGGTCAACTCTCTTAGacggtggtgttgaccgtccgtacatggtggctcttatctaaac

cctttgtgtcgtcttgtctatgcggtgtcgtttttgctttttatatttgtccaagtcgtt

ttaggaactgtggtgctatctttggaggagtggatcgattgatcatat

>SfEFA_mirabait2_81_c10

gcctctcaggcttcccttgcaggatgtctacaagattggtggtattggta

ctgtcccactggcacagtaccaataccaccaatcttgtagacatcctgca

agggaagcctgagaggccctcagacaagcctctcaggcttcccttgcrgg

atgtctacaagattggtggtattggtactggcacagtaccaataccacca

atcttgtagacatcctgcaagggaagcctgcaggatgtgtacaagattgg

tggtattgggactgtgccagttggacgtgttgaaactggtgtcataaagc

caggtATGGTGGTGACCTTTGGTCCCACTGGTCTGACAACTGAAGTTAAG

TCTGTGGAGATGCACCATGAGGCTCTCACAGAGGCTCTTCCAGGTGACAA

TGTGGGATTTAATGTGAAGAATGTTGCAGTCAAGGATCTCAAGCGTGGTT

TTGTTGCCTCAAACTCTAAGGATGACCCTGCTAAGGAGGCTGCCAACTTC

ACATCCCAAGTCATTATAATGAACCATCCTGGCCAGATTGGGAATGGCTA

TGCCCCTGTGCTTGACTGCCACACTTCTCACATTGCTGTCAAGTTTGCTG

AGCTTTTGACCAAGATTGACAGGCGATCTGGGAAGGAGCTTGAGAAGGAA

CCCAAATTCTTGAAGAATGGAGATGCAGGTCTAGTTAAGATGATTCCCAC

CAAGCCTATGGTGGTTGAAACTTTCTCTGAGTATCCTCCTCTTGGTCGTT

TTGCTGTGAGGGACATGCGTCAAACTGTGGCTGTTGGAGTTATTAAGAGT

GTGGAGAAGAAGGACCCCACTGGAGCCAAGGTCACCAAGGCGGCAGCAAA

GAAGAAGTGA

atgtatcttggtatttctgcaagtgatcataattatggtt

acagaaaacagtggttccttgttagtaatttatcctttgagattctaggt

gtttttacctgttatagtttcaactgtcacccgttttcat

>SfUBQb_60S_RP_mb2_83_tf_c2_CDS_387nt_641nt

ctagaagtggaaatgcaagtataaaaccacttgttggctgtaacatttca

cggcgcagagagcagagaagaagcagcagcaaag

ATGCAGATTTTCGTGA

AAACCCTAACTGGGAAGACCATTACCCTCGAGGTCGAAAGCAGCGACACC

ATCGACAATGTCAAAGCTAAGATCCAAGACAAGGAAGGCATTCCACCGGA

TCAACAACGTTTGATATTCGCTGGGAAGCAGCTTGAGGATGGAAGAACAT

TAGCTGATTACAACATTCAGAAGGAATCGACTCTTCACCTTGTTCTCAGG

CTTCGTGGTGGAATCATTGAGCCTTCTTTGATGGCTTTGGCTCGTAAATA

CAACCAAGACAAGATGATCTGCCGCAAATGCTACGCTCGTCTACATCCTA

GGGCTGTGAACTGCCGCAAAAAGAAGTGTGGTCACAGTAATCAGTTGAGG

CCAAAGAAGAAGATCAAGTAG

attctagctgttatggttcctctcttttt

gatgttgcaattcagatcattattgctatctatggttttgattaatgttt

aaattgtttcggaatttacatttcggcagtaattatattgttaggatgac

tttatgatctctttrcagtaatcagttgaggccacagaaga

**(B)** *SfLDC* gDNA sequence

>*SfLDC_*1_12

aacatcccagttgaaatcacagtccttcacccaaaattaataaaaAT

GCCTACACTAGTAACTGAGGCATTCCATGCCAAGGGTGCAGGACCTTTGA

GCCTGAAGCCACTATTTAGTGCTTCAGGGGTTAAGGGCAAAAGAGTCACT

GCATTATCTGCAAAAGAAGAAGGTGGCATCTCTGGTTTCATCCAATCAAT

CATTCACGACAAACCAGAGATGGATTCACCGTTTTTGGTGCTTGATCTTG

GGGTCGTCATGGACCTCATGGACAATTGGACCAACAACCTTCCCACAGTT

CAACCTTTCTATGCAGTTAAGTGCAACCCTAACCCATGCTTGCTGGGAGC

ACTGGCAGCACTCGGTTCCAGCTTCGACTGCGCTAGTCGAGCTGAGATCG

AATCCGTTTTGTCACTGGGAGTCTCACCGGACAAAATCATCTACGCCAAT

CCATGCAAATCCGAGTCTCACATCAAATACGCTGCCAGTGTGGGTGTCAA

CGTTACAACGTTTGACTCCAAAGAAGAGATCGACAAGATTCGAAAATGGC

ACCCGAAATGTGAGCTTCTCATCCGCATCAAACCCCCAGGAGACAGCGGA

GCACGAAATGCGTTGGGCCTCAAATACGGTGCGCTTCCTGAAGAAGTCAT

GCCTCTCCTCCAAGCTGCTCAAAACGCGGGGTTGAAGGTCACCGGCGTGT

CGTTTCATATCGGAAGTGGAGGAGCTGATTCTCAAACCTATCACGGAGCT

ATCGCTGCTGCTAAGAGAGTTTTCGACATGGCTTCTTCTGAGCTAAACAT

GCCTAGAATGAAAGTACTGGACATTGGTGGCGGTTTCACATGTGGGAAGC

AGTTTGAGGCTGCTGCATTGCACGTGAACGAGGCTCTTCAAGTTCACTTC

GGAGATGAAGAGGGTGTCGTGGTTATTGGAGAACCGGGTCGTTATTTTGC

TGAGTCAGCTTTTACGTTGGCCAGTAAAGTTATTGGGAAGCGTGTAAGAG

GCGAGGTGAGGGAGTATTGGATCGACGATGGGATCTACGGTTCCCTGAAT

TGCATAATGTTTGATTTCGCGACAGTCACGTGCTCACCACTCGCGTGCAG

CTCAAAGCCTGAGAATCCCAGATGCAGAGACTCGAAAACGTACCCTTCAA

CTGTGTTTGGTCCCACTTGCGATTCATTAGATACTATTTTCAGAGATTAC

CAGCTCCCGGAACTGGGACTTAACGATTGGCTTGTCTTCCCGAATATGGG

TGCTTATACGACGTCGTCAGGGACCAACTTCAATGGCTTTAGCACTTCAG

CTATCGCTACCTACCTTGCATGTTCCAGTCCCATTGCGCGGGAACAAGCT

ATGATCGAATCAGCTGCGATGTTCGCTAATAGTATGTTTAGTAGTTTTGC

AACACCTAAACCAATAGTTTGAgagaaag

>*SfLDC_*2_11

aacatcccagttgaaatcacagtccttcacccaaaattaaaaaaaATGCC

TACACTAGTAACTGAGGCATTCCATGCCAAGGGTGCAGGACCTTTGAGCC

TGAAGCCACTATTTAGTGCTTCAGGGGTTAAGGGCAAAAGAGTCACTGCA

TTATCTGCAAAAGAAGAAGGTGGCATCTCTGGTTTCATCCAATCAATCAT

TCACGACAAACCAGAGATGGATTCACCGTTTTTGGTGCTTGATCTTGGGG

TCGTCATGGACCTCATGGACAATTGGACCAACAACCTTCCCACAGTTCAA

CCTTTCTATGCAGTTAAGTGCAACCCTAACCCATGCTTGCTGGGAGCACT

GGCAGCACTCGGTTCCAGCTTCGACTGCGCTAGTCGAGCTGAGATCGAAT

CCGTTTTGTCACTGGGAGTCTCACCGGACAAAATCATCTACGCCAATCCA

TGCAAATCCGAGTCTCACATCAAATACGCTGCCAGTGTGGGTGTCAACGT

TACAACGTTTGACTCCAAAGAAGAGATCGACAAGATTCGAAAATGGCACC

CGAAATGTGAGCTTCTCATCCGCATCAAACCCCCAGGAGACAGCGGAGCA

CGAAATGCGTTGGGCCTCAAATACGGTGCGCTTCCTGAAGAAGTCATGCC

TCTCCTCCAAGCTGCTCAAAACGCGGGGTTGAAGGTCACCGGCGTGTCGT

TTCATATCGGAAGTGGAGGAGCTGATTCTCAAACCTATCACGGAGCTATC

GCTGCTGCTAAGAGAGTTTTCGACATGGCTTCTTCTGAGCTAAACATGCC

TAGAATGAAAGTACTGGACATTGGTGGCGGTTTCACATGTGGGAAGCAGT

TTGAGGCTGCTGCATTGCACGTGAACGAGGCTCTTCAAGTTCACTTCGGA

GATGAAGAGGGTGTCGTGGTTATTGGAGAACCGGGTCGTTATTTTGCTGA

GTCAGCTTTTACGTTGGCCAGTAAAGTTATTGGGAAGCGTGTAAGAGGCG

AGGTGAGGGAGTATTGGATCGACGATGGGATCTACGGTTCCCTGAATTGC

ATAATGTTTGATTTCGCGACAGTCACGTGCTCACCACTCGCGTGCAGCTC

AAAGCCTGAGAATCCCAGATGCAGAGACTCGAAAACGTACCCTTCAACTG

TGTTTGGTCCCACTTGCGATTCATTAGATACTATTTTCAGAGATTACCAG

CTCCCGGAACTGGGACTTAACGATTGGCTTGTCTTCCCGAATATGGGTGC

TTATACGACGTCGTCAGGGACCAACTTCAATGGCTTTAGCACTTCAGCTA

TCGCTACCTACCTTGCATGTTCCAGTCCCATTGCGCGGGAACAAGCTATG

ATCGAATCAGCTGCGATGTTCGCTAATAGTATGTTTAGTAGTTTTGCAAC

ACCTAAACCAATAGTTTGAgagaaag

>*SfLDC_*3_5

aacatcccagttgaaatcacagtccttcacccaaaattaataaaaATGCC

TACACTAGTAACTGAGGCATTCCATGCCAAGGGTGCAGGACCTTTGAGCC

TGAAGCCACTATTTAGTGCTTCAGGGGTTAAGGGCAAAAGAGTCACTGCA

TTATCTGCAAAAGAAGAAGGTGGCATCTCTGGTTTCATCCAATCAATCAT

TCACGACAAACCAGAGATGGATTCACCGTTTTTGGTGCTTGATCTTGGGG

TCGTCATGGACCTCATGGACAATTGGACCAACAACCTTCCCACAGTTCAA

CCTTTCTATGCAGTTAAGTGCAACCCTAACCCATGCTTGCTGGGAGCACT

GGCAGCACTCGGTTCCAGCTTCGACTGCGCTAGTCGAGCTGAGATCGAAT

CCGTTTTGTCACTGGGAGTCTCACCGGACAAAATCATCTACGCCAATCCA

TGCAAATCCGAGTCTCACATCAAATACGCTGCCAGTGTGGGTGTCAACGT

TACAACGTTTGACTCCAAAGAAGAGATCGACAAGATTCGAAAATGGCACC

CGAAATGTGAGCTTCTCATCCGCATCAAACCCCCAGGAGACAGCGGAGCA

CGAAATGCGTTGGGCCTCAAATACGGTGCGCTTCCTGAAGAAGTCATGCC

TCTCCTCCAAGCTGCTCAAAACGCGGGGTTGAAGGTCACCGGCGTGTCGT

TTCATATCGGAAGTGGAGGAGCTGATTCTCAAACCTATCACGGAGCTATC

GCTGCTGCTAAGAGAGTTTTCGACATGGCTTCTTCTGAGCTAAACATGCC

TAGAATGAAAGTACTGGACATTGGTGGCGGTTTCACATGTGGGAAGCAGT

TTGAGGCTGCTGCATTGCACGTGAACGAGGCTCTTCAAGTTCACTTCGGA

GATGAAGAGGGTGTCGTGGTTATTGGAGAACCGGGTCGTTATTTTGCTGA

GTCAGCTTTTACGTTGGCCAGTAAAGTTATTGGGAAGCGTGTAAGAGGCG

AGGTGAGGGAGTATTGGATCGACGATGGGATCTACGGTTCCCTGAATTGC

ATAATGTTTGATTTCGCGACAGTCACGTGCTCACCACTCGCGTGCAGCTC

AAAGCCTGAGAATCCCAGATGCAGAGACTCGAAAACGTACCCTTCAACTG

TGTTTGGTCCCACTTGCGATTCATTAGATACTATTTTCAGAGATTACCAG

CTCCCGGAACTGGGACTTAACGATTGGCTTGTCTTCCCGAATATGGGTGC

TTATACGACGTCGTCAGGGACCAACTTCAATGGCTTTAGCACTTCAGCTA

TCGCTACCTACCTTGCATGTTCCAGTCCCATTGCGCGGGAACAAGCTATG

ATCGAATCAGCTGCGATGTTCGCTAATAGTATGTTTAGTAGTTTTGCAAC

ACCTAAACCAATAGTTTGAgagaaag

>*SfLDC_*4_15

aacatcccagttgaaatcacagtccttcacccaaaattaataaaaATGCC

TACACTAGTAACTGAGGCATTCCATGCCAAGGGTGCAGGACCTTTGAGCC

TGAAGCCACTATTTAGTGCTTCAGGGGTTAAGGGCAAAAGAGTCACTGCA

TTATCTGCAAAAGAAGAAGGTGGCATCTCTGGTTTCATCCAATCAATCAT

TCACGACAAACCAGAGATGGATTCACCGTTTTTGGTGCTTGATCTTGGGG

TCGTCATGGACCTCATGGACAATTGGACCAACAACCTTCCCACAGTTCAA

CCTTTCTATGCAGTTAAGTGCAACCCTAACCCATGCTTGCTGGGAGCACT

GGCAGCACTCGGTTCCAGCTTCGACTGCGCCAGTCGAGCTGAGATCGAAT

CCGTTTTGTCACTTGGAGTCTCACCGGACAAAATCATCTACGCCAATCCA

TGCAAATCMGAGTCTCACATCAAATACGCTGCCAGTGTGGGTGTCAACGT

TACAACGTTTGACTCCAAAGAAGAGATCGACAAGATTCGAAAATGGCACC

CGAAATGTGAGCTTCTCATCCGCATCAAACCCCCAGGAGACAGCGGAGCA

CGAAATGCGTTGGGCCTCAAATACGGTGCGCTTCCTGAAGAAGTCATGCC

TCTCCTCCAAGCTGCTCAAAACGCGGGGTTGAAGGTCACCGGCGTGTCGT

TTCATATCGGAAGTGGAGGAGCTGATTCTCAAACCTATCACGGAGCTATC

GCTGCTGCTAAGAGAGTTTTCGACATGGCTTCTTCTGAGCTAAACATGCC

TAGAATGAAAGTACTGGACATTGGTGGCGGTTTCACATGTGGGAAGCAGT

TTGAGGCTGCTGCATTGCACGTGAACGAGGCTCTTCAAGTTCACTTCGGA

GATGAAGAGGGTGTCGTGGTTATTGGAGAACCGGGTCGTTATTTTGCTGA

GTCAGCTTTTACGTTGGCCAGTAAAGTTATTGGGAAGCGTGTAAGAGGCG

AGGTGAGGGAGTATTGGATCGACGATGGGATCTACGGTTCCCTGAATTGC

ATAATGTTTGATTTCGCGACAGTCACGTGCTCACCACTCGCGTGCAGCTC

AAAGCCTGAGAATCCCAGATGCAGAGACTCGAAAACGTACCCTTCAACTG

TGTTTGGTCCCACTTGYGATTCRTTAGATACTATTTTCAGAGATTACCAG

CTCCCGGAACTGGRACTTAACGATTGGCTTGTCTTCCCGAATATGGGTGC

TTATACGACGTCGTCAGGGACCAACTTCAATGGCTTTAGCACTTCAGCTA

TCGCTACCTACCTTGCATGTTCCAGTCCCATTGGGCGGGAACAAGCTATG

ATCGAATCAGCTGTGATGTTCGCTAATAGTATGTTTAGTAGTTTTGCAAC

ACCTAAACCAATAGTTTGAgagaaag

>*SfLDC_*5_13

aacatcccagttgaaatcacagtccttcacccaaaattaataaaaATGCC

TACACTAGTAACTGAGGCATTCCATGCCAAGGGTGCAGGACCTTTGAGCC

TGAAGCCACTATTTAGTGCTTCAGGGGTTAAGGGCAAAAGAGTCACTGCA

TTATCTGCAAAAGAAGAAGGTGGCATCTCTGGTTTCATCCAATCAATCAT

TCACGACAAACCAGAGATGGATTCACCGTTTTTGGTGCTTGATCTTGGGG

TCGTCATGGACCTCATGGACAATTGGACCAACAACCTTCCCACAGTTCAA

CCTTTCTATGCAGTTAAGTGCAACCCTAACCCATGCTTGCTGGGAGCACT

GGCAGCACTCGGTTCCAGCTTCGACTGCGCCAGTCGAGCTGAGATCGAAT

CCGTTTTGTCACTTGGAGTCTCACCGGACAAAATCATCTACGCCAATCCA

TGCAAATCCGAGTCTCACATCAAATACGCTGCCAGTGTGGGTGTCAACGT

TACAACGTTTGACTCCAAAGAAGAGATCGACAAGATTCGAAAATGGCACC

CGAAATGCGAGCTTCTCATCCGCATCAAACCCCCAGGAGACAGCGGAGCA

CGAAATGCGTTGGGCCTCAAATACGGTGCGCTTCCTGAAGAAGTCATGCC

TCTCCTCCAAGCTGCTCAAAACGCGGGGTTGAAGGTCACCGGCGTGTCGT

TTCATATCGGAAGTGGAGGAGCTGATTCTCAAACCTATCACGGAGCTATC

GCTGCTGCTAAGAGAGTTTTCGACATGGCTTCTTCTGAGCTAAACATGCC

TAGAATGAAAGTACTGGACATTGGTGGCGGTTTCACATGTGGGAAGCAGT

TTGAGGCTGCTGCATTGCACGTGAACGAGGCTCTTCAAGTTCACTTCGGA

GATGAAGAGGGTGTCGTGGTTATTGGAGAACCGGGTCGTTATTTTGCTGA

GTCAGCTTTTACGTTGGCCAGTAAAGTTATTGGGAAGCGTGTAAGAGGCG

AGGTGAGGGAGTATTGGATCGACGATGGGATCTACGGTTCCCTGAATTGC

ATAATGTTTGATTTCGCGACAGTCACGTGCTCACCACTCGCGTGCAGCTC

AAAGCCTGAGAATCCCAGATGCAGAGACTCGAAAACGTACCCTTCAACTG

TGTTTGGTCCCACTTGCGATTCATTAGATACTATTTTCAGAGATTACCAG

CTCCCGGAACTGGGACTTAACGATTGGCTTGTCTTCCCGAATATGGGTGC

TTATACGACGTCGTCAGGGACCAACTTCAATGGCTTTAGCACTTCAGCTA

TCGCTACCTACCTTGCATGTTCCAGTCCCATTGCGCGGGAACAAGCTATG

ATCGAATCAGCTGCGATGTTCGCTAATAGTATGTTTAGTAGTTTTGCAAC

ACCTAAACCAATAGTTTGAgagaaag

>*SfLDC_*6_16

aacatcccagttgaaatcacagtccttcacccaaaattaataaaaATGCC

TACACTAGTAACTGAGGCATTCCATGCCAAGGGTGCAGGACCTTTGAGCC

TGAAGCCACTATTTAGTGCTTCAGGGGTTAAGGGCAAAAGAGTCACTGCA

TTATCTGCAAAAGAAGAAGGTGGCATCTCTGGTTTCATCCAATCAATCAT

TCACGACAAACCAGAGATGGATTCACCGTTTTTGGTGCTTGATCTTGGGG

TCGTCATGGACCTCATGGACAATTGGACCAACAACCTTCCCACAGTTCAA

CCTTTCTATGCAGTTAAGTGCAACCCTAACCCATGCTTGCTGGGAGCACT

GGCAGCACTCGGTTCCAGCTTCGACTGCGCCAGTCGAGCTGAGATCGAAT

CCGTTTTGTCACTTGGAGTCTCACCGGACAAAATCATCTACGCCAATCCA

TGCAAATCAGAGTCTCACATCAAATACGCTGCCAGTGTGGGTGTCAACGT

TACAACGTTTGACTCCAAAGAAGAGATCGACAAGATTCGAAAATGGCACC

CGAAATGTGAGCTTCTCATCCGCATCAAACCCCCAGGAGACAGCGGAGCA

CGAAATGCGTTGGGCCTCAAATACGGTGCGCTTCCTGAAGAAGTCATGCC

TCTCCTCCAAGCTGCTCAAAACGCGGGGTTGAAGGTCACCGGCGTGTCGT

TTCATATCGGAAGTGGAGGAGCTGATTCTCAAACCTATCACGGAGCTATC

GCTGCTGCTAAGAGAGTTTTCGACATGGCTTCTTCTGAGCTAAACATGCC

TAGAATGAAAGTACTGGACATTGGTGGCGGTTTCACATGTGGGAAGCAGT

TTGAGGCTGCTGCATTGCACGTGAACGAGGCTCTTCAAGTTCACTTCGGA

GATGAAGAGGGTGTCGTGGTTATTGGAGAACCGGGTCGTTATTTTGCTGA

GTCAGCTTTTACGTTGGCCAGTAAAGTTATTGGGAAGCGTGTAAGAGGCG

AGGTGAGGGAGTATTGGATCGACGATGGGATCTACGGTTCCCTGAATTGC

ATAATGTTTGATTTCGCGACAGTCACGTGCTCACCACTCGCGTGCAGCTC

AAAGCCTGAGAATCCCAGATGCAGAGACTCGAAAACGTACCCTTCAACTG

TGTTTGGTCCCACTTGCGATTCATTAGATACTATTTTCAGAGATTACCAG

CTCCCGGAACTGGGACTTAACGATTGGCTTGTCTTCCCGAATATGGGTGC

TTATACGACGTCGTCAGGGACCAACTTCAATGGCTTTAGCACTTCAGCTA

TCGCTACCTACCTTGCATGTTCCAGTCCCATTGCGCGGGAGCAAGCTATG

ATCGAATCAGCTGCGATGTTCGCTAATAGTATGTTTAGTAGTTTTGCAAC

ACCTAAACCAATAGTTTGAgagaaag

>*SfLDC_*7_10

aacatcccagttgaaatcacagtccttcacccaaaattaawaaaaAT

GCCTACACTAGTAACTGAGGCATTCCATGCCAAGGGTGCAGGACCTTTGA

GCCTGAAGCCACTATTTAGTGCTTCAGGGGTTAAGGGCAAAAGAGTCACT

GCATTATCTGCAAAAGAAGAAGGTGGCATCTCTGGTTTCATCCAATCAAT

CATTCACGACAAACCAGAGATGGATTCACCGTTTTTGGTGCTTGATCTTG

GGGTCGTCATGGACCTCATGGACAATTGGACCAACAACCTTCCCACAGTT

CAACCTTTCTATGCAGTTAAGTGCAACCCTAACCCATGCTTGCTGGGAGC

ACTGGCAGCACTCGGTTCCAGCTTCGACTGCGCCAGTCGAGCTGAGATCG

AATCCGTTTTGTCACTTGGAGTCTCACCGGACAAAATCATCTACGCCAAT

CCATGCAAATCMGAGTCTCACATCAAATACGCTGCCAGTGTGGGTGTCAA

CGTTACAACGTTTGACTCCAAAGAAGAGATCGACAAGATTCGAAAATGGC

ACCCGAAATGTGAGCTTCTCATCCGCATCAAACCCCCAGGAGACAGCGGA

GCACGAAATGCGTTGGGCCTCAAATACGGTGCGCTTCCTGAAGAAGTCAT

GCCTCTCCTCCAAGCTGCTCAAAACGCGGGGTTGAAGGTCACCGGCGTGT

CGTTTCATATCGGAAGTGGAGGAGCTGATTCTCAAACCTATCACGGAGCT

ATCGCTGCTGCTAAGAGAGTTTTCGACAYGGCTTCTTCTGAGCTAAACAT

GCCTAGAATGAAAGTACTGGACATTGGTGGCGGTTTCACATGTGGGAAGC

AGTTTGAGGCTGCTGCATTGCACGTGAACGAGGCTCTTCRAGTTCACTTC

GGAGATGAAGAGGGTGTCGTGGTTATTGGAGAACCGGGTCGTTATTTTGC

TGAGTCAGCTTTTACGTTGGCCAGTAAAGTTATTGGGAAGCGTGTAAGAG

GCGAGGTGAGGGAGTATTGGATCGACGATGGGATCTACGGTTCCCTGAAT

TGCATAATGTTTGATTTCGCGACAGTCACGTGCTCACCACTCGCGTGCAG

CTCAAAGCCTGAGAATCCCAGATGCAGAGACTCGAAAACGTACCCTTCAA

CTGTGTTTGGTCCCACTTGCGATTCATTAGATACTATTTTCAGAGATTAC

CAGCTCCCGGAACTGGGACTTAACGATTGGCTTGTCTTCCCGAATATGGG

TGCTTATACGACGTCGTCAGGGACCAACTTCAATGGCTTTAGCACTTCAG

CTATCGCTACCTACCTTGCATGTTCCAGTCCCATTGCGCGGGARCAAGCT

ATGATCGAATCAGCTGCGATGTTCGCTAATAGTATGTTTAGTAGTTTTGC

AACACCTAAACCAATAGTTTGAgagaaag

>*SfLDC_*8_11

aacatcccagttgaaatcacagtccttcacccaaaattaataaaaATGCC

TACACTAGTAACTGAGGCATTCCATGCCAAGGGTGCAGGACCTTTGAGCC

TGAAGCCACTATTTAGTGCTTCAGGGGTTAAGGGCAAAAGAGTCACTGCA

TTATCTGCAAAAGAAGAAGGTGGCATCTCTGGTTTCATCCAATCAATCAT

TCACGACAAACCAGAGATGGATTCACCGTTTTTGGTGCTTGATCTTGGGG

TCGTCATGGACCTCATGGACAATTGGACCAACAACCTTCCCACAGTTCAA

CCTTTCTATGCAGTTAAGTGCAACCCTAACCCATGCTTGCTGGGAGCACT

GGCAGCACTCGGTTCCAGCTTCGACTGCGCTAGTCGAGCTGAGATCGAAT

CCGTTTTGTCACTGGGAGTCTCACCGGACAAAATCATCTACGCCAATCCA

TGCAAATCCGAGTCTCACATCAAATACGCTGCCAGTGTGGGTGTCAACGT

TACAACGTTTGACTCCAAAGAAGAGATCGACAAGATTCGAAAATGGCACC

CGAAATGTGAGCTTCTCATCCGCATCAAACCCCCAGGAGACAGCGGAGCA

CGAAATGCGTTGGGCCTCAAATACGGTGCGCTTCCTGAAGAAGTCATGCC

TCTCCTCCAAGCTGCTCAAAACGCGGGGTTGAAGGTCACCGGCGTGTCGT

TTCATATCGGAAGTGGAGGAGCTGATTCTCAAACCTATCACGGAGCTATC

GCTGCTGCTAAGAGAGTTTTCGACATGGCTTCTTCTGAGCTAAACATGCC

TAGAATGAAAGTACTGGACATTGGTGGCGGTTTCACATGTGGGAAGCAGT

TTGAGGCTGCTGCATTGCACGTGAACGAGGCTCTTCAAGTTCACTTCGGA

GATGAAGAGGGTGTCGTGGTTATTGGAGAACCGGGTCGTTATTTTGCTGA

GTCAGCTTTTACGTTGGCCAGTAAAGTTATTGGGAAGCGTGTAAGAGGCG

AGGTGAGGGAGTATTGGATCGACGATGGGATCTACGGTTCCCTGAATTGC

ATAATGTTTGATTTCGCGACAGTCACGTGCTCACCACTCGCGTGCAGCTC

AAAGCCTGAGAATCCCAGATGCAGAGACTCGAAAACGTACCCTTCAACTG

TGTTTGGTCCCACTTGCGATTCATTAGATACTATTTTCAGAGATTACCAG

CTCCCGGAACTGGGACTTAACGATTGGCTTGTCTTCCCGAATATGGGTGC

TTATACGACGTCGTCAGGGACCAACTTCAATGGCTTTAGCACTTCAGCTA

TCGCTACCTACCTTGCATGTTCCAGTCCCATTGCGCGGGAACAAGCTATG

ATCGAATCAGCTGCGATGTTCGCTAATAGTATGTTTAGTAGTTTTGCAAC

ACCTAAACCAATAGTTTGAgagaaag

>*SfLDC_*9_8

aacatcccagttgaaatcacagtccttcacccaaaattaaaaaaaATGCC

TACACTAGTAACTGAGGCATTCCATGCCAAGGGTGCAGGACCTTTGAGCC

TGAAGCCACTATTTAGTGCTTCAGGGGTTAAGGGCAAAAGAGTCACTGCA

TTATCTGCAAAAGAAGAAGGTGGCATCTCTGGTTTCATCCAATCAATCAT

TCACGACAAACCAGAGATGGATTCACCGTTTTTGGTGCTTGATCTTGGGG

TCGTCATGGACCTCATGGACAATTGGACCAACAACCTTCCCACAGTTCAA

CCTTTCTATGCAGTTAAGTGCAACCCTAACCCATGCTTGCTGGGAGCACT

GGCAGCACTCGGTTCCAGCTTCGACTGCGCTAGTCGAGCTGAGATCGAAT

CCGTTTTGTCACTGGGAGTCTCACCGGACAAAATCATCTACGCCAATCCA

TGCAAATCCGAGTCTCACATCAAATACGCTGCCAGTGTGGGTGTCAACGT

TACAACGTTTGACTCCAAAGAAGAGATCGACAAGATTCGAAAATGGCACC

CGAAATGTGAGCTTCTCATCCGCATCAAACCCCCAGGAGACAGCGGAGCA

CGAAATGCGTTGGGCCTCAAATACGGTGCGCTTCCTGAAGAAGTCATGCC

TCTCCTCCAAGCTGCTCAAAACGCGGGGTTGAAGGTCACCGGCGTGTCGT

TTCATATCGGAAGTGGAGGAGCTGATTCTCAAACCTATCACGGAGCTATC

GCTGCTGCTAAGAGAGTTTTCGACATGGCTTCTTCTGAGCTAAACATGCC

TAGAATGAAAGTACTGGACATTGGTGGCGGTTTCACATGTGGGAAGCAGT

TTGAGGCTGCTGCATTGCACGTGAACGAGGCTCTTCAAGTTCACTTCGGA

GATGAAGAGGGTGTCGTGGTTATTGGAGAACCGGGTCGTTATTTTGCTGA

GTCAGCTTTTACGTTGGCCAGTAAAGTTATTGGGAAGCGTGTAAGAGGCG

AGGTGAGGGAGTATTGGATCGACGATGGGATCTACGGTTCCCTGAATTGC

ATAATGTTTGATTTCGCGACAGTCACGTGCTCACCACTCGCGTGCAGCTC

AAAGCCTGAGAATCCCAGATGCAGAGACTCGAAAACGTACCCTTCAACTG

TGTTTGGTCCCACTTGTGATTCGTTAGATACTATTTTCAGAGATTACCAG

CTCCCGGAACTGGAACTTAACGATTGGCTTGTCTTCCCGAATATGGGTGC

TTATACGACGTCGTCAGGGACCAACTTCAATGGCTTTAGCACTTCAGCTA

TCGCTACCTACCTTGCATGTTCCAGTCCCATTGGGCGGGAACAAGCTATG

ATCGAATCAGCTGTGATGTTCGCTAATAGTATGTTTAGTAGTTTTGCAAC

ACCTAAACCAATAGTTTGAgagaaag

>*SfLDC_*10_4

aacatcccagttgaaatcacagtccttcacccaaaattaawaaaaATGCC

TACACTAGTAACTGAGGCATTCCATGCCAAGGGTGCAGGACCTTTGAGCC

TGAAGCCACTATTTAGTGCTTCAGGGGTTAAGGGCAAAAGAGTCACTGCA

TTATCTGCAAAAGAAGAAGGTGGCATCTCTGGTTTCATCCAATCAATCAT

TCACGACAAACCAGAGATGGATTCACCGTTTTTGGTGCTTGATCTTGGGG

TCGTCATGGACCTCATGGACAATTGGACCAACAACCTTCCCACAGTTCAA

CCTTTCTATGCAGTTAAGTGCAACCCTAACCCATGCTTGCTGGGAGCACT

GGCAGCACTCGGTTCCAGCTTCGACTGCGCYAGTCGAGCTGAGATCGAAT

CCGTTTTGTCACTKGGAGTCTCACCGGACAAAATCATCTACGCCAATCCA

TGCAAATCCGAGTCTCACATCAAATACGCTGCCAGTGTGGGTGTCAACGT

TACAACGTTTGACTCCAAAGAAGAGATCGACAAGATTCGAAAATGGCACC

CGAAATGTGAGCTTCTCATCCGCATCAAACCCCCAGGAGACAGCGGAGCA

CGAAATGCGTTGGGCCTCAAATACGGTGCGCTTCCTGAAGAAGTCATGCC

TCTCCTCCAAGCTGCTCAAAACGCGGGGTTGAAGGTCWCCGGCGTGTCGT

TTCATATCGGAAGTGGAGGAGCTGATTCTCAAACCTATCACGGAGCTATC

GCTGCTGCTAAGAGAGTTTTCGACATGGCTTCTTCTGAGCTAAACATGCC

TAGAATGAAAGTACTGGACATTGGTGGCGGTTTCACATGTGGGAAGCAGT

TTGAGGCTGCTGCATTGCACGTGAACGAGGCTCTTCAAGTTCACTTCGGA

GATGAAGAGGGTGTCGTGGTTATTGGAGAACCGGGTCGTTATTTTGCTGA

GTCAGCTTTTACGTTGGCCAGTAAAGTTATTGGGAAGCGTGTAAGAGGCG

AGGTGAGGGAGTATTGGATCGACGATGGGATCTACGGTTCCCTGAATTGC

ATAATGTTTGATTTCGCGACAGTCACGTGCTCACCACTCGCGTGCAGCTC

AAAGCCTGAGAATCCCAGATGCAGAGACTCGAAAACGTACCCTTCAACTG

TGTTTGGTCCCACTTGCGATTCATTAGATACTATTTTCAGAGATTACCAG

CTCCCGGAACTGGGACTTAACGATTGGCTTGTCTTCCCGAATATGGGTGC

TTATACGACGTCGTCAGGGACCAACTTCAATGGCTTTAGCACTTCAGCTA

TCGCTACCTACCTTGCATGTTCCAGTCCCATTGCGCGGGARCAAGCTATG

ATCGAATCAGCTGCGATGTTCGCTAATAGTATGTTTAGTAGTTTTGCAAC

ACCTAAACCAATAGTTTGAgagaaag

**(C)** *SfCAO* 5' gDNA sequence

>*SfCAO*_1_12_partial_5prime

tggattgctgtgacacgttgtggtggaattagaaagttctctcacgcttt

ataATGGCATCAGTTTCACAAAAGGTGGCGYCACCTTCTCCTTGCTGTTC

CCCCGGCGGCGACTCTAATCACATTCCACTCCATGCTGCCGCCACTTCCT

CTGCCGAGACTCAAGACTGGACTGATACCATCTCTGACGACCGCCGCCCC

AACACGGTGGCCCTCGTTCGCCCCGTCGACTCCCTTCCTGTGCCTCC

>*SfCAO*_2_11_partial_5prime

tggattgctgtgacacgttgtggtggaattagaaagttctctcacgcttt

ataATGGCATCAGTTTCACAAAAGGTGGCGYCACCTTCTCCTTGCTGTTC

CCCCGGCGGCGACTCTAATCACATTCCACTCCATGCTGCCGCCACTTCCT

CTGCCGAGACTCAAGACTGGACTGATACCATCTCTGACGACCGCCGCCCC

AACACGGTGGCCCTCGTTCKCCCCGTCGACTCCCTTCCTGTGCCTCC

>*SfCAO*_3_5_partial_5prime

tggattgctgtgacacgttgtggtggaattagaaagttctctcacgcttt

ataATGGCATCAGTTTCACAAAAGGTGGCGYCACCTTCTCCTTGCTGTTC

CCCCGGCGGCGACTCTAATCACATTCCACTCCATGCTGCCGCCACTTCCT

CTGCCGAGACTCAAGACTGGACTGATACCATCTCTGACGACCGCCGCCCC

AACACGGTGGCCCTCGTTCKCCCCGTCGACTCCCTTCCTGTGCCTCC

>*SfCAO*_4_15_partial_5prime

tggattgctgtgacacgttgtggtggaattagaaagttctctcacgcttt

ataATGGCATCAGTTTCACAAAAGGTGGCGYCACCTTCTCCTTGCTGTTC

CCCCGGCGGCGACTCTAATCACATTCCACTCCATGCTGCCGCCACTTCCT

CTGCCGAGACTCAAGACTGGACTGATACCATCTCTGACGACCGCCGCCCC

AACACGGTGGCCCTCGTTCKCCCCGTCGACTCCCTTCCTGTGCCTCC

>*SfCAO*_5_13_partial_5prime

tggattgctgtgacacgttgtggtggaattagaaagttctctcacgcttt

ataATGGCATCAGTTTCACAAAAGGTGGCGYCACCTTCTCCTTGCTGTTC

CCCCGGCGGCGACTCTAATCACATTCCACTCCATGCTGCCGCCACTTCCT

CTGCCGAGACTCAAGACTGGACTGATACCATCTCTGACGACCGCCGCCCC

AACACGGTGGCCCTCGTTCKCCCCGTCGACTCCCTTCCTGTGCCTCC

>*SfCAO*_6_16_partial_5prime

tggattgctgtgacacgttgtggtggaattagaaagttctctcacgcttt

ataATGGCATCAGTTTCACAAAAGGTGGCGYCACCTTCTCCTTGCTGTTC

CCCCGGCGGCGACTCTAATCACATTCCACTCCATGCTGCCGCCACTTCCT

CTGCCGAGACTCAAGACTGGACTGATACCATCTCTGACGACCGCCGCCCC

AACACGGTGGCCCTCGTTCKCCCCGTCGACTCCCTTCCTGTGCCTCC

>*SfCAO*_7_10_partial_5prime

tggattgctgtgacacgttgtggtggaattagaaagttctctcacgcttt

ataATGGCATCAGTTTCACAAAAGGTGGCGYCACCTTCTCCTTGCTGTTC

CCCCGGCGGCGACTCTAATCACATTCCACTCCATGCTGCCGCCACTTCCT

CTGCCGAGACTCAAGACTGGACTGATACCATCTCTGACGACCGCCGCCCC

AACACGGTGGCCCTCGTTCKCCCCGTCGACTCCCTTCCTGTGCCTCC

>*SfCAO*_8_11_partial_5prime

tggattgctgtgacacgttgtggtggaattagaaagttctctcacgcttt

ataATGGCATCAGTTTCACAAAAGGTGGCGYCACCTTCTCCTTGCTGTTC

CCCCGGCGGCGACTCTAATCACATTCCACTCCATGCTGCCGCCACTTCCT

CTGCCGAGACTCAAGACTGGACTGATACCATCTCTGACGACCGCCGCCCC

AACACGGTGGCCCTCGTTCKCCCCGTCGACTCCCTTCCTGTGCCTCC

>*SfCAO*_9_8_partial_5prime

tggattgctgtgacacgttgtggtggaattagaaagttctctcacgcttt

ataATGGCATCAGTTTCACAAAAGGTGGCGYCACCTTCTCCTTGCTGTTC

CCCCGGCGGCGACTCTAATCACATTCCACTCCATGCTGCCGCCACTTCCT

CTGCCGAGACTCAAGACTGGACTGATACCATCTCTGACGACCGCCGCCCC

AACACGGTGGCCCTCGTTCKCCCCGTCGACTCCCTTCCTGTGCCTCC

>*SfCAO*_10_4_partial_5prime

tggattgctgtgacacgttgtggtggaattagaaagttctctcacgcttt

ataATGGCATCAGTTTCACAAAAGGTGGCGYCACCTTCTCCTTGCTGTTC

CCCCGGCGGCGACTCTAATCACATTCCACTCCATGCTGCCGCCACTTCCT

CTGCCGAGACTCAAGACTGGACTGATACCATCTCTGACGACCGCCGCCCC

AACACGGTGGCCCTCGTTCKCCCCGTCGACTCCCTTCCTGTGCCTCC

**(D)** *SfCAO1* 3' gDNA sequence

>*SfCAO1*_1_12_partial_3prime_685nt_RefSeq

AACCGGTCTTTAGAAGAGACTAATATAGTTCTTTGgtgcgctctctctctctcttaaaaaagagtgtactgctaatatttttctctataaaatagtttaaaacactgtccaacagtaccaagaaataacctcttctgtaatttatattacattatattaagattagagatgacataaattccccaattccaactttttgttccaatccactttaatgttccacccttaaaatggtttcacattttgtcttctggagactaaccgtgttatcttcagGTATGTATTTGGAGTCACACATGTTCCTCGTTTAGAAGACTGGCCTGTTATGCCAGTAGAGCACATTGGTTTTATGCTCATGgtaattacctgattgattgttttcatattttttttaccactctttttatgctcatggtaattacctgattgattgttttcatatttattaaacttcatctggagattttccaattttctgatcatgaagctctgttgatgtgtgcagCCTCATGGATTCTTCAATTGTTCCCCTGCGATAGATGTGCCACCTAATCCATGTGAATTGGATTCTAAAGATAATGACATCAAGGACAATGGTGCTTTGAAGCCAATTCAGAGTGCGTTAGCGGCAAAGCTTTAGgaacctttcgcaccaaaagttatggcaatgtgctgccgagagaaa

>SfCAO1_2_12_partial_3prime

ACCGGTCTTTAGAAGAGACTAATATAGTTCTTTGgtgcgctctctctctctct

taaaaaagagtgtgttgctartatttttctctataaaatagtttaaaacact

gtccaacagtaccaagaaataacctcttctgtaatttatattacattatatta

agattagagatgacataaattcyccaattccaactttttgttccaatccactt

taatgttccacccttaaaatggtttcacattttgtcttctggagactaaccgt

gttatcttcagGTATGTATTTGGAGTCACACATNNNNNNNNNNNNNNNNNNNN

NNNNNNNNNGCCAGTAGAGCACATTGGTTTTATGCTCATGgtaattacctgat

tgattgttttcatattttttttaccactctttttatgctcatggtaattacct

gattgattgttttcatatttattaaacttcatcyggagrttttccaattktct

gatcatgaagctctgttgatgtgtgcagCCTCATGGATTCTTCAATTGTTCCC

CTGCGATAGATGTGCCACCTAATCCATGTGAATTGGATTCTAAAGATAATGAC

ATCAAGGACAATGGTGCTTTGAAGCCAATTCAGAGTGCGTTAGCGGCAAAGCT

TTAGgaacctttcgcaccaaaagttatggcaatgtgctgccgagaga

>SfCAO1_3_5_partial_3prime

GGTCTTTAGAAGAGACTAATATAGTTCTTTGgtgcgctctctctctctctcttaaaaaaga

nnnnnnnnnnnnnnnnnnnnnnnnnnnnnnnnnnnnnnnnnnnnnnnnnnnnnnnnnnnnn

nnnnnnnnnnnnnnnnnnnnnnnnnnnnnnnnnnnnnnnnnnnnnnn

acataaattccccaattccaactttttgttccaatccactttaatgttccacccttaaaatggtttcacattttgtcttctggagactaaccgtgttatcttcagGTATGTATTTGGAGTCACACATGTTCCTCGTTTAGAAGACTGGCCTGTTATGCCAGTAAAGCACATTGGTTTTATGCTCATGgtaattacctgattgattgttttcatattttttttaccactctttttatgctcatggtaattacctgattgattgttttcatatttattaaacttcatctggagattttccaattttctgatcatgaagctctgttgatgtgtgcagCCTCATGGATTCTTCAATTGTTCCCCTGCGATAGATGTGCCACCTAATCCATGTGAATTGGATTCTAAAGATAATGACATCAAGGACAATGGTGCTTTGAAGCCAATTCAGAGTGCGTTAGCGGCAAAGCTTTAGgaacctttcgcaccaaaagttatggcaa

tgtgctgccgagagaaa

>SfCAO1_5_13_partial_3prime

AACCGGTCTTTAGAAGAGACTAATATAGTTCTTTGgtgcgctctctctctctcttaaaaaagagtgtactgctaatatttttctctataaaatagtttaaaacactgtccaacagtaccaagaaataacctcttctgtaatttatattacattatattaagattagagatgacataaattccccaattccaactttttgttccaatccactttaatgttccacccttaaaatggtttcacattttgtcttctggagactaaccgtgttatcttcagGTATGTATTTGGAGTCACACATGTTCCTCGTTTAGAAGACTGGCCTGTTATGCCAGTAGAGCACATTGGTTTTATGCTCATGgtaattacctgattgattgttttcatattttttttaccactctttttatgctcatggtaattacctgattgattgttttcatatttattaaacttcatctggagattttccaattttctgatcatgaagctctgttgatgtgtgcagCCTCATGGATTCTTCAATTGTTCCCCTGCGATAGATGTGCCACCTAATCCATGTGAATTGGATTCTAAAGATAATGACATCAAGGACAATGGTGCTTTGAAGCCAATTCAGAGTGCGTTAGCGGCAAAGCTTTAGgaacctttcgcaccaaaagttatggcaatgtgctgccgagagaaa

>SfCAO1_6_16_partial_3prime

ACCGGTCTTTAGAAGAGACTAATATAGTTCTTTGgtgcgctctctctctctct

taaaaaagagtgtgytgctartatttttctctataaaatagtttaaaacactgtccaacagtaccaagaaataacctcttctktaatttatattacattatattaagattagagatgacataaattccccaattccaactttttgttccaatccactttaatgttccacccttaaaatggtttcacattttgtcttctggagactaaccgtgttatcttcagGTATGTATTTGGAGTCACACAT

NNNNNNNNNNNNNNNN

ACTGGCCTGTTATGCCAGTAGAGCACATTGGTTTTATGCTCATGgtaattacctgattgattgttttcatatttttttt

accactctttttatgctcatggtaattacctgattgattgttttcatatttattaaacttcatcyggagrttttccaattttctgatcatgaagctctgttgatgtgtgcagCCTCATGGATTCTTCAATTGTTCCCCTGCGATAGATGTGCCACCTAATCCATGTGAATTGGATTCTAAAGATAATGACATCAAGGACAATGGTGCTTTGAAGCCAATTCAGAGTGCGTTAGCGGCAAAGCTTTAGgaacctttcgcaccaaaagttatggcaatgtgctgccgagagaaa

>SfCAO1_7_10_partial_3prime

ACCGGTCTTTAGAAGAGACTAATATAGTTCTTTGgtgcgctctctctctct

cttaaaaaagagtgtgytgctartatttttctctataaaatagtttaaaacactgtccaacagtaccaagaaataacctcttctgtaatttatattacattatattaagattagagatgacataaattcyccaattccaactttttgttccaatccactttaatgttccacccttaaaatggtttcacattttgtcttctggagactaaccgtgttatcttcagGTATGTATTTGGAGTCACACATGTTCCNNNNNNNNNN

GACTGGCCTGTTATGCCAGTAGAGCACATTGGTTTTATGCTCATGgtaattacctgattgattgttttcatattttttttt

accactctttttatgctcatggtaattacctgattgattgttttcatatttattaaacttcatcyggagrttttccaattktctgatcatgaagctctgttgatgtgtgcagCCTCATGGATTCTTCAATTGTTCCCCTGCGATAGATGTRCCACCTAATCCATGTGAATTGGATTCTAAAGATAATGACATCAAGGACAATGGTGCTTTGAAGCCAATTCAGAGTGCGTTAGCGGCAAAGCTTTAGgaacctttcgcaccaaaagttatggcaatgtgctgccgagagaa

>SfCAO1_8_11_partial_3prime

AACCGGTCTTTAGAAGAGACTAATATAGTTCTTTGgtgcgctctctctctcttaaaaaagtgtgtgttgctagtatttttctctataaaatagtttaaaacactgtccnacagtaccaagaaataacctcttctttaatttatattacattatattaagattagagatgacataaattccccaattccaactttttgttccaatccactttaatgttccacccttaaaatggtttcacattttgtcttctggagactaaccgtgttatcttcagGTATGTATTTGGAGTCACACATGTTCCTCGTTTAGAAGACTGGCCCGTTATGCCAGTAGAGCACATTGGTTTTATGCTCATGgtaattacctgattgattgttttcatatttttttttaccactctttttatgctcatggtaattacctgattgattgttttcatatttattaaacttcatccggaggttttccaattttctgatcatgaagctctgttgatgtgtgcagCCTCATGGATTCTTCAATTGTTCCCCTGCGATAGATGTGCCACCTAATCCATGTGAATTGGATTCTAAAGATAATGACATCAAGGACAATGGTGCTTTGAAGCCAATTCAGAGTGCGTTAGCGGCAAAGCTTTAGgaacctttcgcaccaaaagttatggcaatgtgctgccgagagaaa

>SfCAO1_9_8_partial_3prime

AACCGGTCTTTAGAAGAGACTAATATAGTTCTTTGgtgcgctctctctctcttaaaaaagtgtgtgttgctagtatttttctctataaaatagtttaaaacactgtccaacagtaccaagaaataacctcttctttaatttatattacattatattaagattagagatgacataaattccccaattccaactttttgttccaatccactttaatgttccacccttaaaatggtttcacattttgtcttctggagactaaccgtgttat

cttcatccggaggttttccaattttctgatcatgaagctctgttgatgtgtgcagCCTCATGGATTCTTCAATTGTTCCCCTGCGATAGATGTGCCACCTAATCCATGTGAATTGGATTCTAAAGATAATGACATCAAGGACAATGGTGCTTTGAAGCCAATTCAGAGTGCGTTAGCGGCAAAGCTTTAGgaacctttcgcaccaaaagttatggcaatgtgctgccgagagaaa

>SfCAO1_10_4_partial_3prime

ACCGGTCTTTAGAAGAGACTAATATAGTTCTTTGgtgcgctctctctctctcttaaaaaag

agtgtgttgctartatttttctctataaaatagtttaaaacactgtccaacagtaccaagaaataacctcttctgtaatttatattacattatattaagattagagatgacataaattccccaattccaactttttgttccaatccactttaatgttccacccttaaaatggtttcacattttgtcttctggagactaaccgtgttatcttcagGTATGTATTTGGAGTCACACATGTTC

NNNNNNNNNNNGACTGGCCTGTTATGCCAGTAGAGCACATTGGTTTTATGCTCATGgtaattacctgattgattgttttcata

tttttttttaccactctttttatgctcatggtaattacmtgattgattgttttcatatttattaaacttcatcyggagrttttccaattttctgatcatgaagctctgttgatgtgtgcagCCTCATGGATTCTTCAATTGTTCCCCTGCGATAGATGTGCCACCTAATCCATGTGAATTGGATTCTAAAGATAATGACATCAAGGACAATGGTGCTTTGAAGCCAATTCAGAGTGCGTTAGCGGCAAAGCTTTAGgaacctttcgcaccaaaagttatggcaatgtgctgccgagagaaa

**(E)** *SfCAO2* 3' gDNA sequence

>SfCAO2_1_12_partial_3prime_1126nt_RefSeq

AACCGGTCTTTAGAAGAGACTAATATAGTTCTTTGgtgcgctctctctct

ctctctcttaaaaagagtatgttgctagtatttctctctgtaaaatagtt

taaaacactgtccaacagtaccaagaaataacctcttctgtaatttatat

tacattatattaagattagagatgacataaattccccaattccatctttt

tgttccaatccacacataaattccccaattccaattttttgttccaattc

actttaatgttccacccttaaaatggtttcacattttgtcttctggagac

taaccgtgttgtcttcagGTATGTATTTGGAGTCACACATGTTCCTCGTT

TAGAAGACTGGCCTGTTATGCCAGTAGAGCACATTGGTTTTATGCTCATG

gtaattacctgattgattgttttcatattttttttaccactcttttgatt

atagtgtgttgtttgcctttcatgtaagttgcttgatatgatgctcttga

ttttttaatttttcaatgttattatagaacacttggtggtaagtgcttaa

aacatcactagaatattaagaatgttgatttggggtagttgttattaggt

ccggtccaaaggtaaggttagacacacccttacttaggccctcaacccac

cgcactcagtcccctatatagttcactaacaaaaattgcacatgtgagga

tcccttccatgatttattcgctctaaggcgtcacttaattagctttatgc

ctcacattgccttgtgccgaccctggttgttatcggggacaatgtttagt

gtagcacatcttttccatcaccgatgagcttttgttgtctctgttatttt

tttgggggggccttattcttttgtttatgttatttattaaacttcatctg

gaggttttccaattttctgatcatgaagctctgttgatgtgtgcagCCTC

ATGGATTCTTCAATTGTTCCCCTGCGATAGATGTGCCACCTAATCCATGT

GAATTGGATTCTAAAGATAATGACATCAAGGACAATGGTGCTTTGAAGCC

AATTCAGAGTGCGTTAGCGGCAAAGCTTTAGgaacctttcgcaccaaaag

ttatggcaatgtgctgccgagagaaa

>SfCAO2_2_12_partial_3prime

AACCGGTCTTTAGAAGAGACTAATATAGTTCTTTGgtgcgctctctctctctctcttaaaaagagtatgttgctagtatttctctctgtaaaatagtttaaaacactgtccaacagtaccaagaaataacctcttctgtaatttatattacattatattaagattagagatgacataaattccccaattccatctttttgttccaatccacacataaattccccaattccaattttttgttccaattcactttaatgttccacccttaaaatggtttcacattttgtcttctggagactaaccgtgttgtcttcagGTATGTATTTGGAGTCACACATGTTCCTCGTTTAGAAGACTGGCCTGTTATGCCAGTAGAGCACATTGGTTTTATGCTCATGgtaattacctgattgattgttttcatattttttttaccactcttttgattatagtgtgttgtttgcctttcatgtaagttgcttgatatgatgctcttgattttttaatttttcaatgttattatagaacacttggtggtaagtgcttaaaacatcactagaatattaagaatgttgatttggggtagttgttattaggtccggtccaaaggtaaggttagacacacccttacttaggccctcaacccaccgcactcagtcccctatatagttcactaacaaaaattgcacatgtgaggatcccttccatgatttattcgctctaaggcgtcacttaattagctttatgcctcacattgccttgtgccgaccctggttgttatcggggacaatgtttagtatagcacatcttttccatcaccgatgagcttttgttgtctctgttatttttttgggggggccttattcttttgtttatgttatttattaaacttcatctggaggttttccaattttctgatcatgaagctctgttgatgtgtgcagCCTCATGGATTCTTCAATTGTTCCCCTGCGATAGATGTGCCACCTAATCCATGTGAATTGGATTCTAAAGATAATGACATCAAGGACAATGGTGCTTTGAAGCCAATTCAGAGTGCGTTAGCGGCAAAGCTTTAGgaacctttcgcaccaaaagttatggcaatgtgctgccgagagaaa

>SfCAO2_3_5_partial_3prime

AACCGGTCTTTAGAAGAGACTAATATAGTTCTTTGgtgcgctctctctct

ctctcttaaaaagagtatgttgctagtatttctctctgtaaaatagtt

taaaacactgtccaacagtaccaagaaataacctcttctgtaatttatat

tacattatattaagattagagatgacataaattccccaattccatctttt

tgttccaatccacacataaattccccaattccaattttttgttccaattc

actttaatgttccacccttaaaatggtttcacattttgtcttctggagac

taaccgtgttgtcttcagGTATGTATTTGGAGTCACACATGTTCCTCGTT

TAGAAGACTGGCCTGTTATGCCAGTAGAGCACATTGGTTTTATGCTCATG

gtaattacctgattgattgttttcatattttttttaccactcttttgatt

atagtgtgttgtttgcctttcatgtaagttgcttgatatgatgctcttga

ttttttaatttttcaatgttattatagaacacttggtggtaagtgcttaa

aacatcactagaatattaagaatgttgatttggggtagttgttattaggt

ccggtccaaaggtaaggttagacacacccttacttaggccctcaacccac

cgcactcagtcccctatatagttcactaacaaaaattgcacatgtgagga

tcccttccatgatttattcgctctaaggcgtcacttaattagctttatgc

ctcacattgccttgtgccgaccctggttgttatcggggacaatgtttagt

gtagcacatcttttccatcaccgatgagcttttgttgtctctgttatttt

tttgggggggccttattcttttgtttatgttatttattaaacttcatctg

gaggttttycaattttctgatcatgaagctctgttgatgtgtgcagCCTC

ATGGATTCTTCAATTGTTCCCCTGCGATAGATGTGCCACCTAATCCATGT

GAATTGGATTCTAAAGATAATGACATCAAGGACAATGGTGCTTTGAAGCC

AATTCAGAGTGCGTTAGCGGCAAAGCTTTAGgaacctttcgcaccaaaag

ttatggcaatgtgctgccgagagaaa

>SfCAO2_4_15_partial_3prime

ACCGGTCTTTAGAAGAGACTAATATAGTTCTTTGgtgcgctctctctctctctct

taaaaagagtrtgttgctagyatttctctctgtaaaatagtttaaaacactgtccaacagtaccaagaaataacctcttctgtaatttatattacattatattaagattagagatgacataaattccccaattccatctttttgttccaatccacacataaattccccaattccaaytttttgttccaattcactttaatgttccacccttaaaatggtttcacattttgtcttctggagactaaccgtgttgtcttcagGTATGTATTTGGAGTCACACANNNNNNNNNNNNNN

AAGACTGGCCTGTTATGCCAGTAGAGCACATTGGTTTTATGCTCATGgtaattacctgattgattgttttcatattttttttaccactcttttgattatagtgtgnnnnnnnnnnnnnnnnnnnnnnnnnnnnnnnnnnnnnnnnnnnnnnnnnnnnnnnnnnnnnnnnnnnnnnnnnnnnnnnnnnnnnnnnnnnnnnnnnnnnnnnnnnnnnnnnnnnnnnnnnnnnnnnnnnnnnnnnnnnnnnnnnnnnnnnnnnnnnnnnnnnnnnnnnnnnnnnnnnnnnnnnnnnnnnnnnnnnnnnnnnnnnnnnnnnnnnnnnnnnnnnnnnnnnnnnnnnnnnnnnnnnnnnnnnnnnnnnnnnnnnnnnnnnnnnnnn

agtyaattagctttatgcctcacattgccttgtgccgaccctggttgttatcggggacaatgtttagtatagcacatcttttccatcaccaatgagcttttgttgtctctgttatttttttgggggggccttattcttttgtttatgttatttattaaacttcatctggaggttttycaattttctgatcatgaagctctgttgatgtgtgcagCCTCATGGATTCTTCAATTGTTCCCCTGCGATAGATGTGCCACCTAATCCATGTGAATTGGATTCTAAAGATAATGACATCAAGGACAATGGTGCTTTGAAGCCAATTCAGAGTGCGTTAGCGGCAAAGCTTTAGgaacctttcgcaccaaaagttatggcaatgtgctgccgagagaaa

>SfCAO2_5_13_partial_3prime

AACCGGTCTTTAGAAGAGACTAATATAGTTCTTTGgtgcgctctctctctctctcttaaaaagagtrtgttgctagyatttctctctgtaaaatagtttaaaacactgtccaacagtaccaagaaataacctcttctgtaatttatattacattatattaagattagagatgacataaattccccaattccatctttttgttccaatccacacataaattccccaattccaaytttttgttccaattcactttaatgttccacccttaaaatggtttcacattttgtcttctggagactaaccgtgttgtcttcagGTATGTATTTGGAGTCACACATGTTCCTCGTTTAGAAGACTGGCCTGTTATGCCAGTAGAGCACATTGGTTTTATGCTCATGgtaattacctgattgattgttttcatattttttttaccactcttttgattatagtgtgttgt

nnnnnnnnnnnnnnnnnnnnnnnnnnnnnnnnnnnnnnnnnnnnnnnnnnnnnnnnnnnnnnnnnnnnnnnnnnnnnnnnnnnnnnnnnnnnn

nnnnnnnnnnnnnnnnnnnnnnnnnnnnnnnnnnnnnnnnnnnnnnnnnnnnnnnnnnnnnnnnnnnnnnnnnnnnnnnnnnnnnnnnnnnnnnnn

nnnnnnnnnnnnnnnnnnnnnnnnnnnnnnnnnnnnnnnnnnnnnnnnnnnnnnnnnnnnnnnnnnnnnnnnnnnnnnnnnn

gtyaattagctttatgcctcacattgccttgtgccgaccctggttgttatcggggacaatgtttagtatagcacatcttttccatcaccratgagcttttgttgtctctgttatttttttgggggggccttattcttttgtttatgttatttattaaacttcatctggaggttttycaattttctgatcatgaagctctgttgatgtgtgcagCCTCATGGATTCTTCAATTGTTCCCCTGCGATAGATGTGCCACCTAATCCATGTGAATTGGATTCTAAAGATAATGACATCAAGGACAATGGTGCTTTGAAGCCAATTCAGAGTGCGTTAGCGGCAAAGCTTTAGgaacctttcgcaccaaaagttatggcaatgtgctgccgagagaaa

>SfCAO2_6_16_partial_3prime

AACCGGTCTTTAGAAGAGACTAATATAGTTCTTTGgtgcgctctctctctctctc

tcttaaaaagagtatgttgctagtatttctctctgtaaaatagtttaaaacactgtccaacagtaccaagaaataacctcttctgtaatttatattacattatattaagattagagatgacataaattccccaattccatctttttgttccaatccacacataaattccccaattccaattttttgttccaattcactttaatgttccacccttaaaatggtttcacattttgtcttctggagactaaccgtgttgtcttcagGTATGTATTTGGAGTCACACAT

GTTCCTCGTTTAGAAGACTGGCCTGTTATGCCAGTAGAGCACATTGGTTTTATGCTCATGgtaattacctgattgattgttttcatattttttttaccactcttttgattatagtgtgttgtttgcctttcatgtaagttgcttgatatgatgctcttgattttttaatttttcaatgttattatagaacacttggtggtaagtgcttaaaacatcactagaatattaagaatgttgatttggggtagttgttattaggtccggtccaaaggtaaggttagacacacccttacttaggccctcaacccaccgcactcagtcccttatatagttcactaacaaaaattgcacatgtgaggatcccttccatgatttattcgctctaaggcgtcacttaattagctttatgcctcacattgccttgtgccgaccctggttgttatcggggacaatgtttagtrtagcacatcttttccatcaccaatgagcttttgttgtctctgttatttttttgggggggccttattcttttgtttatgttatttattaaacttcatctggaggttttccaattttctgatcatgaagctctgttgatgtgtgcagCCTCATGGATTCTTCAATTGTTCCCCTGCGATAGATGTGCCACCTAATCCATGTGAATTGGATTCTAAAGATAATGACATCAAGGACAATGGTGCTTTGAAGCCAATTCAGAGTGCGTTAGCGGCAAAGCTTTAGgaacctttcgcaccaaaagttatggcaatgtgctgccgagagaaa

>SfCAO2_7_10_partial_3prime

AACCGGTCTTTAGAAGAGACTAATATAGTTCTTTGgtgcgctctctctctctctcttaaaaagagtatgttgctagtatttctctctgtaaaatagtttaaaacactgtccaacagtaccaagaaataacctcttctgtaatttatattacattatattaagattagagatgacataaattccccaattccatctttttgttccaatccacacataaattccccaattccaattttttgttccaattcactttaatgttccacccttaaaatggtttcacattttgtcttctggagactaaccgtgttgtcttcagGTATGTATTTGGAGTCACACATGTTCCTCGTTTAGAAGACTGGCCTGTTATGCCAGTAGAGCACATTGGTTTTATGCTCATGgtaattacctgattgattgttttcatattttttttaccactcttttgattatagtgtgttgtttgcctttcatgtaagttgcttgatatgatgctcttgattttttaatttttcaatgttattatagaacacttggtggtaagtgcttaaaacatcactagaatattaagaatgttgatttggggtagttgttattaggtccggtccaaaggtaaggttagacacacccttacttaggccctcaacccaccgcactcagtcccctatatagttcactaacaaaaattgcacatgtgaggatcccttccatgatttattcgctctaaggcgtcacttaattagctttatgcctcacattgccttgtgccgaccctggttgttatcggggacaatgtttagtrtagcacatcttttccatcaccgatgagcttttgttgtctctgttatttttttgggggggccttattcttttgtttatgttatttattaaacttcatctggaggttttccaattttctgatcatgaagctctgttgatgtgtgcagCCTCATGGATTCTTCAATTGTTCCCCTGCGATAGATGTGCCACCTAATCCATGTGAATTGGATTCTAAAGATAATGACATCAAGGACAATGGTGCTTTGAAGCCAATTCAGAGTGCGTTAGCGGCAAAGCTTTAGgaacctttcgcaccaaaagttatggcaatgtgctgccgagagaaa

>SfCAO2_8_11_partial_3prime

AACCGGTCTTTAGAAGAGACTAATATAGTTCTTTGgtgcgctctctctctctcttaaaaagag

trtgttgctagyatttctctctgtaaaatagtttaaaacactgtccaacagtaccaagaaataacctcttctgtaatttatattacattatattaagattagagatgacataaattccccaattccatctttttgttccaatccacacataaattccccaattccaaytttttgttccaattcactttaatgttccacccttaaaatggtttcacattttgtcttctggagactaaccgtgttgtcttcagGTATGTATTTGGAGTCACACAT

NNNNNNNNNNNNNN

AGACTGGCCTGTTATGCCAGTAGAGCACATTGGTTTTATGCTCATGgtaattacctgattgattgttttcatattttttttaccactcttttgattatagtgtgttgt

nnnnnnnnnnnnnnnnnnnnnnnnnnnnnnnnnnnnnnnnnnnnnnnnnnnnnnnnnnnnnnnnnnnnnnnnnnnnnnnnnnnnnnnnnnnnn

nnnnnnnnnnnnnnnnnnnnnnnnnnnnnnnnnnnnnnnnnnnnnnnnnnnnnnnnnnnnnnnnnnnnnnnnnnnnnnnnnnnnnnnnnnnnnnnn

nnnnnnnnnnnnnnnnnnnnnnnnnnnnnnnnnnnnnnnnnnnnnnnnnnnnnnnnnnnnnnnnnnnnnnnnnnnnnnnnnn

gtyaattagctttatgcctcacattgccttgtgccgaccctggttgttatcggggacaatgtttagtatagcacatcttttccatcaccaatgagcttttgttgtctctgttatttttttgggggggccttattcttttgtttatgttatttattaaacttcatctggaggttttycaattttctgatcatgaagctctgttgatgtgtgcagCCTCATGGATTCTTCAATTGTTCCCCTGCGATAGATGTGCCACCTAATCCATGTGAATTGGATTCTAAAGATAATGACATCAAGGACAATGGTGCTTTGAAGCCAATTCAGAGTGCGTTAGCGGCAAAGCTTTAGgaacctttcgcaccaaaagttatggcaatgtgctgccgagagaaa

>SfCAO2_9_8_partial_3prime

AACCGGTCTTTAGAAGAGACTAATATAGTTCTTTGgtgcgctctctctctctctcttaaaaagagtatgttgctagtatttctctctgtaaaatagtttaaaacactgtccaacagtaccaagaaataacctcttctgtaatttatattacattatattaagattagagatgacataaattccccaattccatctttttgttccaatcaacacataaattccccaattccaattttttgttccaattcactttaatgttccacccttaaaatggtttcacattttgtcttctggagactaaccgtgttgtcttcagGTATGTATTTGGAGTCACACATGTTCCTCGTTTAGAAGACTGGCCTGTTATGCCAGTAGAGCACATTGGTTTTATGCTCATGgtaattacctgattgattgttttcatatttttttttaccactcttttgattatagtgtgttgtttgcctttcatgtaagttgcttgatatgatgctcttgattttttaatttttcaatgttattatagaacacttggtggtaagtgcttaaaacatcactagaatattaagaatgttgatttggggtagttgttattaggtccggtccaaaggtaaggttagacacacccttacttaggccctcaacccaccgcactcagtcccctatatagttcactaacaaaaattgcacatgtgaggatcccttccatgatttattcgctctaaggcgtcacttaattagctttatgcctcacattgccttgtgccgaccctggttgttatcggggacaatgtttagtatagcacatcttttccatcaccaatgagcttttgttgtctctgttatttttttgggggggccttattcttttgtttatgttatttattaaacttcatctggaggttttccaattttctgatcatgaagctctgttgatgtgtgcagCCTCATGGATTCTTCAATTGTTCCCCTGCGATAGATGTGCCACCTAATCCATGTGAATTGGATTCTAAAGATAATGACATCAAGGACAATGGTGCTTTGAAGCCAATTCAGAGTGCGTTAGCGGCAAAGCTTTAGgaacctttcgcaccaaaagttatggcaatgtgctgccgagagaaa

>SfCAO2_10_4_partial_3prime

ACCGGTCTTTAGAAGAGACTAATATAGTTCTTTGgtgcgctctctctctctctct

nnnnnnnnnnnnnnnnnnnnnnnnnnnnnnnnnnnnnnnnnnnnnnnnnnnnnnnnnnnnnnnnnnnnnnnnnnnnnnnnnnnnnnnnnnnnnnnnnnnnnnnnnnnnnnnnnnnnnnnnnnnn

nnnnnnnnnnnnnnnnnnnnnnnnnnnnnacacataaattccccaattccaaytttttgttccaattcactttaatgttccacccttaaaatggtttcacattttgtcttctggagactaaccgtgttgtcttcagGTATGTATTTGGAGTCACACATGTTCCTNNNNNNNAAGACTGGCCTGTTATGCCAGTAGAGCACATTGGTTTTATGCTCATGgtaattacctgattgattgttttcatattttttttaccactcttttnnnnnnnnnnnnnnnnnnnnnnnnnnnnnnnnnnnnnnnnnnnnnnnnnnnnnnnnnnnnnnnnnnnnnnnnnnnnnnnnnnnnnnnnnnnnnnnnnnnnnnnnnnnnnnnnnnnnnnnnnnnnnnnnnnnnnnnnnnnnnnnnnnnnnnnnnnnnnnnnnnnnnnnnnnnnnnnnnnnnnnnnnnnnnnnnnnnnnnnnnnnnnnnnnnnnnnnnnnnnnnnnnnnnnnnnnnnnnnnnnnnnnnnnnnnnnnnnnnnnnnnnnnnnnnnnnnnnnnnnnnnnnnn

ctyaattagctttatgcctcacattgccttgtgccgaccctggttgttatcggggacaatgtttagtatagcacatcttttccatcaccaatgagcttttgttgtctctgttatttttttgggggggccttattcttttgtttatgttatttattaaacttcatctggaggttttccaattttctgatcatgaagctctgttgatgtgtgcagCCTCATGGATTCTTCAATTGTTCCCCTGCGATAGATGTGCCACCTAATCCATGTGAATTGGATTCTAAAGATAATGACATCAAGGACAATGGTGCTTTGAAGCCAATTCAGAGTGCGTTAGCGGCAAAGCTTTAGgaacctttcgcaccaaaagttatggcaatgtgctgccgagagaaa

**(F)** *SfCAO* fulll length cDNAs from two samples

>SfCAO2_1_LC1_cDNA_2441nt

tggattgctgtgacacgttgtggtggaattagaaagttctctcacgcttt

ataATGGCATCAGTTTCACAAAAGGTGGCGCCACCTTCTCCTTGCTGTTC

CCCCGGCGGCGACTCTAATCACATTCCACTCCATGCTGCCGCCACTTCCT

CTGCCGAGACTCAAGACTGGACTGATACCATCTCTGACGACCGCCGCCCC

AACACGGTGGCCCTCGTTCGCCCCGTCGACTCCCTTCCTGTGCCTCCCAC

CAATGCTCCCACTGTCAAAGGAATCACTACAATGCCAAGGCCCCAGTCAA

GCCACCCTTTGGACCCTTTATCTGCTGCTGAAATCTCTGTGGCAGTGGCA

ACTGTGAGGGCTGCTGGTTCCACTCCTGAGCTGAGAGACAGTATGCGCTT

CCTTGAAGTAGTTTTGGTGGAACCAGATAAACATGTTATTGCACTTGCAG

ATGCTTATTTCTTCCCACCATTCCAACCATCATTACTTCATAGAACTAAA

GGAGGGCCTCTCATTCCAACTAAACTCCCTCCAAGATGTGCTAAACTTGT

TGTTTACAGTAGGAAGACAAATGAGACTACTATATGGATTGTTGAATTAT

CACAAGTACATGCAGTAACTAGAGGTGGTCATCATAGAGGAAAAGTAATT

GTATCACATGTTATTCCTGATGTTCAGCCTCCAATGGATGCTGTGGAGTA

TGCAGAATGTGAGGCTGCTGTTAAAAGTTTTCCTCCATTTATAGAGGCTA

TGAAGAAAAGGGGTGTTGAAGACATGGACCTTGTCATGGTTGACCCCTGG

TGTGCAGGTTATTTCAGTGAAGCTGATGCTCCCAAGAGAAGACTTGCTAA

ACCACTAATATTTTGTCGATCCGAGAGTGACTGCCCTATGGAAAATGGCT

ATGCACGCCCCGTTGAGGGTATCTTTGTTCTTGTTGATATGCAAAACATG

GTGGTGATAGAGTTTGAAGACCGCAAGCTTGTTCCTCTGCCTCCAGTTGA

TCCATTAAGGAACTATACTCGCGGTGAAACAAGAGGTGGCACTGATAGAA

GTGATGTAAAACCTTTGCAAATTATTCAACCCGAAGGTCCAAGCTTTCGT

GTCAATGGATATTATGTTGAATGGCAGAAGTGGAATTTTCGTGTTGGATT

CACACCCAAAGAAGGTTTGGTTATATATTCTGTTGCGTATGATGATGGTA

GTCGAGGGCGAAGACCCGTAGCTCATAGGCTGAGTTTCGTGGAGATGGTT

GTACCCTATGGAGATCCAAATGATCCACATTACAGGAAAAATGCTTTTGA

TGCTGGGGAAGATGGTTTAGGAAGAAATGCACATTCCCTCAAGAAGGGAT

GTGATTGTTTGGGTTTCATCAAATATTTTGATGCTCACTTTACAAATTTC

ACTGGTGGAGTGGAGACAATTGAGAATTGTGTGTGTATGCATGAAGAAGA

TCATGGAATTCTGTGGAAGCATCAAGATTGGAGAACTGGCTTAGCAGAAG

TCAGAAGGTCTAGAAGACTTACAGTGTCTTTTATATGTACTGTGGCTAAC

TATGAGTATGGATTTTTTTGGCACTTTTATCAGGATGGAAAGATTGAAGC

TGAAGTTAAGCTAACTGGAATTCTCAGCTTAGGAGCCTTGATGCCTGGAG

AGTATCGAAAATATGGAACCATGATTGCCCCAGGTCTGTATGCTCCAGTT

CATCAGCACTTTTTTGTTGCTCGTATGGACATGGCTGTTGATTCTAAACC

TGGTGAAGCTTTGAATCAGGTTGTGGAGGTAAATGTGAAAATTGAGGAAC

CTGGTGAGAATAATGTTCACAATAATGGATTCTATGCTGAAGAAACTTTG

CTCAGATCTGAATTGGAAGCCGTGCGCGATTGCAATCCTATGACTGCTCG

GCATTGGATTGTAAGGAACACAAGATCAAGCAATAGAACTGGAGAGTTAA

CAGGCTACAAGCTAGTACCAGGCTCAAACTGCTTACCATTAGCAGGTTCT

GATGCCAAGTTTTTAAGAAGAGCTGCTTTCTTGAAGCATAATCTTTGGGT

AACAGCTTATTCACCTGATGAGATGTTTCCTGGAGGAGAATTTCCTAATC

AAAATCCACGTATTGGCGAAGGATTACCTACATGGGTTAAGCAGAACCGG

TCTTTAGAAGAGACTAATATAGTTCTTTGGTATGTATTTGGAGTCACACA

TGTTCCTCGTTTAGAAGACTGGCCTGTTATGCCAGTAGAGCACATTGGTT

TTATGCTCATGCCTCATGGATTCTTCAATTGTTCCCCTGCGATAGATGTG

CCACCTAATCCATGTGAATTGGATTCTAAAGATAATGACATCAAGGACAA

TGGTGCTTTGAAGCCAATTCAGAGTGCGTTAGCGGCAAAGCTTTAGgaac

ctttcgcaccaaaagttatggcaatgtgctgccgagagaaa

>SfCAO2_9_AC1_cDNA_2441nt

tggattgctgtgacacgttgtggtggaattagaaagttctctcacgcttt

ataATGGCATCAGTTTCACAAAAGGTGGCGCCACCTTCTCCTTGCTGTTC

CCCCGGCGGCGACTCTAATCACATTCCACTCCATGCTGCCGCCACTTCCT

CTGCCGAGACTCAAGACTGGACTGATACCATCTCTGACGACCGCCGCCCC

AACACGGTGGCCCTCGTTCGCCCCGTCGACTCCCTTCCTGTGCCTCCCAC

CAATGCTCCCACTGTCAAAGGAATCACTACAATGCCAAGGCCCCAGTCAA

GCCACCCTTTGGACCCTTTATCTGCTGCTGAAATCTCTGTGGCAGTGGCA

ACTGTGAGGGCTGCTGGTTCCACTCCTGAGCTGAGAGACAGTATGCGCTT

CCTTGAAGTAGTTTTGGTGGAACCAGATAAACATGTTATTGCACTTGCAG

ATGCTTATTTCTTCCCACCATTCCAACCATCATTACTTCATAGAACTAAA

GGAGGGCCTCTCATTCCAACTAAACTCCCTCCAAGATGTGCTAAACTTGT

TGTTTACAGTAGGAAGACAAATGAGACTACTATATGGATTGTTGAATTAT

CACAAGTACATGCAGTAACTAGAGGTGGTCATCATAGAGGAAAAGTAATT

GTATCACATGTTATTCCTGATGTTCAGCCTCCAATGGATGCTGTGGAGTA

TGCAGAATGTGAGGCTGCTGTTAAAAGTTTTCCTCCATTTATAGAGGCTA

TGAAGAAAAGGGGTGTTGAAGACATGGACCTTGTAATGGTTGATCCCTGG

TGTGCAGGTTATTTCAGTGAAGCTGATGCTCCCAAGAGAAGACTTGCTAA

ACCACTAATATTTTGTCGATCCGAGAGTGACTGCCCTATGGAAAATGGCT

ATGCACGCCCCGTTGAGGGTATCTTTGTTCTTGTTGATATGCAAAACATG

GTGGTGATAGAGTTTGAAGACCGCAAGCTTGTTCCTCTGCCTCCAGTTGA

TCCATTAAGGAACTATACTCGCGGTGAAACAAGAGGTGGCACTGATAGAA

GTGATGTAAAACCTTTGCAAATTATTCAACCCGAAGGTCCAAGCTTTCGT

GTCAATGGATATTATGTTGAATGGCAGAAGTGGAATTTTCGTGTTGGATT

CACACCCAAAGAAGGTTTGGTTATATATTCTGTTGCGTATGATGATGGTA

GTCGAGGGCGAAGACCCGTAGCTCATAGGCTGAGTTTCGTGGAGATGGTT

GTACCCTATGGAGATCCAAATGATCCACATTACAGGAAAAATGCTTTTGA

TGCTGGGGAAGATGGTTTAGGAAGAAATGCACATTCCCTCAAGAAGGGAT

GTGATTGTTTGGGTTTCATCAAATATTTTGATGCTCACTTTACAAATTTC

ACTGGTGGAGTGGAGACAATTGAGAATTGTGTGTGTATGCATGAAGAAGA

TCATGGAATTCTGTGGAAGCATCAAGATTGGAGAACTGGCTTAGCAGAAG

TCAGAAGGTCTAGAAGACTTACAGTGTCTTTTATATGTACTGTGGCTAAC

TATGAGTATGGATTTTTTTGGCACTTTTATCAGGATGGAAAGATTGAAGC

TGAAGTTAAGCTAACTGGAATTCTCAGCTTAGGAGCCTTGATGCCTGGAG

AGTATCGAAAATATGGAACCATGATTGCCCCAGGTCTGTATGCTCCAGTT

CATCAGCACTTTTTTGTTGCTCGGATGGACATGGCTGTTGATTCTAAACC

TGGTGAAGCTTTGAATCAGGTTGTGGAGGTAAATGTGAAAATTGAGGAAC

CTGGTGAGAATAATGTTCACAATAATGCATTCTATGCTGAAGAAACTTTG

CTCAGATCTGAATTGGAAGCCGTGCGCGATTGCAATCCCATGACTGCTCG

GCATTGGATTGTAAGGAACACAAGATCGAGCAATAGAACTGGAGAGTTAA

CAGGCTACAAGCTAGTACCAGGCTCAAACTGCTTACCATTAGCAGGTTCC

GATGCCAAGTTTTTAAGAAGAGCTGCTTTCTTGAAGCATAATCTTTGGGT

AACAGCTTATTCACCTGATGAGATGTTTCCTGGAGGAGAATTTCCTAATC

AAAATCCACGTATTGGCGAAGGATTACCTACATGGGTTAAGCAGAACCGG

TCTTTAGAAGAGACTAATATAGTTCTTTGGTATGTATTTGGAGTCACACA

TGTTCCTCGTTTAGAAGACTGGCCTGTTATGCCAGTAGAGCACATTGGTT

TTATGCTCATGCCTCATGGATTCTTCAATTGTTCCCCTGCGATAGATGTG

CCACCTAATCCATGTGAATTGGATTCTAAAGATAATGACATCAAGGACAA

TGGTGCTTTGAAGCCAATTCAGAGTGCGTTAGCGGCAAAGCTTTAGgaac

ctttcgcaccaaaagttatggcaatgtgctgccgagagaaa
