# Supplementary material for: Analysis of Genetic Diversity in the Traditional Chinese Medicine Plant ‘Kushen’ (Sophora flavescens Ait.)
Source: Front Plant Sci. 2021 Aug 3;12:704201. doi: 10.3389/fpls.2021.704201 (PMC8369264; doi:10.3389/fpls.2021.704201)
Supplement: Supplementary Figure 1 — Plants prior to harvest and photographs of the harvested root pieces. [file Data_Sheet_1.zip › Supplementary Tables and Figures.DOCX]

Supplementary Material

# Supplementary Tables

**TABLE S1 |** Primer names, sequences, T_m_ and their use.

| **Primer name** | **Sequence (5' to 3')** | **Tm (°C) ^a^** | **Use** |
| --- | --- | --- | --- |
| SfLDC_F_5pr | AGGGTCGTCATTTGTGTTCTCC | 66.6 | PCR, sequencing |
| SfLDC_R1 | TGAGTTTCCTAAACCAAAAGATAGACAAG | 65 | PCR, sequencing & qPCR |
| SfLDC_F314 | CCCACAGTTCAACCATTCTACG | 65.7 | PCR, sequencing |
| SfLDC_F752 | ATATCGGAAGTGGAGGAGCTG | 64.7 | PCR, sequencing |
| SfLDC_R596 | GGATGAGAAGCTCACATTTCG | 64.1 | PCR, sequencing |
| SfLDC_R1026 | TCGCCTCTTACACGCTTCC | 65.8 | PCR, sequencing |
| SfCAO_5p_Fd | RAGAGTGAGTYTCCACTGTATGTCAG | 64.2 | PCR, sequencing |
| SfCAO_e1_R | TGACAGTGGGAGCATTGGTG | 67.7 | PCR, sequencing |
| SfCAO_e10_F | GAAGGATTACCTACATGGGTTAAGCAG | 66.5 | PCR, sequencing |
| SfCAO_R1 | AAGTGGATGCCAAAATAGTATTCGTG | 66 | PCR, sequencing & qPCR |
| SfCAO_e11_F | GGAGTCACACATGTTCCTCGTTTAG | 66.9 | PCR, sequencing |
| SfCAO_e11_R | CATAACAGGCCAGTCTTCTAAACG | 64.9 | PCR, sequencing |
| SfCAO_e5_F | TTGTCGATCCGAGAGTGACTG | 65.9 | PCR, sequencing |
| SfCAO_ex8_F | CCAGGTCTGTATGCTCCAGTTC | 65.2 | PCR, sequencing |
| SfCAO_ex7_R | CCAATCTTGATGCTTCCACAG | 64.9 | PCR, sequencing |
| SfCAO_ex4_R | CTCACATTCTGCATACTCCACAG | 64 | PCR, sequencing |
| SfLDC_F1 | TATCGCTACCTACCTTGCATGTTC | 65 | qPCR |
| SfCAO_F1 | GCGATAGATGTGCCACCTAATCC | 67 | qPCR |
| SfTUB_F1 | CGTGCTTTTGTTCACTGGTATGTG | 67 | qPCR |
| SfTUB_R1 | CCGATCTACACACACCATAACCATC | 67 | qPCR |
| SfCYC_F1 | GATCTCAGTTCTTCATCTGCACGAC | 67 | qPCR |
| SfCYC_R1 | ACACCGCATAGACAAGACGACAC | 67 | qPCR |
| SfEFa_F1 | GGACATGCGTCAAACTGTGG | 67 | qPCR |
| SfEFa_R1 | CAAAGGATAAATTACTAACAAGGAACCACTG | 67 | qPCR |
| SfUBQ_F1 | GCAAATGCTACGCTCGTCTACATCC | 70.4 | qPCR |
| SfUBQ_R1 | ACATCAAAAAGAGAGGAACCATAACAGCTAG | 68.6 | qPCR |

^a^ Tm calculated using modified nearest neighbour method, <https://www.thermofisher.com/au/en/home/brands/thermo-scientific/molecular-biology/molecular-biology-learning-center/molecular-biology-resource-library/thermo-scientific-web-tools/multiple-primer-analyzer.html>

**TABLE S2** | Comparison of DArTSeq and SNP genotype results for one *S. flavescens* plant from each of four regions of China.

|  |  | GBS and KASP genotype calls^a^ | | | | | | | | | | |
| --- | --- | --- | --- | --- | --- | --- | --- | --- | --- | --- | --- | --- |
|  |  | Provinces | | | | | | | | | | |
| GBS tag sequence from DArTSeq | KASP assay^b^ | Liaoning | |  | Jilin | |  | Shaanxi | |  | Guizhou | |
|  |  | GBS | KASP |  | GBS | KASP |  | GBS | KASP |  | GBS | KASP |
| TGCAGCAAGGACACGTGTTCCTCTTC[C/A]CTAGCCACGGCAATATGCACATCCAATGAAACAAGTGCTGAC | Sf1 | C:C | C:C |  | C:C | C:C |  | C:C | C:C |  | A:A | A:A |
| TGCAGAGAAGTCACGCGCGAGCAGAA[C/T]AGGGGGAGCAACCAGGACCGCCCGCCGCCGCGCCG | Sf2 | C:C | C:C |  | C:C | C:C |  | C:C | C:C |  | T:T | T:T |
| TGCAGCTGCATGCCTGGTCAGGATA[C/T]TGGTGTTGATGAAATTCCCTTTCTTCACCTTCTTCGGGTTGCA | Sf3 | C:C | C:C |  | C:C | C:C |  | T:T | T:T |  | C:C | C:C |
| TGCAGTTGTTTCTGGTGCCAACACAAT[T/C]GAAATTTTCAAGGTTCATGGTATATGTATCATAGAACCTAG | Sf5 | T:T | C:T |  | T:T | T:T |  | C:C | C:C |  | C:C | C:C |
| TGCAGCATAGTACTGATCATCAAGGAAAGGGAGAA[A/C]TGGAGAGGAGATAAAAGAAATCAATGATTGATT | Sf6 | A:A | A:A |  | A:A | A:A |  | C:C | C:C |  | C:C | A:A |
| TGCAGTTTTTGCTGTGAGTCTAGTGTTGT[T/G]TGTAAGTATATTTGTCCACAAGCTACCGCATTGAAATTT | Sf7 | T:T | G:T |  | G:G | G:G |  | T:T | T:T |  | T:T | T:T |
| TGCAGCTAGATGGGCTCCATCGTCTCATCCA[T/G]TTGATCCTATCCTCGAATAAAATCCCAAGGCAGGCCA | Sf8 | T:T | T:T |  | G:G | G:G |  | T:T | T:T |  | T:T | T:T |
| TGCAGGGAAGTCGAACCCCTTCCTGATAGATGCT[C/T]GAGTGTCTCTTCAACTCTTCCCTAGCCTATCTTC | Sf10 | C:C | C:T |  | T:T | T:T |  | T:T | T:T |  | C:C | C:C |
| TGCAGGAACCTTAACAAACTGAAGCAA[A/G]CTTAAAGCTTCTTCAAAACCCACGTCAATTTCGCCTTTTCT | Sf12 | G:G | G:G |  | A:A | A:A |  | A:A | A:A |  | A:A | A:A |
| TGCAGCGGGTTCAGAATCCGACGGTGGTGG[T/C]TCCGCCACGTGTTTCCCTTTCGAGTCATGTTTATTACC | Sf13 | C:C | C:C |  | T:T | T:T |  | T:T | T:T |  | T:T | T:T |
| TGCAGGCACCCACATGGTATTCTTT[G/A]TCCTGAATATAGTTGAAGGGGTTGGTACACTAATGATGTTCAC | Sf14 | A:A | A:A |  | G:G | G:G |  | G:G | G:G |  | A:A | A:A |
| TGCAGGTGAAACAGATGCTGCTAAATT[G/T]GCAATTGCAACTGCTGAAGAGTCCAGGAAAGCTGTTCAGAA | Sf15 | T:T | G:T |  | G:G | G:G |  | G:G | G:G |  | T:T | T:T |
| TGCAGGATGCTGGGGTTACCATGTC[T/C]CCTGACCTTAGGGCAACTGCTTTATCTAACGTCATCAAGAATG | Sf16 | C:C | C:C |  | T:T | T:T |  | C:C | C:C |  | T:T | T:T |
| TGCAGCACAGTTGCTCGACTGATGATTGG[T/C]TTTTTTGCTTTTGAATTCACTTTCAGGGAACTTCAAATG | Sf17 | C:C | - |  | T:T | C:T |  | C:C | C:C |  | T:T | T:T |
| TGCAGACCAGCAAGCCATAGTTCAAT[A/T]TATTGGCTCTTTTAATTCCTACAAGGCTTTATTGGAAGGAAG | Sf19 | T:T | - |  | A:A | A:A |  | T:T | T:T |  | T:T | T:T |

^a^ Genomic DNA was extracted from leaves of seedlings grown for the field trial site, from seed originally sourced from Liaoning, Jilin, Shaanxi and Guizhou provinces (regions 1, 3, 7 and 9, respectively **Figure 2A**). Shading indicates agreement differences between GBS and KASP markers. - not scored.

**TABLE S3** | Genotypes from DArTseq analysis of 85 plants from nine regions in China

See separate Excel file

**TABLE S4** | Genotype information for single nucleotide polymorphisms (SNPs) identified in the *SfLDC* gene of 10 *S. flavescens* samples.

| **LDC primer** | **Position^a^** | **SNP^b^** | **AA change^c^** | **Genotype of *S. flavescens* individuals**^d^ | | | | | | | | | |
| --- | --- | --- | --- | --- | --- | --- | --- | --- | --- | --- | --- | --- | --- |
|  |  |  |  | **1_12** | **2_11** | **3_5** | **4_15^e^** | **5_13** | **6_16** | **7_10** | **8_11** | **9_8^e^** | **10_4** |
| R1026 | 41 | T/A | in 5’ UTR | TT | AA | TT |  | TT | TT | TA | TT |  | TA |
| R596 | 41 | T/A |  | TT | AA | TT | TT | TT | TT | TA | TT | AA | TA |
| 5pr_F | 41 | T/A |  | TT | AA | TT | TT | TT | TT | TA | TT | AA | TA |
|  |  |  |  |  |  |  |  |  |  |  |  |  |  |
| R1026 | 381 | C/T | No, GCC/GCT, Ala | TT | TT | TT | CC | CC | CC | CT | TT |  | CT |
| R596 | 381 | C/T |  | TT | TT | TT | CC | CC | CC | CC | TT | TT | CT |
| 5pr_F | 381 | C/T |  | TT | TT | TT | CC | CC | CC | CC | TT | TT | CT |
| R1 | 381 | C/T |  | TT | TT | TT |  | CC | CC | CC | TT |  | CT |
| F314 | 381 | C/T |  | TT | TT | TT | CC | CC | CC | CC | TT |  | CT |
|  |  |  |  |  |  |  |  |  |  |  |  |  |  |
| R1026 | 414 | G/T | No, CTG/CTT, Leu | GG | GG | GG | TT | TT | TT | TT | GG |  | GT |
| R596 | 414 | G/T |  | GG | GG | GG | TT | TT | TT | TT | GG | GG | GT |
| 5pr_F | 414 | G/T |  | GG | GG | GG | TT | TT | TT | TT | GG | GG | GT |
| F314 | 414 | G/T |  | GG | GG | GG | TT | TT | TT | TT | GG |  | GT |
| R1 | 414 | G/T |  | GG | GG | GG |  | TT | TT | TT | GG |  | GT |
|  |  |  |  |  |  |  |  |  |  |  |  |  |  |
| R1026 | 459 | A/C | No, TCA/TCC, Ser | CC | CC | CC | AC | CC | AA | AC | CC |  | CC |
| R596 | 459 | A/C |  | CC | CC | CC | AC | CC | AA | AC | CC | CC | CC |
| 5pr_F | 459 | A/C |  | CC | CC | CC | AC | CC | AA | AC | CC | CC | CC |
| F314 | 459 | A/C |  | CC | CC | CC | AC | CC | AA | AC | CC |  | CC |
| R1 | 459 | A/C |  | CC | CC | CC |  | CC | AA | AC | CC |  | CC |
|  |  |  |  |  |  |  |  |  |  |  |  |  |  |
| R1026 | 558 | T/C | No, TGT/TGC, Cys | TT | TT | TT | TT | CC | TT | TT | TT |  | TT |
| 5pr_F | 558 | T/C |  | TT | TT | TT | TT | CC | TT | TT | TT | TT | TT |
| F314 | 558 | T/C |  | TT | TT | TT | TT | CC | TT | TT | TT |  | TT |
| R1 | 558 | T/C |  | TT | TT | TT |  | CC | TT | TT | TT |  | TT |
|  |  |  |  |  |  |  |  |  |  |  |  |  |  |
| R1026 | 688 | A/T | Yes, ACC/TCC, Thr/Ser | AA | AA | AA | AA | AA | AA | AA | AA |  | AT |
| 5pr_F | 688 | A/T |  | AA | AA | AA | AA | AA | AA | AA | AA | AA | AT |
| F314 | 688 | A/T |  | AA | AA | AA | AA | AA | AA | AA | AA |  | AT |
| R1 | 688 | A/T |  | AA | AA | AA |  | AA | AA | AA | AA |  | AT |

Table S4 continues, next page

**TABLE S4 |** continued

| **LDC primer** | **Position^a^** | **SNP^b^** | **AA change^c^** | **Genotype of *S. flavescens* individuals**^d^ | | | | | | | | | |
| --- | --- | --- | --- | --- | --- | --- | --- | --- | --- | --- | --- | --- | --- |
|  |  |  |  | **1_12** | **2_11** | **3_5** | **4_15^e^** | **5_13** | **6_16** | **7_10** | **8_11** | **9_8^e^** | **10_4** |
| R1026 | 776 | T/C | Yes, ATG/ACG, Met/Thr | TT | TT | TT | TT | TT | TT | TC | TT |  | TT |
| F752 | 776 | T/C |  | TT | TT | TT | TT | TT | TT | TC | TT | TT | TT |
| 5pr_F | 776 | T/C |  | TT | TT | TT | TT | TT | TT | TC | TT | TT | TT |
| F314 | 776 | T/C |  | TT | TT | TT | TT | TT | TT | TC | TT |  | TT |
| R1 | 776 | T/C |  | TT | TT | TT |  | TT | TT | TC | TT |  | TT |
|  |  |  |  |  |  |  |  |  |  |  |  |  |  |
| R1026 | 887 | A/G | Yes, CAA/CGA, Gln/Arg | AA | AA | AA | AA | AA | AA | AG | AA |  | AA |
| F752 | 887 | A/G |  | AA | AA | AA | AA | AA | AA | AG | AA | AA | AA |
| 5pr_F | 887 | A/G |  | AA | AA | AA | AA | AA | AA | AG | AA | AA | AA |
| F314 | 887 | A/G |  | AA | AA | AA | AA | AA | AA | AG | AA |  | AA |
| R1 | 887 | A/G |  | AA | AA | AA |  | AA | AA | AG | AA |  | AA |
|  |  |  |  |  |  |  |  |  |  |  |  |  |  |
| F752 | 1167 | T/C | No, TGT/TGC, Cys | CC | CC | CC | TC | CC | CC | CC | CC | TT | CC |
| F314 | 1167 | T/C |  | CC | CC | CC | TC | CC | CC | CC | CC |  | CC |
|  |  |  |  |  |  |  |  |  |  |  |  |  |  |
| F752 | 1173 | G/A | No, TCG/TCA, Ser | AA | AA | AA | AG | AA | AA | AA | AA | GG | AA |
| F314 | 1173 | G/A |  | AA | AA | AA | AG | AA | AA | AA | AA |  | AA |
|  |  |  |  |  |  |  |  |  |  |  |  |  |  |
| F752 | 1214 | A/G | Yes, GAA/GGA, Glu/Gly | GG | GG | GG | AG | GG | GG | GG | GG | AA | GG |
| F314 | 1214 | A/G |  | GG | GG | GG | AG | GG | GG | GG | GG |  | GG |
|  |  |  |  |  |  |  |  |  |  |  |  |  |  |
| F752 | 1334 | G/C | Yes, GGG/GCG, Gly/Ala | CC | CC | CC | GG | CC | CC | CC | CC | GG | CC |
| F314 | 1334 | G/C |  | CC | CC | CC | GG | CC | CC | CC | CC |  | CC |
|  |  |  |  |  |  |  |  |  |  |  |  |  |  |
| F752 | 1341 | A/G | No, GAA/GAG, Glu | AA | AA | AA | AA | AA | GG | AG | AA | AA | AG |
| R1 | 1341 | A/G |  | AA | AA | AA |  | AA | GG | AG | AA |  | AG |
| F314 | 1341 | A/G |  | AA | AA | AA | AA | AA | GG | AG | AA |  | AG |
|  |  |  |  |  |  |  |  |  |  |  |  |  |  |
| F752 | 1364 | T/C | Yes, GTG/GCG, Val/Ala | CC | CC | CC | TT | CC | CC | CC | CC | TT | CC |
| F314 | 1364 | T/C |  | CC | CC | CC | TT | CC | CC | CC | CC |  | CC |

^a^ Position number is relative to the start of the sequence of the genomic PCR product (excluding primers, SfLDC_F_5pr and SfLDC_R1). The coding sequence starts at position 46. The full sequence for each sample is in **Figure S4**, and aligned (**Figure S7A**) with deduced proteins aligned (**Figure S7B**).

^b^ The first nucleotide is the one present in *SfLDC* (AB561138.1).

^c^ Amino acid (AA) change (Yes or No) relative to *SfLDC* (AB561138.1), followed by the codon in AB561138.1, compared to the alternative codon, and the resulting amino acid(s).

^d^ DNA samples used for PCR and sequenced (Sanger) were the same as used for DArTSeq, one from each region and one commercial sample (**Figure 2**). Grey shading indicates no sequence data available for this genotype/primer combination.

^e^ 4_15 and 9_8 data were generated from three different PCR products as no products were obtained with an annealing temperature of 65°C (1. primers F314 and R1 @ 55°C; 2, primers Fd and R1 @ 55°C and 3, primers Fd and R1 with touch down PCR) and the SNP results were consistent. Sequencing of 4_15 and 9_8 with primer F752 confirmed that primer R1 sequence is different to the other samples (data not shown).

**TABLE S5 |** Genotype information for SNPs identified in *SfCAO* 5' gene fragment amplified from 10 *S. flavecens* samples.

| **CAO primer** | **Position^a^** | **SNP** | **Codons, AA encoded^b^** | **Genotype of *S. flavescens* individuals**^c^ | | | | | | | | | |
| --- | --- | --- | --- | --- | --- | --- | --- | --- | --- | --- | --- | --- | --- |
|  |  |  |  | **1_12** | **2_11** | **3_5** | **4_15** | **5_13** | **6_16** | **7_10** | **8_11** | **9_8** | **10_4** |
| 5'_Fd | 81 | CT | CCC/TCC, Pro/Ser | CT | CT | CT | CT | CT | CT | CT | CT | CT | CT |
| e1_R | 81 | CT |  | CT | CT | CT | CT | CT | CT | CT | CT | CT | CT |
|  |  |  |  |  |  |  |  |  |  |  |  |  |  |
| 5'_Fd | 220 | GT | CGC/CTC, Arg/Leu | GG | GT | GT | GT | GT | GT | GT | GT | GT | GT |
| e1_R | 220 | GT |  | GG | GT | GT | GT | GT | GT | GT | GT | GT | GT |

^a^ Position number is relative to the start of the sequence of the genomic 5' PCR product (excluding primers SfCAO_5p_Fd and SfCAO_e1_R) (**Table S1**). All samples were heterozygous for the SNP at positions 81 and most at 220 (except 1_12), suggesting that two different genes have been amplified. The sequence for each sample is in **Figure S4**, and aligned (**Figure S7C**).

^b^ The two possible codons encode different amino acids (AA).

^c^ DNA samples used for PCR and sequenced (Sanger) were the same as used for DArTSeq, one from each region and one commercial sample (**Figure 2**).

**TABLE S6 |** Genotype information for SNPs identified in *SfCAO1* 3' gene fragment amplified from 10 *S. flavecens* samples.

| **Primer** | ***SfCAO*1 position^a^** | **SNP or polyN** | **AA change^b^** | **Genotype of *S. flavescens* individuals**^c^ | | | | | | | | | |
| --- | --- | --- | --- | --- | --- | --- | --- | --- | --- | --- | --- | --- | --- |
|  |  |  |  | **1_12** | **2_11** | **3_5** | **4_15^d^** | **5_13** | **6_16** | **7_10** | **8_11** | **9_8^d^** | **10_4** |
| e10F | 56 | A_5-6_ | intron | A_6_A_6_ |  | A_5_A_6_ |  | A_5_A_5_ |  |  | A_5_A_5_ | A_5_A_5_ | A_6_A_6_ |
| R1 | 56^e^ | A_5-6_ |  | A_6_A_6_ |  |  |  | A_6_A_6_ |  |  | A_6_A_6_ | A_6_A_6_ |  |
| e11F | 56^e^ | A_5-6_ |  |  |  |  |  |  |  |  |  |  |  |
| e11R | 56^e^ | A_5-6_ |  |  | A_6_A_6_ |  |  |  | A_6_A_6_ | A_6_A_6_ | A_6_A_6_ |  | A_6_A_6_ |
|  |  |  |  |  |  |  |  |  |  |  |  |  |  |
| e10F | 63 | AT | intron | AA |  | AA |  | AA |  |  | TT | TT |  |
| R1 | 63 | AT |  | AA |  | AA |  | AA |  |  | TT | TT |  |
| e11F | 63 | AT |  |  |  |  |  |  |  |  |  |  |  |
| e11R | 63 | AT |  |  | AA | AA |  |  | AA | AA | TT |  | AA |
|  |  |  |  |  |  |  |  |  |  |  |  |  |  |
| e10F | 68 | AG | intron | AA |  |  |  | AA |  |  | GG | GG |  |
| R1 | 68 | AG |  | AA |  |  |  | AA |  |  | GG |  |  |
| e11F | 68 | AG |  |  |  |  |  |  |  |  |  |  |  |
| e11R | 68 | AG |  |  | GG |  |  |  | GG | GG | GG |  | GG |
|  |  |  |  |  |  |  |  |  |  |  |  |  |  |
| e10F | 69 | CT | intron | CC |  |  |  | CC |  |  | TT | TT |  |
| R1 | 69 | CT |  | CC |  |  |  | CC |  |  | TT |  |  |
| e11F | 69 | CT |  |  |  |  |  |  |  |  |  |  |  |
| e11R | 69 | CT |  |  | TT |  |  |  | CT | CT | TT | TT | TT |
|  |  |  |  |  |  |  |  |  |  |  |  |  |  |
| e10F | 75 | AG | intron | AA |  |  |  | AA |  |  | GG | GG |  |
| R1 | 75 | AG |  | AA |  |  |  | AA |  |  | GG | GG |  |
| e11F | 75 | AG |  |  |  |  |  |  |  |  |  |  |  |
| e11R | 75 | AG |  |  | AG |  |  |  | AG | AG | GG |  | AG |
|  |  |  |  |  |  |  |  |  |  |  |  |  |  |
| e10F | 137 | GT | intron | GG |  | GG |  | GG |  |  | TT | TT |  |
| R1 | 137 | GT |  | GG |  | GG |  | GG |  |  | TT | TT |  |
| e11F | 137 | GT |  |  |  |  |  |  |  |  |  |  |  |
| e11R | 137 | GT |  |  | GG | GG |  |  | GT | GG | TT |  | GG |
|  |  |  |  |  |  |  |  |  |  |  |  |  |  |
| e10F | 182 | CT | intron | CC |  | CC |  | CC |  |  | CC | CC |  |
| R1 | 182 | CT |  | CC |  | CC |  | CC |  |  | CC | CC |  |
| e11F | 182 | CT |  |  |  |  |  |  |  |  |  |  |  |
| e11R | 182 | CT |  |  | CT | CC |  |  | CC | CT | CC |  | CC |

Table S6 continues, next page

**TABLE S6 |** continued

| **Primer** | ***SfCAO1* position^a^** | **SNP or polyN** | **AA change^b^** | **Genotype of *S. flavescens* individuals**^c^ | | | | | | | | | |
| --- | --- | --- | --- | --- | --- | --- | --- | --- | --- | --- | --- | --- | --- |
|  |  |  |  | **1_12** | **2_11** | **3_5** | **4_15^d^** | **5_13** | **6_16** | **7_10** | **8_11** | **9_8^d^** | **10_4** |
| e10F | 322 | TC | No, CCT/CCC, Pro | TT |  | TT |  | TT |  |  | CC |  |  |
| R1 | 322 | TC |  | TT |  | TT |  | TT |  |  | CC |  |  |
| e11F | 322 | TC |  |  |  |  |  |  | TT | TT |  |  | TT |
| e11R | 322 | TC |  |  |  |  |  |  |  |  |  |  |  |
|  |  |  |  |  |  |  |  |  |  |  |  |  |  |
| e10F | 335 | GA | Yes, GAG/AAG, Glu/Lys | GG |  | AA |  | GG |  |  | GG |  |  |
| R1 | 335 | GA |  | GG |  | AA |  | GG |  |  | GG |  |  |
| e11F | 335 | GA |  |  | GG | AA |  |  | GG | GG | GG |  | GG |
| e11R | 335 | GA |  |  |  |  |  |  |  |  |  |  |  |
|  |  |  |  |  |  |  |  |  |  |  |  |  |  |
| e10F | 386-394^f^ | T_8-9_ | intron | A_8_A_8_ |  |  |  | A_8_A_8_ |  |  | A_9_A_9_ |  |  |
| R1 | 386-394^f^ | T_8-9_ |  | A_8_A_8_ | A_8_A_9_ | A_8_A_8_ |  | A_8_A_8_ | A_8_A_9_ | A_8_A_9_ | A_9_A_9_ |  | A_8_A_9_ |
| e11F | 386-394^f^ | T_8-9_ |  |  | A_8_A_9_ | A_8_A_8_ |  |  | A_8_A_9_ | A_8_A_9_ | A_9_A_9_ |  | A_8_A_9_ |
| e11R | 386-394^f^ | T_8-9_ |  |  |  |  |  |  |  |  |  |  |  |
|  |  |  |  |  |  |  |  |  |  |  |  |  |  |
| e10F | 423 | CA | intron | CC |  | CC |  | CC |  |  | CC |  |  |
| R1 | 423 | CA |  | CC | CC | CC |  | CC | CC | CC | CC |  | AC |
| e11F | 423 | CA |  |  |  | CC |  |  |  |  | CC |  |  |
| e11R | 423 | CA |  |  |  |  |  |  |  |  |  |  |  |
|  |  |  |  |  |  |  |  |  |  |  |  |  |  |
| e10F | 458 | TC | intron | TT |  | TT |  | TT |  |  | CC | CC |  |
| R1 | 458 | TC |  | TT | CT | TT |  | TT | CT | CT | CC | CC | CT |
| e11F | 458 | TC |  |  |  | TT |  |  |  |  | CC |  |  |
| e11R | 458 | TC |  |  |  |  |  |  |  |  |  |  |  |
|  |  |  |  |  |  |  |  |  |  |  |  |  |  |
| e10F | 463 | AG | intron | AA |  | AA |  | AA |  |  | GG | GG |  |
| R1 | 463 | AG |  | AA | AG | AA |  | AA | AG | AG | GG | GG | AG |
| e11F | 463 | AG |  |  |  | AA |  |  |  |  | GG |  |  |
| e11R | 463 | AG |  |  |  |  |  |  |  |  |  |  |  |
|  |  |  |  |  |  |  |  |  |  |  |  |  |  |
| e10F | 474 | TG | intron | TT |  | TT |  | TT |  |  | TT | TT |  |
| R1 | 474 | TG |  | TT | TG | TT |  | TT | TT | TG | TT | TT | TT |
| e11F | 474 | TG |  |  |  | TT |  |  |  |  | TT |  |  |
| e11R | 474 | TG |  |  |  |  |  |  |  |  |  |  |  |
|  |  |  |  |  |  |  |  |  |  |  |  |  |  |
| e10F | 544 | GA | No, GTG/GTA, Val | GG |  | GG |  | GG |  |  | GG | GG |  |
| R1 | 544 | GA |  | GG | GG | GG |  | GG | GG | GA | GG | GG | GG |
| e11F | 544 | GA |  |  |  | GG |  |  |  |  | GG |  |  |
| e11R | 544 | GA |  |  |  |  |  |  |  |  |  |  |  |

Table S6 footnotes, next page

**TABLE S6 |** footnotes

^a^ Position number is relative to the start of the sequence of 3' genomic PCR product for *SfCAO1* (excluding primers SfCAO_e10_F and SfCAO_R1) for sample 1_12 (**Table S1**). Samples that were heterozygous for an indel or SSR leading to a frameshift, were sequenced with two additional primers SfCAO_e11_F and SfCAO_e11_R1. The sequence for each sample is in **Figure S4**, and aligned (**Figure S7D**).

^b^ Amino acid (AA) change (Yes or No) relative to sample 1_12, followed by the codon, compared to the alternative codon, and the resulting amino acid(s).

^c^ DNA samples used for PCR and sequenced (Sanger) were the same as used for DArTSeq, one from each region and one commercial sample (**Figure 2**). Grey shading indicates no sequence data available for this genotype/primer combination.

^d^ Sample 4_15 did not produce a PCR product for *SfCAO1* and sample 9_8 produced a smaller ‘lower’ PCR product (**Figure S9A**) that was later revealed as a 181 nt deletion from intron 10 to intron 11, including all of exon 11 (nucleotides 271..451) **(Figure S7)** and are shaded black.

^e^ For this polyA tract different results were obtained with forward and reverse primers. Where this occurred (eg samples 5_13, 8_11 and 9_8), sequences were assumed to be homozygous for the longest allele eg A6 (underlined). Samples heterozygous for the polyA tract produced a frameshift that resulted in unreadable downstream sequence.

^f^ For this polyT tract different results were obtained with forward and reverse primers. Where this occurred sequences were assumed to be homozygous for the longest allele Samples heterozygous for the polyT tract produced a frameshift that resulted in unreadable downstream sequence.

**TABLE S7 |** Genotype information for SNPs identified in *SfCAO2* 3' gene fragments amplified from 10 *S. flavecens* samples.

| **Primer** | ***SfCAO*2 position^a^** | **SNP or polyN** | **AA change^b^** | **Genotype of *S. flavescens* individuals**^c^ | | | | | | | | | |
| --- | --- | --- | --- | --- | --- | --- | --- | --- | --- | --- | --- | --- | --- |
|  |  |  |  | **1_12** | **2_11** | **3_5** | **4_15** | **5_13** | **6_16** | **7_10** | **8_11** | **9_8** | **10_4** |
| e10F | 69 | AG | intron |  | AA | AA |  | AG |  | AA |  | AA |  |
| R1 | 69 | AG | intron | AA | AA | AA |  |  | AA | AA |  | AA |  |
| e11F | 69 | AG | intron |  |  |  |  |  |  |  |  |  |  |
| e11R | 69 | AG | intron | AA |  | AA | AG | AG | AA |  | AG |  |  |
|  |  |  |  |  |  |  |  |  |  |  |  |  |  |
| e10F | 79 | TC | intron |  | TT | TT |  | TC |  | TT |  | TT |  |
| R1 | 79 | TC | intron | TT | TT | TT |  |  | TT | TT |  | TT |  |
| e11F | 79 | TC | intron |  |  |  |  |  |  |  |  |  |  |
| e11R | 79 | TC | intron | TT |  | TT | TC | TC | TT |  | TC |  |  |
|  |  |  |  |  |  |  |  |  |  |  |  |  |  |
| e10F | 235 | TC | intron |  | TT | TT |  | TC |  | TT |  | TT |  |
| R1 | 235 | TC | intron | TT | TT | TT |  |  | TT | TT |  | TT |  |
| e11F | 235 | TC | intron |  |  |  |  |  |  |  |  |  |  |
| e11R | 235 | TC | intron | TT |  | TT | TC | TC | TT |  | TC |  | TC |
|  |  |  |  |  |  |  |  |  |  |  |  |  |  |
| e10F | 736 | TC | intron |  | TT | TT |  |  |  | TT |  | TT |  |
| R1 | 736 | TC | intron | TT | TT | TT | TC | TC | TT | TT | TC | TT | TC |
| e11F | 736 | TC | intron | TT |  | TT |  |  | TT |  |  |  |  |
| e11R | 736 | TC | intron |  |  |  |  |  |  |  |  |  |  |
|  |  |  |  |  |  |  |  |  |  |  |  |  |  |
| e10F | 801 | GA | intron |  | AA | AA |  |  |  | GA |  | AA |  |
| R1 | 801 | GA | intron | GG | AA | AA | AA | AA | GA | GA | AA | AA | AA |
| e11F | 801 | GA | intron | GG |  | AA |  |  |  |  |  |  |  |
| e11R | 801 | GA | intron |  |  |  |  |  |  |  |  |  |  |
|  |  |  |  |  |  |  |  |  |  |  |  |  |  |
| e10F | 823 | GA | intron |  | GG | AA |  |  |  | GG |  | AA |  |
| R1 | 823 | GA | intron | GG | GG | AA | AA | GA | AA | GG | AA | AA | AA |
| e11F | 823 | GA | intron | GG |  | AA |  |  |  |  |  |  |  |
| e11R | 823 | GA | intron |  |  |  |  |  |  |  |  |  |  |
|  |  |  |  |  |  |  |  |  |  |  |  |  |  |
| e10F | 909 | CT | intron |  | CC | CC |  |  |  | CC |  | CC |  |
| R1 | 909 | CT | intron | CC | CC | CC | TC | TC | CC | CC | TC | CC | CC |
| e11F | 909 | CT | intron | CC |  | CC |  |  | CC |  |  |  | CC |
| e11R | 909 | CT | intron |  |  |  |  |  |  |  |  |  |  |

^a^ Position number is relative to start of the sequence of *SfCAO2* genomic 3' PCR product (excluding primers SfCAO_e10_F and SfCAO_R1) for sample 1_12 (**Table S1**). Samples that were heterozygous for an indel or SSR leading to a frameshift, were sequenced with two additional primers SfCAO_e11_F and SfCAO_e11_R1. The sequence for each sample is in **Figure S4**, and aligned (**Figure S7E**).

^b^ Amino acid (AA) change (Yes or No) relative to sample 1_12, followed by the codon, compared to the alternative codon, and the resulting AAs.

^c^ DNA samples used for PCR and sequenced (Sanger) were the same as used for DArTSeq, one from each region and one commercial sample (**Figure 2**). Grey shading indicates no sequence data available for this genotype/primer combination.

**TABLE S8 |** Lengths of the simple sequence repeat (SSR) polymorphisms in intron 10 are different in *SfCAO1* and *SfCAO2.* Samples that are heterozygous for SSR length do not produce readable sequence after the SSR (**Figure S10**).

|  | **SSR genotype of sequenced individuals** | | | | | | | | | |
| --- | --- | --- | --- | --- | --- | --- | --- | --- | --- | --- |
| Individual | 1_12 | 2_11 | 3_5 | 4_15 | 5_13 | 6_16 | 7_10 | 8_11 | 9_8 | 10_4 |
| *SfCAO1* | (CT)_7_ | (CT)_6_/(CT)_7_ | (CT)_8_ | no product | (CT)_7_ | (CT)_6_ /(CT)_7_ | (CT)_6_ /(CT)_7_ | (CT)_6_ | (CT)_6_ | (CT)_6_ /(CT)_7_ |
| *SfCAO2* | (CT)_8_/(CT)_9_ | (CT)_8_ | (CT)_8_ | (CT)_7_/(CT)_8_ | (CT)_8_ | (CT)_7_/(CT)_8_ | (CT)_8_ | (CT)_7_/(CT)_8_ | (CT)_8_ | (CT)_7_/(CT)_8_ |

**TABLE S9 |** SNPs identified in *SfCAO* cDNA fragments amplified from the two *S. flavecens* plants used for qPCR (**Figure 7**) compared to each other and to the *SfCAO* sequence assembled from RNASeq data. SNP positions in bold indicate a difference between 1_LC1 and 9_AC1. Green shading indicates that both 1_LC1 and 9_AC1 have an amino acid change compared to the sequence assembled from the short reads (*SfCAO_cDNA_mirabait2_81_c5,* **Figure S4**). Red text indicates the polymorphic position.

|  | SNP position | | | | | | | | | | | | | | |
| --- | --- | --- | --- | --- | --- | --- | --- | --- | --- | --- | --- | --- | --- | --- | --- |
|  | 116 | 579 | 607 | 671 | **785** | **794** | 1192 | 1304 | 1396 | 1622 | **1724** | 1828 | 1889 | **1928** | **2000** |
| SNP | A/T | T/A | C/T | G/T | A/C | T/C | T/A | C/T | G/A | C/T | T/G | C/G | C/T | A/G | C/T |
| Mira codon | TCA | TCT | GCA | GAG | GTA | GAT | GTT | GCC | AGT | ATC | CGT | GCA | CCC | TCA | TCC |
| Mira AA | Ser | Ser | Ala | Glu | Val | Asp | Val | Ala | Ser | Ile | Arg | Ala | Pro | Ser | Ser |
|  |  |  |  |  |  |  |  |  |  |  |  |  |  |  |  |
| 1_LC1 codon | TCT | ACT | GTA | GAT | GTC | GAC | GAT | GCT | AAT | ATT | CGT | GGA | CCT | TCA | TCT |
| 1_LC1 AA | Ser | Thr | Val | Asp | Val | Asp | Asp | Ala | Asn | Ile | Arg | Gly | Pro | Ser | Ser |
|  |  |  |  |  |  |  |  |  |  |  |  |  |  |  |  |
| 9_AC1 codon | TCT | ACT | GTA | GAT | GTA | GAT | GAT | GCT | AAT | ATT | CGG | GCA | CCC | TCG | TCC |
| 9_AC1 AA | Ser | Thr | Val | Asp | Val | Asp | Asp | Ala | Asn | Ile | Arg | Ala | Pro | Ser | Ser |

# TABLE S10 | for full size - see separate excel file (legend and thumbnail data below)

**TABLE S10** | Gene-specific KASP markers for *SfLDC* and *SfCAO1*: Genotype of 87 samples from 9 regions for 7 SfLDC-specific markers and 3 SfCAO1-specific markers. The sample from each region that was sequenced with Sanger sequencing is indicated in blue bold text and genotypes that did not match the Sanger result are underlined (see **Tables S4** and **S6** for *SfLDC* and *SfCAO1*, respectively). nr, no result.

#
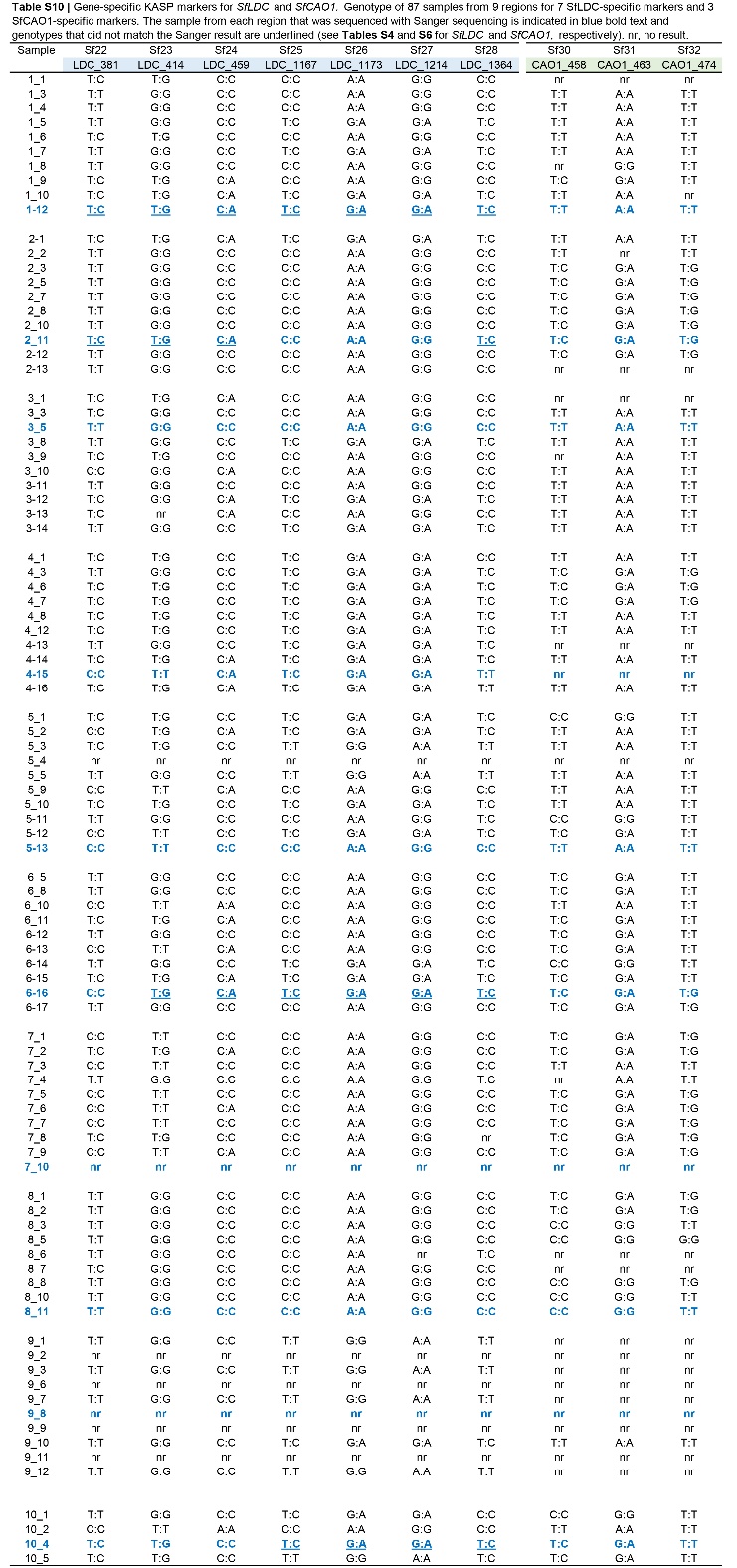


# Supplementary Figures

See next page

| **A**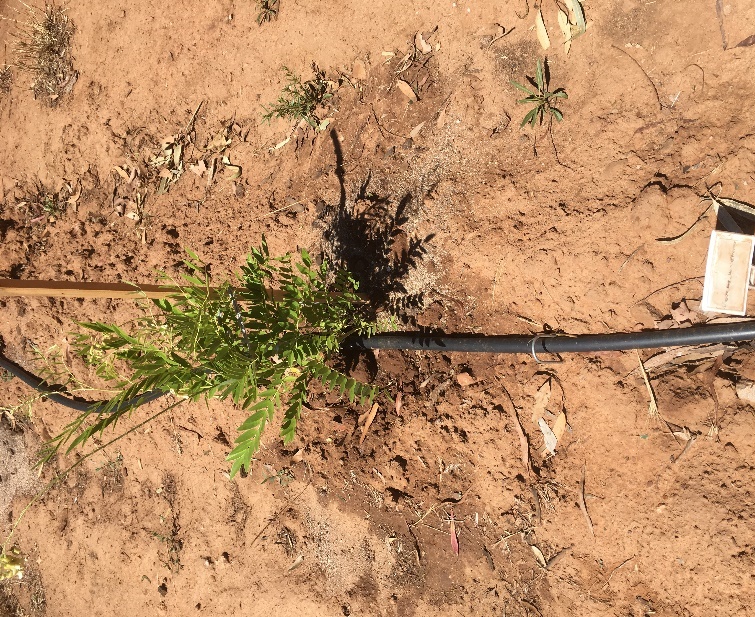 | **B**  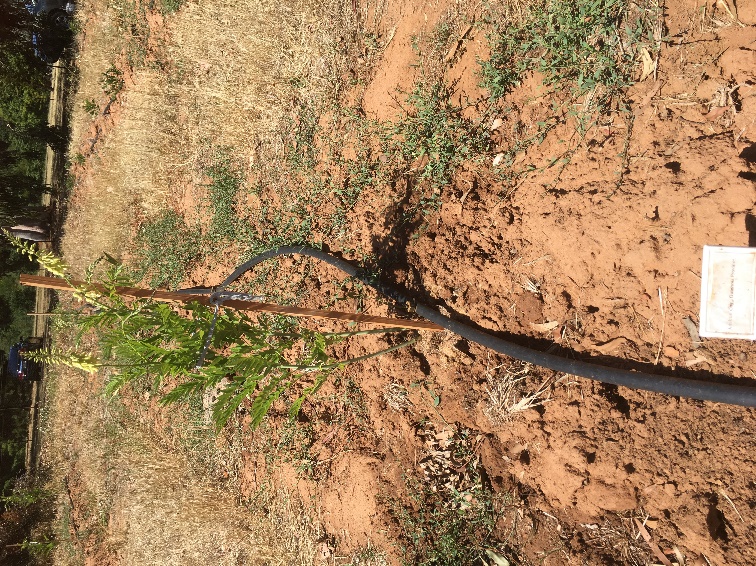 |
| --- | --- |
| **C**  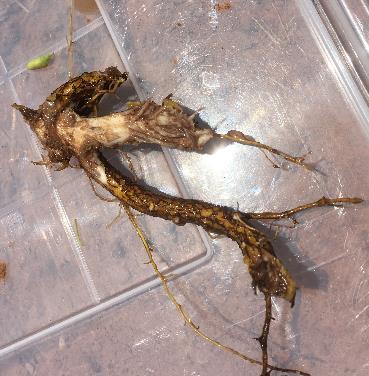 | **D**  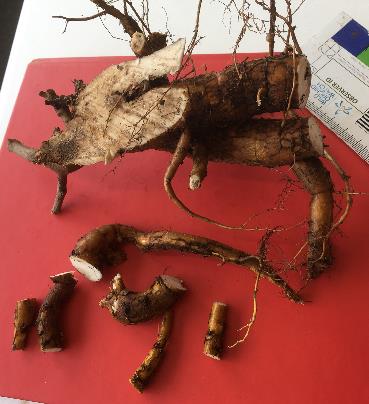 |

**FIGURE S1 |** Plants prior to harvest **(A,B)** and photographs of the harvested root pieces **(C,D)**. **(A)** Plant 1_LC1, grown from seed collected in Lingyuan City, Liaoning Province (growing in the field trial site, position row 1, plant 1N (north)). **(B)** Plant 9_AC1, grown from seed collected in from Anshun City, Guizhou Province (field trial site position row 9, plant 1N). **(C)** Photograph of the harvested tissue of 1_LC1, which was sampled from the lower part of the root system to ensure survival of LC1. The ‘root-big’ tissue (centre, whiter portion) was not as big as expected based on the ‘sacrificed’/practice plant, but was thicker than the ‘root-small’ (left). **(D)** The root piece removed from plant 9_AC1 was much larger than removed from plant 1_LC, because the latter had a much smaller root system (see portion removed (**C**)). Root pieces were photographed after tissue sampling for qPCR.

| **Tissue** | **Images** | | **Tissue collection details** |  |
| --- | --- | --- | --- | --- |
| **Leaves** | 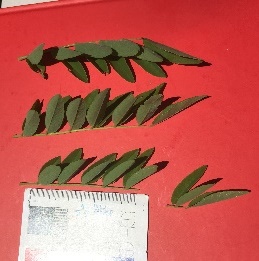  **Leaflets** | 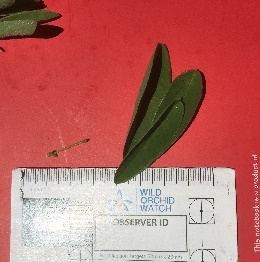  **Leaves** | Leaf samples. Used 3 apical leaves (fully expanded) from a leaflet approximately 30-40 cm off the ground. Each replicate (rep, *n*=3*)* represents leaves from a different leaflet. |  |
|  |  | |  |  |
| **Stem** | 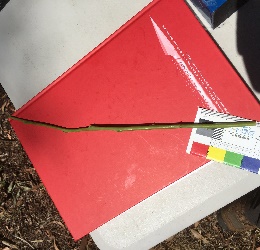 | 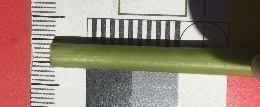 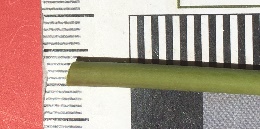 | Stem samples. Cut off a section of stem about 40 cm in length, about 40 cm above the ground. Leaves were removed and then 6 pieces (8 mm in length) were cut from each end of the stem. Pieces (from each end) 1 and 4 (rep 1), 2 and 5 (rep 2) and 3 and 6 (rep 3) for a total of 4 pieces for each replicate. |  |
|  | **stem piece (left)** | **stem thick, thin end (right)** |  |  |
|  |  | |  |  |
| **Roots with**  **stems attached** | 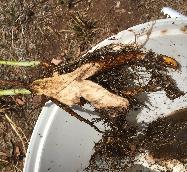 | 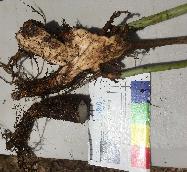 | Root samples. A section of root was selected and pieces cut from either end, as per stem. Small roots were cut into 5 – 7 mm pieces, and big roots were cut in half, then slices taken (2 – 3 mm). |  |
| **Roots pieces** | 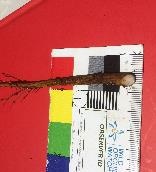 | 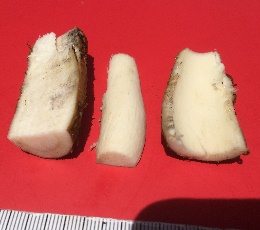 |  |  |
|  | **root – small (s)** | **root – big (b)** |  |  |
|  |  | |  |  |
| **Flowers** | 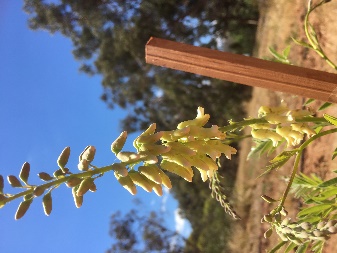 | 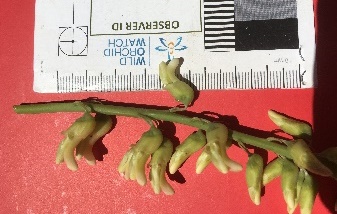 | Flower samples. A mix of open flowers and buds (including pedicels) were used as follows. Three in total, one fully opened, one with 1 – 3 mm of petal showing and one unopened. |  |
|  |  | |  |  |

**FIGURE S2 |** Photos of a test plant used to develop a consistent harvesting strategy. A description of how each tissue type was collected is provided adjacent the relevant photos.

**A B**


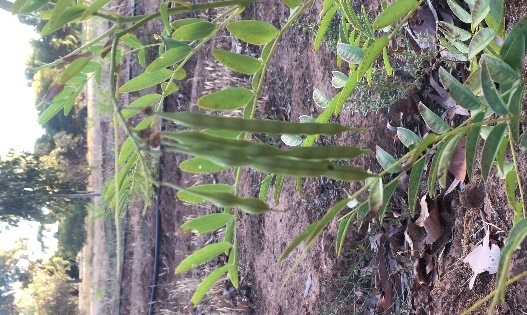

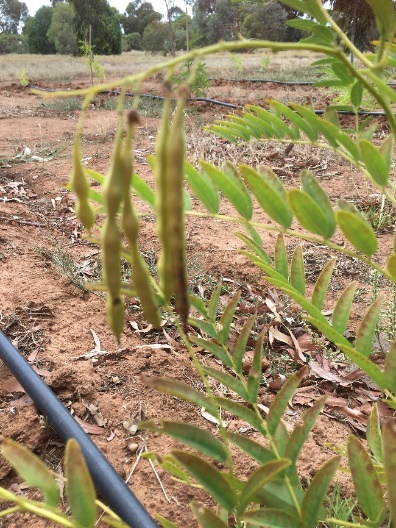


**C D E**


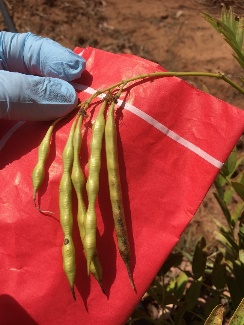

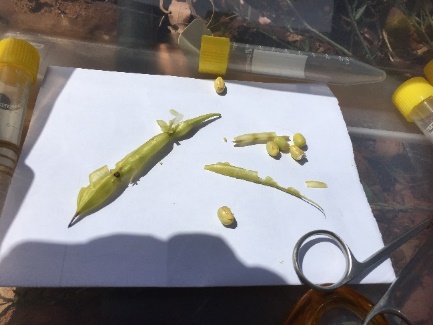

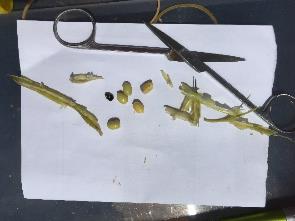


**FIGURE S3 |** Developing seed pods on plant 1_LC4 (grown from seed collected in Lingyuan City, Liaoning Province (field trial site position row 1, plant 4N from the northern end of row)). (**A**) Developing seed pods 13 days before harvest (30 Jan 2019). **(B)** and **(C)**. Pods harvested 12 February 2019. **(D)** Pod a (rep 1) had 6 yellowish-green plump seeds. **(E)** Pod b (rep 2) had 5 good looking plump seed, and one black / wrinkled seed that was discarded, and Pod c (rep 3) had 6 good plump seeds (not shown). The pods could not be opened easily and had to be cut / torn to release the seed. The pod pieces from each replicate were pooled separately to give the three replicate samples of pod material.

**FIGURE S4 |** *S. flavescens* DNA sequences obtained during this study*.*

**See separate Word file (49 sequences) – figure legend include here for context**

(**A**) *S. flavescens* sequences assembled from short read RNA-seq data after two rounds of mirabait (*SfCAO*, *SfLDC* and 5 control genes). Two RNAseq datasets were used from *S. flavescens* project (PRJDB3906, DRR031281 and DRR031283, referred to as 81 and 83, respectively in the sequencing header line) (Han et al., 2015). For *SfLDC* two variants were obtained variant 1 (from dataset DRR031281) and variant 2 (from dataset DRR031283). There are 5 SNP between the two sequences and three of them correspond to the highly polymorphic SNPs observed from sequencing the 10 *SfLDC* genes (positions 41, 381 and 459, **Figure S7**). Some of the cDNA sequences for the control genes for qPCR are partial sequences that include the 3' untranslated region. Selected partial sequences start with N or NN to give the correct reading frame for translation of uppercase text. Includes a total of 8 sequences.

**(B)** *SfLDC* full length genomic DNA sequence derived from Sanger sequenced PCR products amplified using SfLDC_F_5pr and SfLDC_R1, 10 sequences, one from each region 1 to 10).

**(C)** *SfCAO* 5' partial genomic DNA sequence derived from Sanger sequenced PCR products amplified using SfCAO_5p_Fd and SfCAO_e1_R, 10 sequences, one from each region 1 to 10).

**(D)** *SfCAO1* 3' partial genomic DNA derived from (Sanger sequenced PCR products amplified using SfCAO_e10_F and SfCAO_R1, 9 sequences, one from each region 1 to 10, except region 4 where no product was amplified from sample 4_15).

**(E)** *SfCAO2* 3' partial genomic DNA derived from Sanger sequenced PCR products amplified using SfCAO_e10_F and SfCAO_R1, 10 sequences, one from each region 1 to 10).

**(F)** *SfCAO* full length cDNA sequences derived from Sanger sequenced PCR products amplified using SfCAO_5p_Fd and SfCAO_R1, 2 sequences from samples collected from the field trial site, 1_LC1 and 9_AC1 respectively).

Upper case text is coding sequence, lowercase text is non-coding sequence (eg untranslated regions (UTRs) or introns. Unterlined text in introns indicates a region that is polymorphic in this individual for an SSR or polyN tract, creating a frameshift. Primer sequences are listed in **Table S1**.

Sequences from the “wet-bench experiments” (Fig. S4B Fig. S4F) were submitted to GenBank, NCBI accession numbers, MW960974-MW961014 (MW960974- MW960975 (Fig. S4F), MW960976- MW960985 (Fig. S4F), MW960986- MW960994 (Fig. S4F), MW960995- MW961004 (Fig. S4F), MW961005-MW961014 (Fig. S4F).

SaLDC_5UTR_KY038928.1 CACCGCTGCAATCCATCATATGCGTTCCTATAAATAGGGTCGTCATTT**A**TGTTCTCCAACAT

SfLDC_mirabait2_81_c1rc --------------------------------------------------------------

DRR031281.5306439.1 --------------------TGCGTTCCTATAAATAGGGTCGTCATTTGTGTTCTCCAACAT

DRR031281.13238458.1 CACCGCTGCAATCCATCATATGCGTTCCTAT-AATAGGGTCGTCATTTGTGTTCTCCAACAT

DRR031281.3994782.1 --------------ATCATATGCGTTCCTATAAATAGGGTCGTCATTTGTGTTCTCCAACAT

**Primer SfLDC_F_5pr AGGGTCGTCATTTGTGTTCTCC**

SaLDC_5UTR_KY038928.1 cccagttgaaatcacagtccttcacccaaaattaataataca**ATG**----------------

SfLDC_mirabait2_81_c1rc cccagttgaaatcacagtccttcacccaaaattaata--aaa**ATG**----------------

DRR031281.5306439.1 cccagttgaaatcacagtccttcacccaaaattaata--aaa**ATG**CCTACACTAGTAACTG

DRR031281.13238458.1 cccagttgaaatcacagtccttcacccaaaa------------------------------

DRR031281.3994782.1 cccagttgaaatcacagtccttcacccaaaattaata--aaa**ATG**CCTA------------

*******************************

**FIGURE S5 |** Alignment (Muscle) for *SfLDC* 5' UTR primer design. Additional 5' sequence for primer design to enable sequencing of full length genomic sequences was obtained from the 5' UTR sequence obtained in this study using mirabait (SfLDC_cDNA_variant_1_mirabait2_81_c1rc, **Figure S4**) and sequences from NCBI: promoter sequence from *S. alopecuroides* (KY038928.1, SaLDC_5UTR) and three short reads from *S. flavescens* (DRR031281.13238458.1, DRR031281.3994782.1, and DRR031281.5306439.1). Primer sequence (in green) has one mis-match to the *S. alopecuroides* sequence (in red). Start ATG in blue.

**FIGURE S6 |** Example chromatograms showing SNPs. See legend after figure.

*
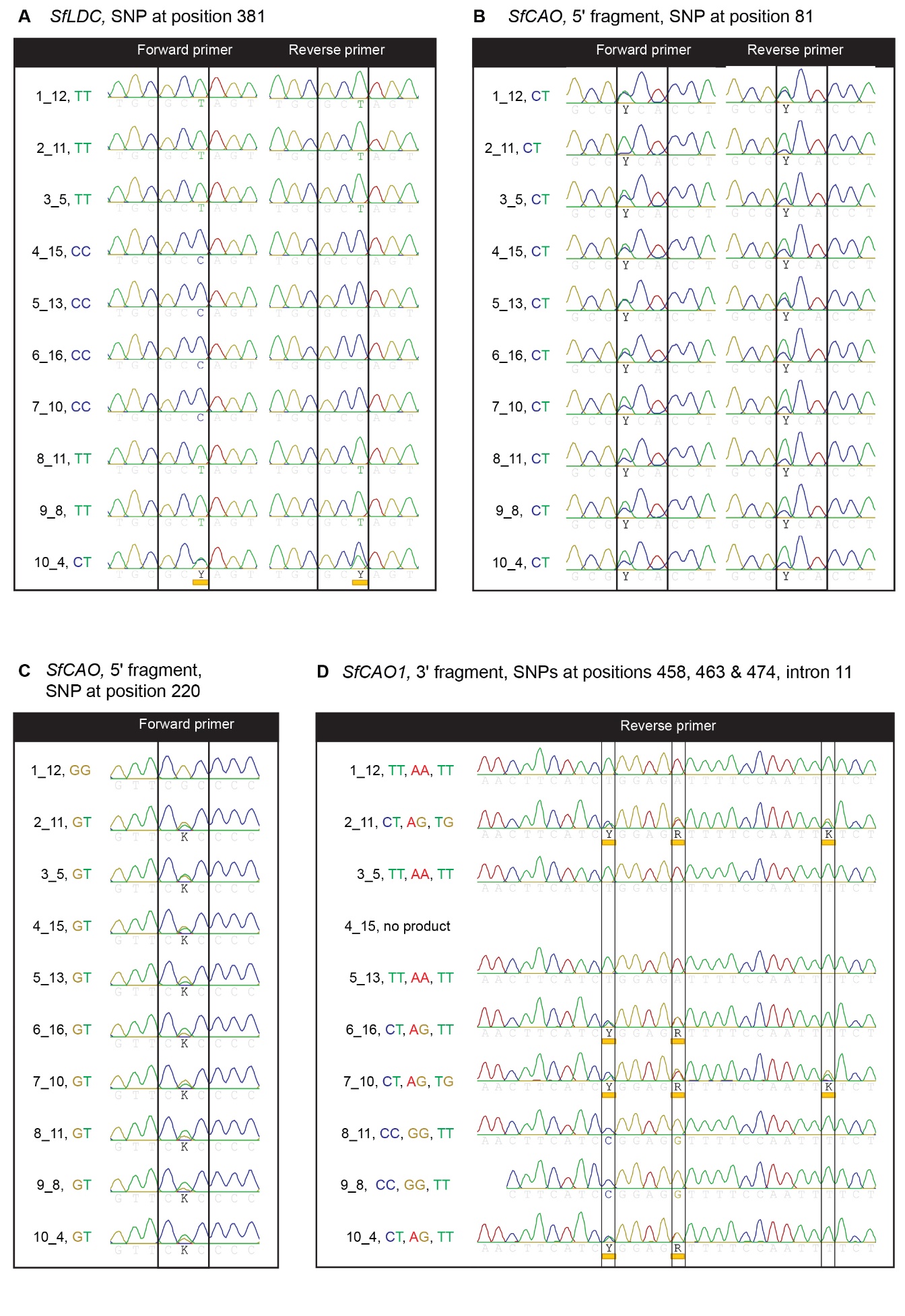
*

**FIGURE S6 |** Example chromatograms showing SNPs identified in *SfLDC* **(A)**, exon 1 of *SfCAO* **(B, C)** and intron 11 of *SfCAO1* **(D)**. Chromatograms from Sanger sequencing of purified PCR products amplified from 10 genomic DNA samples (**Figure 2**). SNPs are summarised in **Tables S2** (*SfLDC*) and **S3** (*SfCAO* 5' PCR product) and **S4** (*SfCAO1* 3' PCR product), respectively. See **Figure S7** for the aligned sequences for each PCR product. Vertical lines delineate codons for chromatograms in **A–C,** whereas in **D** the lines delineate variable nucleotides in intron 11 highlighting 3 SNPs. Solid yellow bars under peaks **(A,D)** indicate heterozygous positions where the sequence was manually changed to the IUPAC degenerate code. **(A)** C/T SNP at position 381 of *SfLDC* PCR product (**Figure 4**) sequenced with both the forward and reverse primers. All possible genotypes were observed, TT, CC and CT. GCT and GCC both encode Ala. **(B)** C/T SNP at position 81 of *SfCAO* 5' PCR product (**Figure 4**) sequenced with both the forward and reverse primers. All samples are heterozygous CT for the SNP, which results in different amino acids, Pro (CCC) and Ser (TCC). **(C)** G/T SNP at position 220 of *SfCAO* 5' PCR product (**Figure 4**), sequenced with the forward primer. Nine of 10 samples are heterozygous for GT, whereas sample 1_12 is homozygous GG. The SNP results in an amino acid change from Arg (CGC) to Leu (CTC). There is no data for the reverse primer because the SNP is too close to the sequence primer. A new primer could not be designed easily because intron 1 sequence for *S. flavescens* is not known, and is expected to be nearly 2 kb, based on the size in lupin (1931nt, see **Figure S8A**). **(D)** Three (of four) SNPs in intron 11 of *SfCAO1* amplified with primer e10_F and R1) (**Figure 4**). Unlike the 5' PCR product **(B,C)**, most of the possible genotypes are observed (with the exception of GG in the third SNP (T/G)). One explanation for the contrasting results between the 5' and 3' SNPs for *SfCAO* is that the 5' PCR product (with no introns) represents the sequence of the two genes *SfCAO1* and *SfCAO2*, that were revealed in the 3' PCR product due to differences in the lengths of both introns 10 and 11 (**Figure S9**). Sequencing data was analysed and edited in Geneious (8.1.9).

**FIGURE S7 |** Alignments of sequences generated from Sanger sequencing of PCR products of genomic DNA (gDNA). *SfLDC* **(A)**, and its encoded protein **(B)** and *SfCAO* **(B–E)**. Samples from nine regions of China and one commercial sample (**Figure 2**). SNPs are highlighted in yellow, or green (for sites with three genotypes). Heterozygous individuals were clearly identified for some SNPs (see **Figure S6** for chromatograms of SNP at position 381), as indicated by the IUPAC code (and **Tables S3–S6**). SNPs numbered in blue (bold) were developed into KASP markers (**Table S9**). SNPs that result in amino acid changes are listed below the alignment **(B)**. Coding sequence, uppercase text; UTRs and introns, lowercase text. N or n (in exon or intron sequence, respectively), is used where the base could not be called. –, is used where no sequence was available (eg deletion variant of *SfCAO1* from sample 9_8), or close to the sequencing primer.

**FIGURE S7A |** *SfLDC* full length gDNA sequence

41

**|**

1_12 aacatcccagttgaaatcacagtccttcacccaaaattaataaaaATGCCTACACTAGTAACTGAGGCATTCCATGCCAAGGGTGCAGGACCTTTGAGCCTGAAGCCACTATTTAGTGCTTCAGGGGTTAAGGGCAAAAGAGTCACTGCATTATCTGCAAAAGAAGAAGGTGGCATCTCT

2_11 aacatcccagttgaaatcacagtccttcacccaaaattaaaaaaaATGCCTACACTAGTAACTGAGGCATTCCATGCCAAGGGTGCAGGACCTTTGAGCCTGAAGCCACTATTTAGTGCTTCAGGGGTTAAGGGCAAAAGAGTCACTGCATTATCTGCAAAAGAAGAAGGTGGCATCTCT

3_5 aacatcccagttgaaatcacagtccttcacccaaaattaataaaaATGCCTACACTAGTAACTGAGGCATTCCATGCCAAGGGTGCAGGACCTTTGAGCCTGAAGCCACTATTTAGTGCTTCAGGGGTTAAGGGCAAAAGAGTCACTGCATTATCTGCAAAAGAAGAAGGTGGCATCTCT

4_15 aacatcccagttgaaatcacagtccttcacccaaaattaataaaaATGCCTACACTAGTAACTGAGGCATTCCATGCCAAGGGTGCAGGACCTTTGAGCCTGAAGCCACTATTTAGTGCTTCAGGGGTTAAGGGCAAAAGAGTCACTGCATTATCTGCAAAAGAAGAAGGTGGCATCTCT

5_13 aacatcccagttgaaatcacagtccttcacccaaaattaataaaaATGCCTACACTAGTAACTGAGGCATTCCATGCCAAGGGTGCAGGACCTTTGAGCCTGAAGCCACTATTTAGTGCTTCAGGGGTTAAGGGCAAAAGAGTCACTGCATTATCTGCAAAAGAAGAAGGTGGCATCTCT

6_16 aacatcccagttgaaatcacagtccttcacccaaaattaataaaaATGCCTACACTAGTAACTGAGGCATTCCATGCCAAGGGTGCAGGACCTTTGAGCCTGAAGCCACTATTTAGTGCTTCAGGGGTTAAGGGCAAAAGAGTCACTGCATTATCTGCAAAAGAAGAAGGTGGCATCTCT

7_10 aacatcccagttgaaatcacagtccttcacccaaaattaawaaaaATGCCTACACTAGTAACTGAGGCATTCCATGCCAAGGGTGCAGGACCTTTGAGCCTGAAGCCACTATTTAGTGCTTCAGGGGTTAAGGGCAAAAGAGTCACTGCATTATCTGCAAAAGAAGAAGGTGGCATCTCT

8_11 aacatcccagttgaaatcacagtccttcacccaaaattaataaaaATGCCTACACTAGTAACTGAGGCATTCCATGCCAAGGGTGCAGGACCTTTGAGCCTGAAGCCACTATTTAGTGCTTCAGGGGTTAAGGGCAAAAGAGTCACTGCATTATCTGCAAAAGAAGAAGGTGGCATCTCT

9_8 aacatcccagttgaaatcacagtccttcacccaaaattaaaaaaaATGCCTACACTAGTAACTGAGGCATTCCATGCCAAGGGTGCAGGACCTTTGAGCCTGAAGCCACTATTTAGTGCTTCAGGGGTTAAGGGCAAAAGAGTCACTGCATTATCTGCAAAAGAAGAAGGTGGCATCTCT

10_4 aacatcccagttgaaatcacagtccttcacccaaaattaawaaaaATGCCTACACTAGTAACTGAGGCATTCCATGCCAAGGGTGCAGGACCTTTGAGCCTGAAGCCACTATTTAGTGCTTCAGGGGTTAAGGGCAAAAGAGTCACTGCATTATCTGCAAAAGAAGAAGGTGGCATCTCT

1_12 GGTTTCATCCAATCAATCATTCACGACAAACCAGAGATGGATTCACCGTTTTTGGTGCTTGATCTTGGGGTCGTCATGGACCTCATGGACAATTGGACCAACAACCTTCCCACAGTTCAACCTTTCTATGCAGTTAAGTGCAACCCTAACCCATGCTTGCTGGGAGCACTGGCAGCACTC

2_11 GGTTTCATCCAATCAATCATTCACGACAAACCAGAGATGGATTCACCGTTTTTGGTGCTTGATCTTGGGGTCGTCATGGACCTCATGGACAATTGGACCAACAACCTTCCCACAGTTCAACCTTTCTATGCAGTTAAGTGCAACCCTAACCCATGCTTGCTGGGAGCACTGGCAGCACTC

3_5 GGTTTCATCCAATCAATCATTCACGACAAACCAGAGATGGATTCACCGTTTTTGGTGCTTGATCTTGGGGTCGTCATGGACCTCATGGACAATTGGACCAACAACCTTCCCACAGTTCAACCTTTCTATGCAGTTAAGTGCAACCCTAACCCATGCTTGCTGGGAGCACTGGCAGCACTC

4_15 GGTTTCATCCAATCAATCATTCACGACAAACCAGAGATGGATTCACCGTTTTTGGTGCTTGATCTTGGGGTCGTCATGGACCTCATGGACAATTGGACCAACAACCTTCCCACAGTTCAACCTTTCTATGCAGTTAAGTGCAACCCTAACCCATGCTTGCTGGGAGCACTGGCAGCACTC

5_13 GGTTTCATCCAATCAATCATTCACGACAAACCAGAGATGGATTCACCGTTTTTGGTGCTTGATCTTGGGGTCGTCATGGACCTCATGGACAATTGGACCAACAACCTTCCCACAGTTCAACCTTTCTATGCAGTTAAGTGCAACCCTAACCCATGCTTGCTGGGAGCACTGGCAGCACTC

6_16 GGTTTCATCCAATCAATCATTCACGACAAACCAGAGATGGATTCACCGTTTTTGGTGCTTGATCTTGGGGTCGTCATGGACCTCATGGACAATTGGACCAACAACCTTCCCACAGTTCAACCTTTCTATGCAGTTAAGTGCAACCCTAACCCATGCTTGCTGGGAGCACTGGCAGCACTC

7_10 GGTTTCATCCAATCAATCATTCACGACAAACCAGAGATGGATTCACCGTTTTTGGTGCTTGATCTTGGGGTCGTCATGGACCTCATGGACAATTGGACCAACAACCTTCCCACAGTTCAACCTTTCTATGCAGTTAAGTGCAACCCTAACCCATGCTTGCTGGGAGCACTGGCAGCACTC

8_11 GGTTTCATCCAATCAATCATTCACGACAAACCAGAGATGGATTCACCGTTTTTGGTGCTTGATCTTGGGGTCGTCATGGACCTCATGGACAATTGGACCAACAACCTTCCCACAGTTCAACCTTTCTATGCAGTTAAGTGCAACCCTAACCCATGCTTGCTGGGAGCACTGGCAGCACTC

9_8 GGTTTCATCCAATCAATCATTCACGACAAACCAGAGATGGATTCACCGTTTTTGGTGCTTGATCTTGGGGTCGTCATGGACCTCATGGACAATTGGACCAACAACCTTCCCACAGTTCAACCTTTCTATGCAGTTAAGTGCAACCCTAACCCATGCTTGCTGGGAGCACTGGCAGCACTC

10_4 GGTTTCATCCAATCAATCATTCACGACAAACCAGAGATGGATTCACCGTTTTTGGTGCTTGATCTTGGGGTCGTCATGGACCTCATGGACAATTGGACCAACAACCTTCCCACAGTTCAACCTTTCTATGCAGTTAAGTGCAACCCTAACCCATGCTTGCTGGGAGCACTGGCAGCACTC

**381 414 459**

**| | |**

1_12 GGTTCCAGCTTCGACTGCGCTAGTCGAGCTGAGATCGAATCCGTTTTGTCACTGGGAGTCTCACCGGACAAAATCATCTACGCCAATCCATGCAAATCCGAGTCTCACATCAAATACGCTGCCAGTGTGGGTGTCAACGTTACAACGTTTGACTCCAAAGAAGAGATCGACAAGATTCGA

2_11 GGTTCCAGCTTCGACTGCGCTAGTCGAGCTGAGATCGAATCCGTTTTGTCACTGGGAGTCTCACCGGACAAAATCATCTACGCCAATCCATGCAAATCCGAGTCTCACATCAAATACGCTGCCAGTGTGGGTGTCAACGTTACAACGTTTGACTCCAAAGAAGAGATCGACAAGATTCGA

3_5 GGTTCCAGCTTCGACTGCGCTAGTCGAGCTGAGATCGAATCCGTTTTGTCACTGGGAGTCTCACCGGACAAAATCATCTACGCCAATCCATGCAAATCCGAGTCTCACATCAAATACGCTGCCAGTGTGGGTGTCAACGTTACAACGTTTGACTCCAAAGAAGAGATCGACAAGATTCGA

4_15 GGTTCCAGCTTCGACTGCGCCAGTCGAGCTGAGATCGAATCCGTTTTGTCACTTGGAGTCTCACCGGACAAAATCATCTACGCCAATCCATGCAAATCMGAGTCTCACATCAAATACGCTGCCAGTGTGGGTGTCAACGTTACAACGTTTGACTCCAAAGAAGAGATCGACAAGATTCGA

5_13 GGTTCCAGCTTCGACTGCGCCAGTCGAGCTGAGATCGAATCCGTTTTGTCACTTGGAGTCTCACCGGACAAAATCATCTACGCCAATCCATGCAAATCCGAGTCTCACATCAAATACGCTGCCAGTGTGGGTGTCAACGTTACAACGTTTGACTCCAAAGAAGAGATCGACAAGATTCGA

6_16 GGTTCCAGCTTCGACTGCGCCAGTCGAGCTGAGATCGAATCCGTTTTGTCACTTGGAGTCTCACCGGACAAAATCATCTACGCCAATCCATGCAAATCAGAGTCTCACATCAAATACGCTGCCAGTGTGGGTGTCAACGTTACAACGTTTGACTCCAAAGAAGAGATCGACAAGATTCGA

7_10 GGTTCCAGCTTCGACTGCGCCAGTCGAGCTGAGATCGAATCCGTTTTGTCACTTGGAGTCTCACCGGACAAAATCATCTACGCCAATCCATGCAAATCAGAGTCTCACATCAAATACGCTGCCAGTGTGGGTGTCAACGTTACAACGTTTGACTCCAAAGAAGAGATCGACAAGATTCGA

8_11 GGTTCCAGCTTCGACTGCGCTAGTCGAGCTGAGATCGAATCCGTTTTGTCACTGGGAGTCTCACCGGACAAAATCATCTACGCCAATCCATGCAAATCCGAGTCTCACATCAAATACGCTGCCAGTGTGGGTGTCAACGTTACAACGTTTGACTCCAAAGAAGAGATCGACAAGATTCGA

9_8 GGTTCCAGCTTCGACTGCGCTAGTCGAGCTGAGATCGAATCCGTTTTGTCACTGGGAGTCTCACCGGACAAAATCATCTACGCCAATCCATGCAAATCCGAGTCTCACATCAAATACGCTGCCAGTGTGGGTGTCAACGTTACAACGTTTGACTCCAAAGAAGAGATCGACAAGATTCGA

10_4 GGTTCCAGCTTCGACTGCGCYAGTCGAGCTGAGATCGAATCCGTTTTGTCACTKGGAGTCTCACCGGACAAAATCATCTACGCCAATCCATGCAAATCCGAGTCTCACATCAAATACGCTGCCAGTGTGGGTGTCAACGTTACAACGTTTGACTCCAAAGAAGAGATCGACAAGATTCGA

558 688

| |

1_12 AAATGGCACCCGAAATGTGAGCTTCTCATCCGCATCAAACCCCCAGGAGACAGCGGAGCACGAAATGCGTTGGGCCTCAAATACGGTGCGCTTCCTGAAGAAGTCATGCCTCTCCTCCAAGCTGCTCAAAACGCGGGGTTGAAGGTCACCGGCGTGTCGTTTCATATCGGAAGTGGAGGA

2_11 AAATGGCACCCGAAATGTGAGCTTCTCATCCGCATCAAACCCCCAGGAGACAGCGGAGCACGAAATGCGTTGGGCCTCAAATACGGTGCGCTTCCTGAAGAAGTCATGCCTCTCCTCCAAGCTGCTCAAAACGCGGGGTTGAAGGTCACCGGCGTGTCGTTTCATATCGGAAGTGGAGGA

3_5 AAATGGCACCCGAAATGTGAGCTTCTCATCCGCATCAAACCCCCAGGAGACAGCGGAGCACGAAATGCGTTGGGCCTCAAATACGGTGCGCTTCCTGAAGAAGTCATGCCTCTCCTCCAAGCTGCTCAAAACGCGGGGTTGAAGGTCACCGGCGTGTCGTTTCATATCGGAAGTGGAGGA

4_15 AAATGGCACCCGAAATGTGAGCTTCTCATCCGCATCAAACCCCCAGGAGACAGCGGAGCACGAAATGCGTTGGGCCTCAAATACGGTGCGCTTCCTGAAGAAGTCATGCCTCTCCTCCAAGCTGCTCAAAACGCGGGGTTGAAGGTCACCGGCGTGTCGTTTCATATCGGAAGTGGAGGA

5_13 AAATGGCACCCGAAATGCGAGCTTCTCATCCGCATCAAACCCCCAGGAGACAGCGGAGCACGAAATGCGTTGGGCCTCAAATACGGTGCGCTTCCTGAAGAAGTCATGCCTCTCCTCCAAGCTGCTCAAAACGCGGGGTTGAAGGTCACCGGCGTGTCGTTTCATATCGGAAGTGGAGGA

6_16 AAATGGCACCCGAAATGTGAGCTTCTCATCCGCATCAAACCCCCAGGAGACAGCGGAGCACGAAATGCGTTGGGCCTCAAATACGGTGCGCTTCCTGAAGAAGTCATGCCTCTCCTCCAAGCTGCTCAAAACGCGGGGTTGAAGGTCACCGGCGTGTCGTTTCATATCGGAAGTGGAGGA

7_10 AAATGGCACCCGAAATGTGAGCTTCTCATCCGCATCAAACCCCCAGGAGACAGCGGAGCACGAAATGCGTTGGGCCTCAAATACGGTGCGCTTCCTGAAGAAGTCATGCCTCTCCTCCAAGCTGCTCAAAACGCGGGGTTGAAGGTCACCGGCGTGTCGTTTCATATCGGAAGTGGAGGA

8_11 AAATGGCACCCGAAATGTGAGCTTCTCATCCGCATCAAACCCCCAGGAGACAGCGGAGCACGAAATGCGTTGGGCCTCAAATACGGTGCGCTTCCTGAAGAAGTCATGCCTCTCCTCCAAGCTGCTCAAAACGCGGGGTTGAAGGTCACCGGCGTGTCGTTTCATATCGGAAGTGGAGGA

9_8 AAATGGCACCCGAAATGTGAGCTTCTCATCCGCATCAAACCCCCAGGAGACAGCGGAGCACGAAATGCGTTGGGCCTCAAATACGGTGCGCTTCCTGAAGAAGTCATGCCTCTCCTCCAAGCTGCTCAAAACGCGGGGTTGAAGGTCACCGGCGTGTCGTTTCATATCGGAAGTGGAGGA

10_4 AAATGGCACCCGAAATGTGAGCTTCTCATCCGCATCAAACCCCCAGGAGACAGCGGAGCACGAAATGCGTTGGGCCTCAAATACGGTGCGCTTCCTGAAGAAGTCATGCCTCTCCTCCAAGCTGCTCAAAACGCGGGGTTGAAGGTCWCCGGCGTGTCGTTTCATATCGGAAGTGGAGGA

**FIGURE S7A (continued)**

776 887

| |

1_12 GCTGATTCTCAAACCTATCACGGAGCTATCGCTGCTGCTAAGAGAGTTTTCGACATGGCTTCTTCTGAGCTAAACATGCCTAGAATGAAAGTACTGGACATTGGTGGCGGTTTCACATGTGGGAAGCAGTTTGAGGCTGCTGCATTGCACGTGAACGAGGCTCTTCAAGTTCACTTCGGA

2_11 GCTGATTCTCAAACCTATCACGGAGCTATCGCTGCTGCTAAGAGAGTTTTCGACATGGCTTCTTCTGAGCTAAACATGCCTAGAATGAAAGTACTGGACATTGGTGGCGGTTTCACATGTGGGAAGCAGTTTGAGGCTGCTGCATTGCACGTGAACGAGGCTCTTCAAGTTCACTTCGGA

3_5 GCTGATTCTCAAACCTATCACGGAGCTATCGCTGCTGCTAAGAGAGTTTTCGACATGGCTTCTTCTGAGCTAAACATGCCTAGAATGAAAGTACTGGACATTGGTGGCGGTTTCACATGTGGGAAGCAGTTTGAGGCTGCTGCATTGCACGTGAACGAGGCTCTTCAAGTTCACTTCGGA

4_15 GCTGATTCTCAAACCTATCACGGAGCTATCGCTGCTGCTAAGAGAGTTTTCGACATGGCTTCTTCTGAGCTAAACATGCCTAGAATGAAAGTACTGGACATTGGTGGCGGTTTCACATGTGGGAAGCAGTTTGAGGCTGCTGCATTGCACGTGAACGAGGCTCTTCAAGTTCACTTCGGA

5_13 GCTGATTCTCAAACCTATCACGGAGCTATCGCTGCTGCTAAGAGAGTTTTCGACATGGCTTCTTCTGAGCTAAACATGCCTAGAATGAAAGTACTGGACATTGGTGGCGGTTTCACATGTGGGAAGCAGTTTGAGGCTGCTGCATTGCACGTGAACGAGGCTCTTCAAGTTCACTTCGGA

6_16 GCTGATTCTCAAACCTATCACGGAGCTATCGCTGCTGCTAAGAGAGTTTTCGACATGGCTTCTTCTGAGCTAAACATGCCTAGAATGAAAGTACTGGACATTGGTGGCGGTTTCACATGTGGGAAGCAGTTTGAGGCTGCTGCATTGCACGTGAACGAGGCTCTTCAAGTTCACTTCGGA

7_10 GCTGATTCTCAAACCTATCACGGAGCTATCGCTGCTGCTAAGAGAGTTTTCGACAYGGCTTCTTCTGAGCTAAACATGCCTAGAATGAAAGTACTGGACATTGGTGGCGGTTTCACATGTGGGAAGCAGTTTGAGGCTGCTGCATTGCACGTGAACGAGGCTCTTCRAGTTCACTTCGGA

8_11 GCTGATTCTCAAACCTATCACGGAGCTATCGCTGCTGCTAAGAGAGTTTTCGACATGGCTTCTTCTGAGCTAAACATGCCTAGAATGAAAGTACTGGACATTGGTGGCGGTTTCACATGTGGGAAGCAGTTTGAGGCTGCTGCATTGCACGTGAACGAGGCTCTTCAAGTTCACTTCGGA

9_8 GCTGATTCTCAAACCTATCACGGAGCTATCGCTGCTGCTAAGAGAGTTTTCGACATGGCTTCTTCTGAGCTAAACATGCCTAGAATGAAAGTACTGGACATTGGTGGCGGTTTCACATGTGGGAAGCAGTTTGAGGCTGCTGCATTGCACGTGAACGAGGCTCTTCAAGTTCACTTCGGA

10_4 GCTGATTCTCAAACCTATCACGGAGCTATCGCTGCTGCTAAGAGAGTTTTCGACATGGCTTCTTCTGAGCTAAACATGCCTAGAATGAAAGTACTGGACATTGGTGGCGGTTTCACATGTGGGAAGCAGTTTGAGGCTGCTGCATTGCACGTGAACGAGGCTCTTCAAGTTCACTTCGGA

Met/Thr Gln/Arg

1_12 GATGAAGAGGGTGTCGTGGTTATTGGAGAACCGGGTCGTTATTTTGCTGAGTCAGCTTTTACGTTGGCCAGTAAAGTTATTGGGAAGCGTGTAAGAGGCGAGGTGAGGGAGTATTGGATCGACGATGGGATCTACGGTTCCCTGAATTGCATAATGTTTGATTTCGCGACAGTCACGTGC

2_11 GATGAAGAGGGTGTCGTGGTTATTGGAGAACCGGGTCGTTATTTTGCTGAGTCAGCTTTTACGTTGGCCAGTAAAGTTATTGGGAAGCGTGTAAGAGGCGAGGTGAGGGAGTATTGGATCGACGATGGGATCTACGGTTCCCTGAATTGCATAATGTTTGATTTCGCGACAGTCACGTGC

3_5 GATGAAGAGGGTGTCGTGGTTATTGGAGAACCGGGTCGTTATTTTGCTGAGTCAGCTTTTACGTTGGCCAGTAAAGTTATTGGGAAGCGTGTAAGAGGCGAGGTGAGGGAGTATTGGATCGACGATGGGATCTACGGTTCCCTGAATTGCATAATGTTTGATTTCGCGACAGTCACGTGC

4_15 GATGAAGAGGGTGTCGTGGTTATTGGAGAACCGGGTCGTTATTTTGCTGAGTCAGCTTTTACGTTGGCCAGTAAAGTTATTGGGAAGCGTGTAAGAGGCGAGGTGAGGGAGTATTGGATCGACGATGGGATCTACGGTTCCCTGAATTGCATAATGTTTGATTTCGCGACAGTCACGTGC

5_13 GATGAAGAGGGTGTCGTGGTTATTGGAGAACCGGGTCGTTATTTTGCTGAGTCAGCTTTTACGTTGGCCAGTAAAGTTATTGGGAAGCGTGTAAGAGGCGAGGTGAGGGAGTATTGGATCGACGATGGGATCTACGGTTCCCTGAATTGCATAATGTTTGATTTCGCGACAGTCACGTGC

6_16 GATGAAGAGGGTGTCGTGGTTATTGGAGAACCGGGTCGTTATTTTGCTGAGTCAGCTTTTACGTTGGCCAGTAAAGTTATTGGGAAGCGTGTAAGAGGCGAGGTGAGGGAGTATTGGATCGACGATGGGATCTACGGTTCCCTGAATTGCATAATGTTTGATTTCGCGACAGTCACGTGC

7_10 GATGAAGAGGGTGTCGTGGTTATTGGAGAACCGGGTCGTTATTTTGCTGAGTCAGCTTTTACGTTGGCCAGTAAAGTTATTGGGAAGCGTGTAAGAGGCGAGGTGAGGGAGTATTGGATCGACGATGGGATCTACGGTTCCCTGAATTGCATAATGTTTGATTTCGCGACAGTCACGTGC

8_11 GATGAAGAGGGTGTCGTGGTTATTGGAGAACCGGGTCGTTATTTTGCTGAGTCAGCTTTTACGTTGGCCAGTAAAGTTATTGGGAAGCGTGTAAGAGGCGAGGTGAGGGAGTATTGGATCGACGATGGGATCTACGGTTCCCTGAATTGCATAATGTTTGATTTCGCGACAGTCACGTGC

9_8 GATGAAGAGGGTGTCGTGGTTATTGGAGAACCGGGTCGTTATTTTGCTGAGTCAGCTTTTACGTTGGCCAGTAAAGTTATTGGGAAGCGTGTAAGAGGCGAGGTGAGGGAGTATTGGATCGACGATGGGATCTACGGTTCCCTGAATTGCATAATGTTTGATTTCGCGACAGTCACGTGC

10_4 GATGAAGAGGGTGTCGTGGTTATTGGAGAACCGGGTCGTTATTTTGCTGAGTCAGCTTTTACGTTGGCCAGTAAAGTTATTGGGAAGCGTGTAAGAGGCGAGGTGAGGGAGTATTGGATCGACGATGGGATCTACGGTTCCCTGAATTGCATAATGTTTGATTTCGCGACAGTCACGTGC

**1167 1173 1214**

**| | |**

1_12 TCACCACTCGCGTGCAGCTCAAAGCCTGAGAATCCCAGATGCAGAGACTCGAAAACGTACCCTTCAACTGTGTTTGGTCCCACTTGCGATTCATTAGATACTATTTTCAGAGATTACCAGCTCCCGGAACTGGGACTTAACGATTGGCTTGTCTTCCCGAATATGGGTGCTTATACGACG

2_11 TCACCACTCGCGTGCAGCTCAAAGCCTGAGAATCCCAGATGCAGAGACTCGAAAACGTACCCTTCAACTGTGTTTGGTCCCACTTGCGATTCATTAGATACTATTTTCAGAGATTACCAGCTCCCGGAACTGGGACTTAACGATTGGCTTGTCTTCCCGAATATGGGTGCTTATACGACG

3_5 TCACCACTCGCGTGCAGCTCAAAGCCTGAGAATCCCAGATGCAGAGACTCGAAAACGTACCCTTCAACTGTGTTTGGTCCCACTTGCGATTCATTAGATACTATTTTCAGAGATTACCAGCTCCCGGAACTGGGACTTAACGATTGGCTTGTCTTCCCGAATATGGGTGCTTATACGACG

4_15 TCACCACTCGCGTGCAGCTCAAAGCCTGAGAATCCCAGATGCAGAGACTCGAAAACGTACCCTTCAACTGTGTTTGGTCCCACTTGYGATTCRTTAGATACTATTTTCAGAGATTACCAGCTCCCGGAACTGGRACTTAACGATTGGCTTGTCTTCCCGAATATGGGTGCTTATACGACG

5_13 TCACCACTCGCGTGCAGCTCAAAGCCTGAGAATCCCAGATGCAGAGACTCGAAAACGTACCCTTCAACTGTGTTTGGTCCCACTTGCGATTCATTAGATACTATTTTCAGAGATTACCAGCTCCCGGAACTGGGACTTAACGATTGGCTTGTCTTCCCGAATATGGGTGCTTATACGACG

6_16 TCACCACTCGCGTGCAGCTCAAAGCCTGAGAATCCCAGATGCAGAGACTCGAAAACGTACCCTTCAACTGTGTTTGGTCCCACTTGCGATTCATTAGATACTATTTTCAGAGATTACCAGCTCCCGGAACTGGGACTTAACGATTGGCTTGTCTTCCCGAATATGGGTGCTTATACGACG

7_10 TCACCACTCGCGTGCAGCTCAAAGCCTGAGAATCCCAGATGCAGAGACTCGAAAACGTACCCTTCAACTGTGTTTGGTCCCACTTGCGATTCATTAGATACTATTTTCAGAGATTACCAGCTCCCGGAACTGGGACTTAACGATTGGCTTGTCTTCCCGAATATGGGTGCTTATACGACG

8_11 TCACCACTCGCGTGCAGCTCAAAGCCTGAGAATCCCAGATGCAGAGACTCGAAAACGTACCCTTCAACTGTGTTTGGTCCCACTTGCGATTCATTAGATACTATTTTCAGAGATTACCAGCTCCCGGAACTGGGACTTAACGATTGGCTTGTCTTCCCGAATATGGGTGCTTATACGACG

9_8 TCACCACTCGCGTGCAGCTCAAAGCCTGAGAATCCCAGATGCAGAGACTCGAAAACGTACCCTTCAACTGTGTTTGGTCCCACTTGTGATTCGTTAGATACTATTTTCAGAGATTACCAGCTCCCGGAACTGGAACTTAACGATTGGCTTGTCTTCCCGAATATGGGTGCTTATACGACG

10_4 TCACCACTCGCGTGCAGCTCAAAGCCTGAGAATCCCAGATGCAGAGACTCGAAAACGTACCCTTCAACTGTGTTTGGTCCCACTTGCGATTCATTAGATACTATTTTCAGAGATTACCAGCTCCCGGAACTGGGACTTAACGATTGGCTTGTCTTCCCGAATATGGGTGCTTATACGACG

Glu/Gly

1334 1341 1364

| | |

1_12 TCGTCAGGGACCAACTTCAATGGCTTTAGCACTTCAGCTATCGCTACCTACCTTGCATGTTCCAGTCCCATTGCGCGGGAACAAGCTATGATCGAATCAGCTGCGATGTTCGCTAATAGTATGTTTAGTAGTTTTGCAACACCTAAACCAATAGTTTGAgagaaag

2_11 TCGTCAGGGACCAACTTCAATGGCTTTAGCACTTCAGCTATCGCTACCTACCTTGCATGTTCCAGTCCCATTGCGCGGGAACAAGCTATGATCGAATCAGCTGCGATGTTCGCTAATAGTATGTTTAGTAGTTTTGCAACACCTAAACCAATAGTTTGAgagaaag

3_5 TCGTCAGGGACCAACTTCAATGGCTTTAGCACTTCAGCTATCGCTACCTACCTTGCATGTTCCAGTCCCATTGCGCGGGAACAAGCTATGATCGAATCAGCTGCGATGTTCGCTAATAGTATGTTTAGTAGTTTTGCAACACCTAAACCAATAGTTTGAgagaaag

4_15 TCGTCAGGGACCAACTTCAATGGCTTTAGCACTTCAGCTATCGCTACCTACCTTGCATGTTCCAGTCCCATTGGGCGGGAACAAGCTATGATCGAATCAGCTGTGATGTTCGCTAATAGTATGTTTAGTAGTTTTGCAACACCTAAACCAATAGTTTGAgagaaag

5_13 TCGTCAGGGACCAACTTCAATGGCTTTAGCACTTCAGCTATCGCTACCTACCTTGCATGTTCCAGTCCCATTGCGCGGGAACAAGCTATGATCGAATCAGCTGCGATGTTCGCTAATAGTATGTTTAGTAGTTTTGCAACACCTAAACCAATAGTTTGAgagaaag

6_16 TCGTCAGGGACCAACTTCAATGGCTTTAGCACTTCAGCTATCGCTACCTACCTTGCATGTTCCAGTCCCATTGCGCGGGAGCAAGCTATGATCGAATCAGCTGCGATGTTCGCTAATAGTATGTTTAGTAGTTTTGCAACACCTAAACCAATAGTTTGAgagaaag

7_10 TCGTCAGGGACCAACTTCAATGGCTTTAGCACTTCAGCTATCGCTACCTACCTTGCATGTTCCAGTCCCATTGCGCGGGARCAAGCTATGATCGAATCAGCTGCGATGTTCGCTAATAGTATGTTTAGTAGTTTTGCAACACCTAAACCAATAGTTTGAgagaaag

8_11 TCGTCAGGGACCAACTTCAATGGCTTTAGCACTTCAGCTATCGCTACCTACCTTGCATGTTCCAGTCCCATTGCGCGGGAACAAGCTATGATCGAATCAGCTGCGATGTTCGCTAATAGTATGTTTAGTAGTTTTGCAACACCTAAACCAATAGTTTGAgagaaag

9_8 TCGTCAGGGACCAACTTCAATGGCTTTAGCACTTCAGCTATCGCTACCTACCTTGCATGTTCCAGTCCCATTGGGCGGGAACAAGCTATGATCGAATCAGCTGTGATGTTCGCTAATAGTATGTTTAGTAGTTTTGCAACACCTAAACCAATAGTTTGAgagaaag

10_4 TCGTCAGGGACCAACTTCAATGGCTTTAGCACTTCAGCTATCGCTACCTACCTTGCATGTTCCAGTCCCATTGCGCGGGARCAAGCTATGATCGAATCAGCTGCGATGTTCGCTAATAGTATGTTTAGTAGTTTTGCAACACCTAAACCAATAGTTTGAgagaaag

Gly/Ala Val/Ala

**FIGURE S7B |** SfLDC predicted proteins (from DNA sequences in **Figure S7A**). X indicates a heterozygous sample, therefore it is likely that two different proteins are produced. ▼ active site residue, and F (340) critical for L/ODC dual-activity, based on Bunsupa *et al*. (2012).

1 180

| ▼ |

1_12 MPTLVTEAFHAKGAGPLSLKPLFSASGVKGKRVTALSAKEEGGISGFIQSIIHDKPEMDSPFLVLDLGVVMDLMDNWTNNLPTVQPFYAVKCNPNPCLLGALAALGSSFDCASRAEIESVLSLGVSPDKIIYANPCKSESHIKYAASVGVNVTTFDSKEEIDKIRKWHPKCELLIRIKPP

2_11 MPTLVTEAFHAKGAGPLSLKPLFSASGVKGKRVTALSAKEEGGISGFIQSIIHDKPEMDSPFLVLDLGVVMDLMDNWTNNLPTVQPFYAVKCNPNPCLLGALAALGSSFDCASRAEIESVLSLGVSPDKIIYANPCKSESHIKYAASVGVNVTTFDSKEEIDKIRKWHPKCELLIRIKPP

3_5 MPTLVTEAFHAKGAGPLSLKPLFSASGVKGKRVTALSAKEEGGISGFIQSIIHDKPEMDSPFLVLDLGVVMDLMDNWTNNLPTVQPFYAVKCNPNPCLLGALAALGSSFDCASRAEIESVLSLGVSPDKIIYANPCKSESHIKYAASVGVNVTTFDSKEEIDKIRKWHPKCELLIRIKPP

4_15 MPTLVTEAFHAKGAGPLSLKPLFSASGVKGKRVTALSAKEEGGISGFIQSIIHDKPEMDSPFLVLDLGVVMDLMDNWTNNLPTVQPFYAVKCNPNPCLLGALAALGSSFDCASRAEIESVLSLGVSPDKIIYANPCKSESHIKYAASVGVNVTTFDSKEEIDKIRKWHPKCELLIRIKPP

5_13 MPTLVTEAFHAKGAGPLSLKPLFSASGVKGKRVTALSAKEEGGISGFIQSIIHDKPEMDSPFLVLDLGVVMDLMDNWTNNLPTVQPFYAVKCNPNPCLLGALAALGSSFDCASRAEIESVLSLGVSPDKIIYANPCKSESHIKYAASVGVNVTTFDSKEEIDKIRKWHPKCELLIRIKPP

6_16 MPTLVTEAFHAKGAGPLSLKPLFSASGVKGKRVTALSAKEEGGISGFIQSIIHDKPEMDSPFLVLDLGVVMDLMDNWTNNLPTVQPFYAVKCNPNPCLLGALAALGSSFDCASRAEIESVLSLGVSPDKIIYANPCKSESHIKYAASVGVNVTTFDSKEEIDKIRKWHPKCELLIRIKPP

7_10 MPTLVTEAFHAKGAGPLSLKPLFSASGVKGKRVTALSAKEEGGISGFIQSIIHDKPEMDSPFLVLDLGVVMDLMDNWTNNLPTVQPFYAVKCNPNPCLLGALAALGSSFDCASRAEIESVLSLGVSPDKIIYANPCKSESHIKYAASVGVNVTTFDSKEEIDKIRKWHPKCELLIRIKPP

8_11 MPTLVTEAFHAKGAGPLSLKPLFSASGVKGKRVTALSAKEEGGISGFIQSIIHDKPEMDSPFLVLDLGVVMDLMDNWTNNLPTVQPFYAVKCNPNPCLLGALAALGSSFDCASRAEIESVLSLGVSPDKIIYANPCKSESHIKYAASVGVNVTTFDSKEEIDKIRKWHPKCELLIRIKPP

9_8 MPTLVTEAFHAKGAGPLSLKPLFSASGVKGKRVTALSAKEEGGISGFIQSIIHDKPEMDSPFLVLDLGVVMDLMDNWTNNLPTVQPFYAVKCNPNPCLLGALAALGSSFDCASRAEIESVLSLGVSPDKIIYANPCKSESHIKYAASVGVNVTTFDSKEEIDKIRKWHPKCELLIRIKPP

10_4 MPTLVTEAFHAKGAGPLSLKPLFSASGVKGKRVTALSAKEEGGISGFIQSIIHDKPEMDSPFLVLDLGVVMDLMDNWTNNLPTVQPFYAVKCNPNPCLLGALAALGSSFDCASRAEIESVLSLGVSPDKIIYANPCKSESHIKYAASVGVNVTTFDSKEEIDKIRKWHPKCELLIRIKPP

************************************************************************************************************************************************************************************

340 360

▼ ▼ ▼▼▼ ▼ ▼▼ ▼▼| |

1_12 GDSGARNALGLKYGALPEEVMPLLQAAQNAGLKVTGVSFHIGSGGADSQTYHGAIAAAKRVFDMASSELNMPRMKVLDIGGGFTCGKQFEAAALHVNEALQVHFGDEEGVVVIGEPGRYFAESAFTLASKVIGKRVRGEVREYWIDDGIYGSLNCIMFDFATVTCSPLACSSKPENPRCR

2_11 GDSGARNALGLKYGALPEEVMPLLQAAQNAGLKVTGVSFHIGSGGADSQTYHGAIAAAKRVFDMASSELNMPRMKVLDIGGGFTCGKQFEAAALHVNEALQVHFGDEEGVVVIGEPGRYFAESAFTLASKVIGKRVRGEVREYWIDDGIYGSLNCIMFDFATVTCSPLACSSKPENPRCR

3_5 GDSGARNALGLKYGALPEEVMPLLQAAQNAGLKVTGVSFHIGSGGADSQTYHGAIAAAKRVFDMASSELNMPRMKVLDIGGGFTCGKQFEAAALHVNEALQVHFGDEEGVVVIGEPGRYFAESAFTLASKVIGKRVRGEVREYWIDDGIYGSLNCIMFDFATVTCSPLACSSKPENPRCR

4_15 GDSGARNALGLKYGALPEEVMPLLQAAQNAGLKVTGVSFHIGSGGADSQTYHGAIAAAKRVFDMASSELNMPRMKVLDIGGGFTCGKQFEAAALHVNEALQVHFGDEEGVVVIGEPGRYFAESAFTLASKVIGKRVRGEVREYWIDDGIYGSLNCIMFDFATVTCSPLACSSKPENPRCR

5_13 GDSGARNALGLKYGALPEEVMPLLQAAQNAGLKVTGVSFHIGSGGADSQTYHGAIAAAKRVFDMASSELNMPRMKVLDIGGGFTCGKQFEAAALHVNEALQVHFGDEEGVVVIGEPGRYFAESAFTLASKVIGKRVRGEVREYWIDDGIYGSLNCIMFDFATVTCSPLACSSKPENPRCR

6_16 GDSGARNALGLKYGALPEEVMPLLQAAQNAGLKVTGVSFHIGSGGADSQTYHGAIAAAKRVFDMASSELNMPRMKVLDIGGGFTCGKQFEAAALHVNEALQVHFGDEEGVVVIGEPGRYFAESAFTLASKVIGKRVRGEVREYWIDDGIYGSLNCIMFDFATVTCSPLACSSKPENPRCR

7_10 GDSGARNALGLKYGALPEEVMPLLQAAQNAGLKVTGVSFHIGSGGADSQTYHGAIAAAKRVFDXASSELNMPRMKVLDIGGGFTCGKQFEAAALHVNEALXVHFGDEEGVVVIGEPGRYFAESAFTLASKVIGKRVRGEVREYWIDDGIYGSLNCIMFDFATVTCSPLACSSKPENPRCR

8_11 GDSGARNALGLKYGALPEEVMPLLQAAQNAGLKVTGVSFHIGSGGADSQTYHGAIAAAKRVFDMASSELNMPRMKVLDIGGGFTCGKQFEAAALHVNEALQVHFGDEEGVVVIGEPGRYFAESAFTLASKVIGKRVRGEVREYWIDDGIYGSLNCIMFDFATVTCSPLACSSKPENPRCR

9_8 GDSGARNALGLKYGALPEEVMPLLQAAQNAGLKVTGVSFHIGSGGADSQTYHGAIAAAKRVFDMASSELNMPRMKVLDIGGGFTCGKQFEAAALHVNDALQVHFGDEEGVVVIGEPGRYFAESAFTLASKVIGKRVRGEVREYWIDDGIYGSLNCIMFDFATVTCSPLACSSKPENPRCR

10_4 GDSGARNALGLKYGALPEEVMPLLQAAQNAGLKVXGVSFHIGSGGADSQTYHGAIAAAKRVFDMASSELNMPRMKVLDIGGGFTCGKQFEAAALHVNEALQVHFGDEEGVVVIGEPGRYFAESAFTLASKVIGKRVRGEVREYWIDDGIYGSLNCIMFDFATVTCSPLACSSKPENPRCR

**********************************:**************************** ************************************.*******************************************************************************

Thr/Ser Met/Thr Gln/Arg

458

▼▼ ▼ |

1_12 DSKTYPSTVFGPTCDSLDTIFRDYQLPELGLNDWLVFPNMGAYTTSSGTNFNGFSTSAIATYLACSSPIAREQAMIESAAMFANSMFSSFATPKPIV--

2_11 DSKTYPSTVFGPTCDSLDTIFRDYQLPELGLNDWLVFPNMGAYTTSSGTNFNGFSTSAIATYLACSSPIAREQAMIESAAMFANSMFSSFATPKPIV--

3_5 DSKTYPSTVFGPTCDSLDTIFRDYQLPELGLNDWLVFPNMGAYTTSSGTNFNGFSTSAIATYLACSSPIAREQAMIESAAMFANSMFSSFATPKPIV--

4_15 DSKTYPSTVFGPTCDSLDTIFRDYQLPELXLNDWLVFPNMGAYTTSSGTNFNGFSTSAIATYLACSSPIGREQAMIESAVMFANSMFSSFATPKPIV--

5_13 DSKTYPSTVFGPTCDSLDTIFRDYQLPELGLNDWLVFPNMGAYTTSSGTNFNGFSTSAIATYLACSSPIAREQAMIESAAMFANSMFSSFATPKPIV--

6_16 DSKTYPSTVFGPTCDSLDTIFRDYQLPELGLNDWLVFPNMGAYTTSSGTNFNGFSTSAIATYLACSSPIAREQAMIESAAMFANSMFSSFATPKPIVEK

7_10 DSKTYPSTVFGPTCDSLDTIFRDYQLPELGLNDWLVFPNMGAYTTSSGTNFNGFSTSAIATYLACSSPIAREQAMIESAAMFANSMFSSFATPKPIV--

8_11 DSKTYPSTVFGPTCDSLDTIFRDYQLPELGLNDWLVFPNMGAYTTSSGTNFNGFSTSAIATYLACSSPIAREQAMIESAAMFANSMFSSFATPKPIV--

9_8 DSKTYPSTVFGPTCDSLDTIFRDYQLPELELNDWLVFPNMGAYTTSSGTNFNGFSTSAIATYLACSSPIGREQAMIESAVMFANSMFSSFATPKPIV--

10_4 DSKTYPSTVFGPTCDSLDTIFRDYQLPELGLNDWLVFPNMGAYTTSSGTNFNGFSTSAIATYLACSSPIAREQAMIESAAMFANSMFSSFATPKPIV--

***************************** ***************************************.********* *****************

Glu/Gly Gly/Ala Val/Ala

**FIGURE S7C |** *SfCAO* gDNA sequence (partial, 5' UTR to part exon 1 (-53..194)). Each sequence likely represents two genes, *SfCAO1* and *SfCAO2* (see **Figure S7D,E** respectively and **Figure S9**)*.*

81

|

1_12 tggattgctgtgacacgttgtggtggaattagaaagttctctcacgctttataATGGCATCAGTTTCACAAAAGGTGGCGYCACCTTCTCCTTGCTGTTCCCCCGGCGGCGACTCTAATCACATTCCACTCCATGCTGCCGCCACTTCCTCTGCCGAGACTCAAGACTGGACTGATACCA

2_11 tggattgctgtgacacgttgtggtggaattagaaagttctctcacgctttataATGGCATCAGTTTCACAAAAGGTGGCGYCACCTTCTCCTTGCTGTTCCCCCGGCGGCGACTCTAATCACATTCCACTCCATGCTGCCGCCACTTCCTCTGCCGAGACTCAAGACTGGACTGATACCA

3_5 tggattgctgtgacacgttgtggtggaattagaaagttctctcacgctttataATGGCATCAGTTTCACAAAAGGTGGCGYCACCTTCTCCTTGCTGTTCCCCCGGCGGCGACTCTAATCACATTCCACTCCATGCTGCCGCCACTTCCTCTGCCGAGACTCAAGACTGGACTGATACCA

4_15 tggattgctgtgacacgttgtggtggaattagaaagttctctcacgctttataATGGCATCAGTTTCACAAAAGGTGGCGYCACCTTCTCCTTGCTGTTCCCCCGGCGGCGACTCTAATCACATTCCACTCCATGCTGCCGCCACTTCCTCTGCCGAGACTCAAGACTGGACTGATACCA

5_13 tggattgctgtgacacgttgtggtggaattagaaagttctctcacgctttataATGGCATCAGTTTCACAAAAGGTGGCGYCACCTTCTCCTTGCTGTTCCCCCGGCGGCGACTCTAATCACATTCCACTCCATGCTGCCGCCACTTCCTCTGCCGAGACTCAAGACTGGACTGATACCA

6_16 tggattgctgtgacacgttgtggtggaattagaaagttctctcacgctttataATGGCATCAGTTTCACAAAAGGTGGCGYCACCTTCTCCTTGCTGTTCCCCCGGCGGCGACTCTAATCACATTCCACTCCATGCTGCCGCCACTTCCTCTGCCGAGACTCAAGACTGGACTGATACCA

7_10 tggattgctgtgacacgttgtggtggaattagaaagttctctcacgctttataATGGCATCAGTTTCACAAAAGGTGGCGYCACCTTCTCCTTGCTGTTCCCCCGGCGGCGACTCTAATCACATTCCACTCCATGCTGCCGCCACTTCCTCTGCCGAGACTCAAGACTGGACTGATACCA

8_11 tggattgctgtgacacgttgtggtggaattagaaagttctctcacgctttataATGGCATCAGTTTCACAAAAGGTGGCGYCACCTTCTCCTTGCTGTTCCCCCGGCGGCGACTCTAATCACATTCCACTCCATGCTGCCGCCACTTCCTCTGCCGAGACTCAAGACTGGACTGATACCA

9_8 tggattgctgtgacacgttgtggtggaattagaaagttctctcacgctttataATGGCATCAGTTTCACAAAAGGTGGCGYCACCTTCTCCTTGCTGTTCCCCCGGCGGCGACTCTAATCACATTCCACTCCATGCTGCCGCCACTTCCTCTGCCGAGACTCAAGACTGGACTGATACCA

10_4 tggattgctgtgacacgttgtggtggaattagaaagttctctcacgctttataATGGCATCAGTTTCACAAAAGGTGGCGYCACCTTCTCCTTGCTGTTCCCCCGGCGGCGACTCTAATCACATTCCACTCCATGCTGCCGCCACTTCCTCTGCCGAGACTCAAGACTGGACTGATACCA

************************************************************************************************************************************************************************************

Pro/Ser

220

|

1_12 TCTCTGACGACCGCCGCCCCAACACGGTGGCCCTCGTTCGCCCCGTCGACTCCCTTCCTGTGCCTCC

2_11 TCTCTGACGACCGCCGCCCCAACACGGTGGCCCTCGTTCKCCCCGTCGACTCCCTTCCTGTGCCTCC

3_5 TCTCTGACGACCGCCGCCCCAACACGGTGGCCCTCGTTCKCCCCGTCGACTCCCTTCCTGTGCCTCC

4_15 TCTCTGACGACCGCCGCCCCAACACGGTGGCCCTCGTTCKCCCCGTCGACTCCCTTCCTGTGCCTCC

5_13 TCTCTGACGACCGCCGCCCCAACACGGTGGCCCTCGTTCKCCCCGTCGACTCCCTTCCTGTGCCTCC

6_16 TCTCTGACGACCGCCGCCCCAACACGGTGGCCCTCGTTCKCCCCGTCGACTCCCTTCCTGTGCCTCC

7_10 TCTCTGACGACCGCCGCCCCAACACGGTGGCCCTCGTTCKCCCCGTCGACTCCCTTCCTGTGCCTCC

8_11 TCTCTGACGACCGCCGCCCCAACACGGTGGCCCTCGTTCKCCCCGTCGACTCCCTTCCTGTGCCTCC

9_8 TCTCTGACGACCGCCGCCCCAACACGGTGGCCCTCGTTCKCCCCGTCGACTCCCTTCCTGTGCCTCC

10_4 TCTCTGACGACCGCCGCCCCAACACGGTGGCCCTCGTTCKCCCCGTCGACTCCCTTCCTGTGCCTCC

*************************************** ***************************

Arg/Leu

**FIGURE S7D |** *SfCAO1* gDNA sequence (part of exon 10 to 3' UTR).

41 56 63 68 69 75 137

| | | || | |

1_12 AACCGGTCTTTAGAAGAGACTAATATAGTTCTTTGgtgcg--ctctctctctctcttaaaaaagagtgtactgctaatatttttctctataaaatagtttaaaacactgtccaacagtaccaagaaataacctcttctgtaatttatattacattatattaagattagagatgacataaatt

2_11 -ACCGGTCTTTAGAAGAGACTAATATAGTTCTTTGgtgcg--ctctctctctctcttaaaaaagagtgtgttgctartatttttctctataaaatagtttaaaacactgtccaacagtaccaagaaataacctcttctgtaatttatattacattatattaagattagagatgacataaatt

3_5 ----GGTCTTTAGAAGAGACTAATATAGTTCTTTGgtgcgctctctctctctctcttaaaaaagannnnnnnnnnnnnnnnnnnnnnnnnnnnnnnnnnnnnnnnnnnnnnnnnnnnnnnnnnnnnnnnnnnnnnnnnnnnnnnnnnnnnnnnnnnnnnnnnnnnnnnnnnnnacataaatt

5_13 AACCGGTCTTTAGAAGAGACTAATATAGTTCTTTGgtgcg--ctctctctctctcttaaaaaagagtgtactgctaatatttttctctataaaatagtttaaaacactgtccaacagtaccaagaaataacctcttctgtaatttatattacattatattaagattagagatgacataaatt

6_16 -ACCGGTCTTTAGAAGAGACTAATATAGTTCTTTGgtgcg--ctctctctctctcttaaaaaagagtgtgytgctartatttttctctataaaatagtttaaaacactgtccaacagtaccaagaaataacctcttctktaatttatattacattatattaagattagagatgacataaatt

7_10 -ACCGGTCTTTAGAAGAGACTAATATAGTTCTTTGgtgcg--ctctctctctctcttaaaaaagagtgtgytgctartatttttctctataaaatagtttaaaacactgtccaacagtaccaagaaataacctcttctgtaatttatattacattatattaagattagagatgacataaatt

8_11 AACCGGTCTTTAGAAGAGACTAATATAGTTCTTTGgtgcg----ctctctctctcttaaaaaagtgtgtgttgctagtatttttctctataaaatagtttaaaacactgtccnacagtaccaagaaataacctcttctttaatttatattacattatattaagattagagatgacataaatt

9_8 AACCGGTCTTTAGAAGAGACTAATATAGTTCTTTGgtgcg----ctctctctctcttaaaaaagtgtgtgttgctagtatttttctctataaaatagtttaaaacactgtccaacagtaccaagaaataacctcttctttaatttatattacattatattaagattagagatgacataaatt

10_4 -ACCGGTCTTTAGAAGAGACTAATATAGTTCTTTGgtgcg--ctctctctctctcttaaaaaagagtgtgttgctartatttttctctataaaatagtttaaaacactgtccaacagtaccaagaaataacctcttctgtaatttatattacattatattaagattagagatgacataaatt

182 322 335

| | |

1_12 ccccaattccaactttttgttccaatccactttaatgttccacccttaaaatggtttcacattttgtcttctggagactaaccgtgttatcttcagGTATGTATTTGGAGTCACACATGTTCCTCGTTTAGAAGACTGGCCTGTTATGCCAGTAGAGCACATTGGTTTTATGCTCATGgt

2_11 cyccaattccaactttttgttccaatccactttaatgttccacccttaaaatggtttcacattttgtcttctggagactaaccgtgttatcttcagGTATGTATTTGGAGTCACACATNNNNNNNNNNNNNNNNNNNNNNNNNNNNNGCCAGTAGAGCACATTGGTTTTATGCTCATGgt

3_5 ccccaattccaactttttgttccaatccactttaatgttccacccttaaaatggtttcacattttgtcttctggagactaaccgtgttatcttcagGTATGTATTTGGAGTCACACATGTTCCTCGTTTAGAAGACTGGCCTGTTATGCCAGTAAAGCACATTGGTTTTATGCTCATGgt

5_13 ccccaattccaactttttgttccaatccactttaatgttccacccttaaaatggtttcacattttgtcttctggagactaaccgtgttatcttcagGTATGTATTTGGAGTCACACATGTTCCTCGTTTAGAAGACTGGCCTGTTATGCCAGTAGAGCACATTGGTTTTATGCTCATGgt

6_16 ccccaattccaactttttgttccaatccactttaatgttccacccttaaaatggtttcacattttgtcttctggagactaaccgtgttatcttcagGTATGTATTTGGAGTCACACATNNNNNNNNNNNNNNNNACTGGCCTGTTATGCCAGTAGAGCACATTGGTTTTATGCTCATGgt

7_10 cyccaattccaactttttgttccaatccactttaatgttccacccttaaaatggtttcacattttgtcttctggagactaaccgtgttatcttcagGTATGTATTTGGAGTCACACATGTTCCNNNNNNNNNNGACTGGCCTGTTATGCCAGTAGAGCACATTGGTTTTATGCTCATGgt

8_11 ccccaattccaactttttgttccaatccactttaatgttccacccttaaaatggtttcacattttgtcttctggagactaaccgtgttatcttcagGTATGTATTTGGAGTCACACATGTTCCTCGTTTAGAAGACTGGCCCGTTATGCCAGTAGAGCACATTGGTTTTATGCTCATGgt

9_8 ccccaattccaactttttgttccaatccactttaatgttccacccttaaaatggtttcacattttgtcttctggagactaaccgtgttat------------------------------------------------------------------------------------------

10_4 ccccaattccaactttttgttccaatccactttaatgttccacccttaaaatggtttcacattttgtcttctggagactaaccgtgttatcttcagGTATGTATTTGGAGTCACACATGTTCNNNNNNNNNNNGACTGGCCTGTTATGCCAGTAGAGCACATTGGTTTTATGCTCATGgt

Glu/Lys

386 423 458 463 474

| | | | |

1_12 aattacctgattgattgttttcata-ttttttttaccactctttttatgctcatggtaattacctgattgattgttttcatatttattaaacttcatctggagattttccaattttctgatcatgaagctctgttgatgtgtgcagCCTCATGGATTCTTCAATTGTTCCCCTGCGATAG

2_11 aattacctgattgattgttttcata-ttttttttaccactctttttatgctcatggtaattacctgattgattgttttcatatttattaaacttcatcyggagrttttccaattktctgatcatgaagctctgttgatgtgtgcagCCTCATGGATTCTTCAATTGTTCCCCTGCGATAG

3_5 aattacctgattgattgttttcata-ttttttttaccactctttttatgctcatggtaattacctgattgattgttttcatatttattaaacttcatctggagattttccaattttctgatcatgaagctctgttgatgtgtgcagCCTCATGGATTCTTCAATTGTTCCCCTGCGATAG

5_13 aattacctgattgattgttttcata-ttttttttaccactctttttatgctcatggtaattacctgattgattgttttcatatttattaaacttcatctggagattttccaattttctgatcatgaagctctgttgatgtgtgcagCCTCATGGATTCTTCAATTGTTCCCCTGCGATAG

6_16 aattacctgattgattgttttcata-ttttttttaccactctttttatgctcatggtaattacctgattgattgttttcatatttattaaacttcatcyggagrttttccaattttctgatcatgaagctctgttgatgtgtgcagCCTCATGGATTCTTCAATTGTTCCCCTGCGATAG

7_10 aattacctgattgattgttttcatatttttttttaccactctttttatgctcatggtaattacctgattgattgttttcatatttattaaacttcatcyggagrttttccaattktctgatcatgaagctctgttgatgtgtgcagCCTCATGGATTCTTCAATTGTTCCCCTGCGATAG

8_11 aattacctgattgattgttttcatatttttttttaccactctttttatgctcatggtaattacctgattgattgttttcatatttattaaacttcatccggaggttttccaattttctgatcatgaagctctgttgatgtgtgcagCCTCATGGATTCTTCAATTGTTCCCCTGCGATAG

9_8 -------------------------------------------------------------------------------------------cttcatccggaggttttccaattttctgatcatgaagctctgttgatgtgtgcagCCTCATGGATTCTTCAATTGTTCCCCTGCGATAG

10_4 aattacctgattgattgttttcatatttttttttaccactctttttatgctcatggtaattacmtgattgattgttttcatatttattaaacttcatcyggagrttttccaattttctgatcatgaagctctgttgatgtgtgcagCCTCATGGATTCTTCAATTGTTCCCCTGCGATAG

544

|

1_12 ATGTGCCACCTAATCCATGTGAATTGGATTCTAAAGATAATGACATCAAGGACAATGGTGCTTTGAAGCCAATTCAGAGTGCGTTAGCGGCAAAGCTTTAGgaacctttcgcaccaaaagttatggcaatgtgctgccgagagaaa

2_11 ATGTGCCACCTAATCCATGTGAATTGGATTCTAAAGATAATGACATCAAGGACAATGGTGCTTTGAAGCCAATTCAGAGTGCGTTAGCGGCAAAGCTTTAGgaacctttcgcaccaaaagttatggcaatgtgctgccgagaga--

3_5 ATGTGCCACCTAATCCATGTGAATTGGATTCTAAAGATAATGACATCAAGGACAATGGTGCTTTGAAGCCAATTCAGAGTGCGTTAGCGGCAAAGCTTTAGgaacctttcgcaccaaaagttatggcaa-----------------

5_13 ATGTGCCACCTAATCCATGTGAATTGGATTCTAAAGATAATGACATCAAGGACAATGGTGCTTTGAAGCCAATTCAGAGTGCGTTAGCGGCAAAGCTTTAGgaacctttcgcaccaaaagttatggcaatgtgctgccgagagaaa

6_16 ATGTGCCACCTAATCCATGTGAATTGGATTCTAAAGATAATGACATCAAGGACAATGGTGCTTTGAAGCCAATTCAGAGTGCGTTAGCGGCAAAGCTTTAGgaacctttcgcaccaaaagttatggcaatg---------------

7_10 ATGTRCCACCTAATCCATGTGAATTGGATTCTAAAGATAATGACATCAAGGACAATGGTGCTTTGAAGCCAATTCAGAGTGCGTTAGCGGCAAAGCTTTAGgaacctttcgcaccaaaagttatggcaatgtgctgccgagagaa-

8_11 ATGTGCCACCTAATCCATGTGAATTGGATTCTAAAGATAATGACATCAAGGACAATGGTGCTTTGAAGCCAATTCAGAGTGCGTTAGCGGCAAAGCTTTAGgaacctttcgcaccaaaagttatggcaatgtgctgccgagagaaa

9_8 ATGTGCCACCTAATCCATGTGAATTGGATTCTAAAGATAATGACATCAAGGACAATGGTGCTTTGAAGCCAATTCAGAGTGCGTTAGCGGCAAAGCTTTAGgaacctttcgcaccaaaagttatggcaatgtgctgccgagagaaa

10_4 ATGTGCCACCTAATCCATGTGAATTGGATTCTAAAGATAATGACATCAAGGACAATGGTGCTTTGAAGCCAATTCAGAGTGCGTTAGCGGCAAAGCTTTAGgaacctttcgcaccaaaagttatggcaatgtgctgccgagagaaa

**FIGURE S7E |** *SfCAO2* gDNA sequence (part of exon 10 to 3' UTR).

41 69 79

| | |

1_12 AACCGGTCTTTAGAAGAGACTAATATAGTTCTTTGgtgcgctctctctctctctctcttaaaaagagtatgttgctagtatttctctctgtaaaatagtttaaaacactgtccaacagtaccaagaaataacctcttctgtaatttatattacattatattaagattagagatgacataaat

2_11 AACCGGTCTTTAGAAGAGACTAATATAGTTCTTTGgtgcg--ctctctctctctctcttaaaaagagtatgttgctagtatttctctctgtaaaatagtttaaaacactgtccaacagtaccaagaaataacctcttctgtaatttatattacattatattaagattagagatgacataaat

3_5 AACCGGTCTTTAGAAGAGACTAATATAGTTCTTTGgtgcg--ctctctctctctctcttaaaaagagtatgttgctagtatttctctctgtaaaatagtttaaaacactgtccaacagtaccaagaaataacctcttctgtaatttatattacattatattaagattagagatgacataaat

4_15 -ACCGGTCTTTAGAAGAGACTAATATAGTTCTTTGgtgcg--ctctctctctctctcttaaaaagagtrtgttgctagyatttctctctgtaaaatagtttaaaacactgtccaacagtaccaagaaataacctcttctgtaatttatattacattatattaagattagagatgacataaat

5_13 AACCGGTCTTTAGAAGAGACTAATATAGTTCTTTGgtgcg--ctctctctctctctcttaaaaagagtrtgttgctagyatttctctctgtaaaatagtttaaaacactgtccaacagtaccaagaaataacctcttctgtaatttatattacattatattaagattagagatgacataaat

6_16 AACCGGTCTTTAGAAGAGACTAATATAGTTCTTTGgtgcg--ctctctctctctctcttaaaaagagtatgttgctagtatttctctctgtaaaatagtttaaaacactgtccaacagtaccaagaaataacctcttctgtaatttatattacattatattaagattagagatgacataaat

7_10 AACCGGTCTTTAGAAGAGACTAATATAGTTCTTTGgtgcg--ctctctctctctctcttaaaaagagtatgttgctagtatttctctctgtaaaatagtttaaaacactgtccaacagtaccaagaaataacctcttctgtaatttatattacattatattaagattagagatgacataaat

8_11 AACCGGTCTTTAGAAGAGACTAATATAGTTCTTTGgtgcg----ctctctctctctcttaaaaagagtrtgttgctagyatttctctctgtaaaatagtttaaaacactgtccaacagtaccaagaaataacctcttctgtaatttatattacattatattaagattagagatgacataaat

9_8 AACCGGTCTTTAGAAGAGACTAATATAGTTCTTTGgtgcg--ctctctctctctctcttaaaaagagtatgttgctagtatttctctctgtaaaatagtttaaaacactgtccaacagtaccaagaaataacctcttctgtaatttatattacattatattaagattagagatgacataaat

10_4 -ACCGGTCTTTAGAAGAGACTAATATAGTTCTTTGgtgcg--ctctctctctctctctnnnnnnnnnnnnnnnnnnnnnnnnnnnnnnnnnnnnnnnnnnnnnnnnnnnnnnnnnnnnnnnnnnnnnnnnnnnnnnnnnnnnnnnnnnnnnnnnnnnnnnnnnnnnnnnnnnnnnnnnnnnn

235

|

1_12 tccccaattccatctttttgttccaatccacacataaattccccaattccaattttttgttccaattcactttaatgttccacccttaaaatggtttcacattttgtcttctggagactaaccgtgttgtcttcagGTATGTATTTGGAGTCACACATGTTCCTCGTTTAGAAGACTGGC

2_11 tccccaattccatctttttgttccaatccacacataaattccccaattccaattttttgttccaattcactttaatgttccacccttaaaatggtttcacattttgtcttctggagactaaccgtgttgtcttcagGTATGTATTTGGAGTCACACATGTTCCTCGTTTAGAAGACTGGC

3_5 tccccaattccatctttttgttccaatccacacataaattccccaattccaattttttgttccaattcactttaatgttccacccttaaaatggtttcacattttgtcttctggagactaaccgtgttgtcttcagGTATGTATTTGGAGTCACACATGTTCCTCGTTTAGAAGACTGGC

4_15 tccccaattccatctttttgttccaatccacacataaattccccaattccaaytttttgttccaattcactttaatgttccacccttaaaatggtttcacattttgtcttctggagactaaccgtgttgtcttcagGTATGTATTTGGAGTCACACANNNNNNNNNNNNNNAAGACTGGC

5_13 tccccaattccatctttttgttccaatccacacataaattccccaattccaaytttttgttccaattcactttaatgttccacccttaaaatggtttcacattttgtcttctggagactaaccgtgttgtcttcagGTATGTATTTGGAGTCACACATGTTCCTCGTTTAGAAGACTGGC

6_16 tccccaattccatctttttgttccaatccacacataaattccccaattccaattttttgttccaattcactttaatgttccacccttaaaatggtttcacattttgtcttctggagactaaccgtgttgtcttcagGTATGTATTTGGAGTCACACATGTTCCTCGTTTAGAAGACTGGC

7_10 tccccaattccatctttttgttccaatccacacataaattccccaattccaattttttgttccaattcactttaatgttccacccttaaaatggtttcacattttgtcttctggagactaaccgtgttgtcttcagGTATGTATTTGGAGTCACACATGTTCCTCGTTTAGAAGACTGGC

8_11 tccccaattccatctttttgttccaatccacacataaattccccaattccaaytttttgttccaattcactttaatgttccacccttaaaatggtttcacattttgtcttctggagactaaccgtgttgtcttcagGTATGTATTTGGAGTCACACATNNNNNNNNNNNNNNAGACTGGC

9_8 tccccaattccatctttttgttccaatcaacacataaattccccaattccaattttttgttccaattcactttaatgttccacccttaaaatggtttcacattttgtcttctggagactaaccgtgttgtcttcagGTATGTATTTGGAGTCACACATGTTCCTCGTTTAGAAGACTGGC

10_4 nnnnnnnnnnnnnnnnnnnnnnnnnnnnnacacataaattccccaattccaaytttttgttccaattcactttaatgttccacccttaaaatggtttcacattttgtcttctggagactaaccgtgttgtcttcagGTATGTATTTGGAGTCACACATGTTCCTNNNNNNNAAGACTGGC

1_12 CTGTTATGCCAGTAGAGCACATTGGTTTTATGCTCATGgtaattacctgattgattgttttcata-ttttttttaccactcttttgattatagtgtgttgtttgcctttcatgtaagttgcttgatatgatgctcttgattttttaatttttcaatgttattatagaacacttggtggtaa

2_11 CTGTTATGCCAGTAGAGCACATTGGTTTTATGCTCATGgtaattacctgattgattgttttcata-ttttttttaccactcttttgattatagtgtgttgtttgcctttcatgtaagttgcttgatatgatgctcttgattttttaatttttcaatgttattatagaacacttggtggtaa

3_5 CTGTTATGCCAGTAGAGCACATTGGTTTTATGCTCATGgtaattacctgattgattgttttcata-ttttttttaccactcttttgattatagtgtgttgtttgcctttcatgtaagttgcttgatatgatgctcttgattttttaatttttcaatgttattatagaacacttggtggtaa

4_15 CTGTTATGCCAGTAGAGCACATTGGTTTTATGCTCATGgtaattacctgattgattgttttcata-ttttttttaccactcttttgattatagtgtgnnnnnnnnnnnnnnnnnnnnnnnnnnnnnnnnnnnnnnnnnnnnnnnnnnnnnnnnnnnnnnnnnnnnnnnnnnnnnnnnnnnn

5_13 CTGTTATGCCAGTAGAGCACATTGGTTTTATGCTCATGgtaattacctgattgattgttttcata-ttttttttaccactcttttgattatagtgtgttgtnnnnnnnnnnnnnnnnnnnnnnnnnnnnnnnnnnnnnnnnnnnnnnnnnnnnnnnnnnnnnnnnnnnnnnnnnnnnnnnn

6_16 CTGTTATGCCAGTAGAGCACATTGGTTTTATGCTCATGgtaattacctgattgattgttttcata-ttttttttaccactcttttgattatagtgtgttgtttgcctttcatgtaagttgcttgatatgatgctcttgattttttaatttttcaatgttattatagaacacttggtggtaa

7_10 CTGTTATGCCAGTAGAGCACATTGGTTTTATGCTCATGgtaattacctgattgattgttttcata-ttttttttaccactcttttgattatagtgtgttgtttgcctttcatgtaagttgcttgatatgatgctcttgattttttaatttttcaatgttattatagaacacttggtggtaa

8_11 CTGTTATGCCAGTAGAGCACATTGGTTTTATGCTCATGgtaattacctgattgattgttttcata-ttttttttaccactcttttgattatagtgtgttgtnnnnnnnnnnnnnnnnnnnnnnnnnnnnnnnnnnnnnnnnnnnnnnnnnnnnnnnnnnnnnnnnnnnnnnnnnnnnnnnn

9_8 CTGTTATGCCAGTAGAGCACATTGGTTTTATGCTCATGgtaattacctgattgattgttttcatatttttttttaccactcttttgattatagtgtgttgtttgcctttcatgtaagttgcttgatatgatgctcttgattttttaatttttcaatgttattatagaacacttggtggtaa

10_4 CTGTTATGCCAGTAGAGCACATTGGTTTTATGCTCATGgtaattacctgattgattgttttcata-ttttttttaccactcttttnnnnnnnnnnnnnnnnnnnnnnnnnnnnnnnnnnnnnnnnnnnnnnnnnnnnnnnnnnnnnnnnnnnnnnnnnnnnnnnnnnnnnnnnnnnnnnnn

1_12 gtgcttaaaacatcactagaatattaagaatgttgatttggggtagttgttattaggtccggtccaaaggtaaggttagacacacccttacttaggccctcaacccaccgcactcagtcccctatatagttcactaacaaaaattgcacatgtgaggatcccttccatgatttattcgct

2_11 gtgcttaaaacatcactagaatattaagaatgttgatttggggtagttgttattaggtccggtccaaaggtaaggttagacacacccttacttaggccctcaacccaccgcactcagtcccctatatagttcactaacaaaaattgcacatgtgaggatcccttccatgatttattcgct

3_5 gtgcttaaaacatcactagaatattaagaatgttgatttggggtagttgttattaggtccggtccaaaggtaaggttagacacacccttacttaggccctcaacccaccgcactcagtcccctatatagttcactaacaaaaattgcacatgtgaggatcccttccatgatttattcgct

4_15 nnnnnnnnnnnnnnnnnnnnnnnnnnnnnnnnnnnnnnnnnnnnnnnnnnnnnnnnnnnnnnnnnnnnnnnnnnnnnnnnnnnnnnnnnnnnnnnnnnnnnnnnnnnnnnnnnnnnnnnnnnnnnnnnnnnnnnnnnnnnnnnnnnnnnnnnnnnnnnnnnnnnnnnnnnnnnnnnnnnn

5_13 nnnnnnnnnnnnnnnnnnnnnnnnnnnnnnnnnnnnnnnnnnnnnnnnnnnnnnnnnnnnnnnnnnnnnnnnnnnnnnnnnnnnnnnnnnnnnnnnnnnnnnnnnnnnnnnnnnnnnnnnnnnnnnnnnnnnnnnnnnnnnnnnnnnnnnnnnnnnnnnnnnnnnnnnnnnnnnnnnnnn

6_16 gtgcttaaaacatcactagaatattaagaatgttgatttggggtagttgttattaggtccggtccaaaggtaaggttagacacacccttacttaggccctcaacccaccgcactcagtcccttatatagttcactaacaaaaattgcacatgtgaggatcccttccatgatttattcgct

7_10 gtgcttaaaacatcactagaatattaagaatgttgatttggggtagttgttattaggtccggtccaaaggtaaggttagacacacccttacttaggccctcaacccaccgcactcagtcccctatatagttcactaacaaaaattgcacatgtgaggatcccttccatgatttattcgct

8_11 nnnnnnnnnnnnnnnnnnnnnnnnnnnnnnnnnnnnnnnnnnnnnnnnnnnnnnnnnnnnnnnnnnnnnnnnnnnnnnnnnnnnnnnnnnnnnnnnnnnnnnnnnnnnnnnnnnnnnnnnnnnnnnnnnnnnnnnnnnnnnnnnnnnnnnnnnnnnnnnnnnnnnnnnnnnnnnnnnnnn

9_8 gtgcttaaaacatcactagaatattaagaatgttgatttggggtagttgttattaggtccggtccaaaggtaaggttagacacacccttacttaggccctcaacccaccgcactcagtcccctatatagttcactaacaaaaattgcacatgtgaggatcccttccatgatttattcgct

10_4 nnnnnnnnnnnnnnnnnnnnnnnnnnnnnnnnnnnnnnnnnnnnnnnnnnnnnnnnnnnnnnnnnnnnnnnnnnnnnnnnnnnnnnnnnnnnnnnnnnnnnnnnnnnnnnnnnnnnnnnnnnnnnnnnnnnnnnnnnnnnnnnnnnnnnnnnnnnnnnnnnnnnnnnnnnnnnnnnnnnn

736 801  **823**

| | **|**

1_12 ctaaggcgtcacttaattagctttatgcctcacattgccttgtgccgaccctggttgttatcggggacaatgtttagtgtagcacatcttttccatcaccgatgagcttttgttgtctctgttatttttttgggggggccttattcttttgtttatgttatttattaaacttcatctgga

2_11 ctaaggcgtcacttaattagctttatgcctcacattgccttgtgccgaccctggttgttatcggggacaatgtttagtatagcacatcttttccatcaccgatgagcttttgttgtctctgttatttttttgggggggccttattcttttgtttatgttatttattaaacttcatctgga

3_5 ctaaggcgtcacttaattagctttatgcctcacattgccttgtgccgaccctggttgttatcggggacaatgtttagtgtagcacatcttttccatcaccgatgagcttttgttgtctctgttatttttttgggggggccttattcttttgtttatgttatttattaaacttcatctgga

4_15 nnnnnnnnnnagtyaattagctttatgcctcacattgccttgtgccgaccctggttgttatcggggacaatgtttagtatagcacatcttttccatcaccaatgagcttttgttgtctctgttatttttttgggggggccttattcttttgtttatgttatttattaaacttcatctgga

5_13 nnnnnnnnnnngtyaattagctttatgcctcacattgccttgtgccgaccctggttgttatcggggacaatgtttagtatagcacatcttttccatcaccratgagcttttgttgtctctgttatttttttgggggggccttattcttttgtttatgttatttattaaacttcatctgga

6_16 ctaaggcgtcacttaattagctttatgcctcacattgccttgtgccgaccctggttgttatcggggacaatgtttagtrtagcacatcttttccatcaccaatgagcttttgttgtctctgttatttttttgggggggccttattcttttgtttatgttatttattaaacttcatctgga

7_10 ctaaggcgtcacttaattagctttatgcctcacattgccttgtgccgaccctggttgttatcggggacaatgtttagtrtagcacatcttttccatcaccgatgagcttttgttgtctctgttatttttttgggggggccttattcttttgtttatgttatttattaaacttcatctgga

8_11 nnnnnnnnnnngtyaattagctttatgcctcacattgccttgtgccgaccctggttgttatcggggacaatgtttagtatagcacatcttttccatcaccaatgagcttttgttgtctctgttatttttttgggggggccttattcttttgtttatgttatttattaaacttcatctgga

9_8 ctaaggcgtcacttaattagctttatgcctcacattgccttgtgccgaccctggttgttatcggggacaatgtttagtatagcacatcttttccatcaccaatgagcttttgttgtctctgttatttttttgggggggccttattcttttgtttatgttatttattaaacttcatctgga

10_4 nnnnnnnnnnnctyaattagctttatgcctcacattgccttgtgccgaccctggttgttatcggggacaatgtttagtatagcacatcttttccatcaccaatgagcttttgttgtctctgttatttttttgggggggccttattcttttgtttatgttatttattaaacttcatctgga

**FIGURE S7E (continued)**

909

|

1_12 ggttttccaattttctgatcatgaagctctgttgatgtgtgcagCCTCATGGATTCTTCAATTGTTCCCCTGCGATAGATGTGCCACCTAATCCATGTGAATTGGATTCTAAAGATAATGACATCAAGGACAATGGTGCTTTGAAGCCAATTCAGAGTGCGTTAGCGGCAAAGCTTTAGg

2_11 ggttttccaattttctgatcatgaagctctgttgatgtgtgcagCCTCATGGATTCTTCAATTGTTCCCCTGCGATAGATGTGCCACCTAATCCATGTGAATTGGATTCTAAAGATAATGACATCAAGGACAATGGTGCTTTGAAGCCAATTCAGAGTGCGTTAGCGGCAAAGCTTTAGg

3_5 ggttttycaattttctgatcatgaagctctgttgatgtgtgcagCCTCATGGATTCTTCAATTGTTCCCCTGCGATAGATGTGCCACCTAATCCATGTGAATTGGATTCTAAAGATAATGACATCAAGGACAATGGTGCTTTGAAGCCAATTCAGAGTGCGTTAGCGGCAAAGCTTTAGg

4_15 ggttttycaattttctgatcatgaagctctgttgatgtgtgcagCCTCATGGATTCTTCAATTGTTCCCCTGCGATAGATGTGCCACCTAATCCATGTGAATTGGATTCTAAAGATAATGACATCAAGGACAATGGTGCTTTGAAGCCAATTCAGAGTGCGTTAGCGGCAAAGCTTTAGg

5_13 ggttttycaattttctgatcatgaagctctgttgatgtgtgcagCCTCATGGATTCTTCAATTGTTCCCCTGCGATAGATGTGCCACCTAATCCATGTGAATTGGATTCTAAAGATAATGACATCAAGGACAATGGTGCTTTGAAGCCAATTCAGAGTGCGTTAGCGGCAAAGCTTTAGg

6_16 ggttttccaattttctgatcatgaagctctgttgatgtgtgcagCCTCATGGATTCTTCAATTGTTCCCCTGCGATAGATGTGCCACCTAATCCATGTGAATTGGATTCTAAAGATAATGACATCAAGGACAATGGTGCTTTGAAGCCAATTCAGAGTGCGTTAGCGGCAAAGCTTTAGg

7_10 ggttttccaattttctgatcatgaagctctgttgatgtgtgcagCCTCATGGATTCTTCAATTGTTCCCCTGCGATAGATGTGCCACCTAATCCATGTGAATTGGATTCTAAAGATAATGACATCAAGGACAATGGTGCTTTGAAGCCAATTCAGAGTGCGTTAGCGGCAAAGCTTTAGg

8_11 ggttttycaattttctgatcatgaagctctgttgatgtgtgcagCCTCATGGATTCTTCAATTGTTCCCCTGCGATAGATGTGCCACCTAATCCATGTGAATTGGATTCTAAAGATAATGACATCAAGGACAATGGTGCTTTGAAGCCAATTCAGAGTGCGTTAGCGGCAAAGCTTTAGg

9_8 ggttttccaattttctgatcatgaagctctgttgatgtgtgcagCCTCATGGATTCTTCAATTGTTCCCCTGCGATAGATGTGCCACCTAATCCATGTGAATTGGATTCTAAAGATAATGACATCAAGGACAATGGTGCTTTGAAGCCAATTCAGAGTGCGTTAGCGGCAAAGCTTTAGg

10_4 ggttttccaattttctgatcatgaagctctgttgatgtgtgcagCCTCATGGATTCTTCAATTGTTCCCCTGCGATAGATGTGCCACCTAATCCATGTGAATTGGATTCTAAAGATAATGACATCAAGGACAATGGTGCTTTGAAGCCAATTCAGAGTGCGTTAGCGGCAAAGCTTTAGg

1_12 aacctttcgcaccaaaagttatggcaatgtgctgccgagagaaa

2_11 aacctttcgcaccaaaagttatggcaatgtgctgccgagagaaa

3_5 aacctttcgcaccaaaagttatggcaatgtgctgccgagagaaa

4_15 aacctttcgcaccaaaagttatggcaatgtgctgccgagagaaa

5_13 aacctttcgcaccaaaagttatggcaatgtgctgccgagagaaa

6_16 aacctttcgcaccaaaagttatggcaatgtgctgccgagagaaa

7_10 aacctttcgcaccaaaagttatggcaatgtgctgccgagagaaa

8_11 aacctttcgcaccaaaagttatggcaatgtgctgccgagagaaa

9_8 aacctttcgcaccaaaagttatggcaatgtgctgccgagagaaa

10_4 aacctttcgcaccaaaagttatggcaatgtgctgccgagagaaa

**FIGURE S8 | (A)** Genomic DNA sequence (6520 nt) of *LaCAO* with translation (above the DNA sequence). Predicted intron / exon boundaries of *Lup000530* (exons (uppercase text), introns (lowercase text)) as provided at <https://legumeinfo.org/>, with the exception of premature termination of exon 7 (green text, changed to uppercase text). The 5' splice site of intron 7 likely uses a non-canonical sequence (GC, double underlined) that is the second most used splice site combination in plants (1.488%) compared to the canonical GT-AG (97.886%) (Frey and Pucker, 2020). DNA sequence was translated with “show translation” (UPPER TEXT option) at (<http://www.bioinformatics.org/sms/>). Lupin genomic sequence includes an ‘interrupted’ SSR sequence near the start of intron 10, (CT)_4_NNN(CT)_2_ (underlined) at the same relative position as found in the *SfCAO1* and *SfCAO2* genomic sequences (**Figure 5, Figures S7D,E**, **S10** and **Table S8**).

**A**

      1 M  V  P  P  S  C  A  C  C  S  A  G  N  D  S  A  I  I  P  H  I  A  A  A  A  A  P  S  A  D  
       1 ATGGTACCTCCTTCTTGTGCTTGTTGTTCAGCCGGTAATGACTCTGCCATCATTCCCCACATTGCGGCTGCCGCCGCTCCCTCTGCTGAC
      31 W  T  A  N  V  P  D  D  G  R  L  N  K  M  T  I  V  H  P  V  D  S  L  P  Q  P  S  I  N  A  
      91 TGGACTGCCAATGTCCCCGATGATGGCCGCCTAAATAAGATGACCATCGTTCATCCTGTCGACTCCCTACCGCAACCTTCCATCAATGCC
      61 K  G                                                                                      
     181 AAAGgtatgtatattttgtttcatacttctctttttttttttttttttttttatgtttctttattttaccactctcttgattgtacgaca
     271 aaaataacacaatatttggattccctacaactttagtgagtttgactttttgaaacaatgtttttatttatttaattataaatacataca
     361 gagtaagaaaagattattttaaaaagtgattgaagttattacagttgacagaatgcaaggctaaagaatcaacaacattggtcatatatt
     451 tgaatgaaatatgtatacattaataatataaattatatatagttaatgtatcatttattatttatttatattactttagagtaaataata
     541 ttaaataaatgatataataatattgtaaaaattagttaaactttcgatatatacatatttttcaaataaaattaacgttctcgtatataa
     631 aaataagacagaaagaatcaagaaaactcacgtagcattctaacatttaaatatatatctacactctatccaaatatacttgacaccttt
     721 ctacctagctctactcctctccgatgtgaagaactggaatataaagatgaaatttctctttgaagaatttttatttattttttaataaaa
     811 aataacatgtaattataatccttgaaatttttttgacaatgtcttaattctattcaatactgatccccactcctttgtactggcataaat
     901 ttactatggtctcttcattatctccaaaaaatctatacccaactcaaactaataccaggtacagtttgtatcattccatgtattatgaac
     991 ttataattatcaaaaaatacttaataattaaattttaatttaaatatatttttttatatttaattataagtctatgacatataaaatgac
    1081 aaaaactgtaggacaacaatatatatcctacaactttttgatgtgtgttgtccaaatgattgtacctaaaacattcattaaaaatcggtt
    1171 atgatatattacgttcggaaattgggtaagtttaaacttggaaaatttataagcaatattttaaacaacatacacaccatattttaatcc
    1261 aaaacactaactcattaggtatatggattctacgtgtatatatatataaaaaccctatatatatatatatgggaatataaaaattcatac
    1351 tcgactgcaataataatagtaaagttttctgcacccttaagtttaatatggggctcaattctgttatgtttcaatgaaatatttgaagtt
    1441 cactttcttaactcaaccattgtgtttatcccacttttacttttccttcaagaaaatgaaatttaaaacttcggtagttgctaattgtca
    1531 ctcgaaattgttgctgaaggtccttatttaagtaaggactaagaacatagcaatataagtttttgaacctgaatcaaatgacatgttttt
    1621 ccaatcacacctattgcgactgatgcatttcttgttggacgtaattgaatgcatcaatttctctttggtggtcggtgattttggtggatt
    1711 tagaacacaattgataaatcttgaactcaactaactagtatttatattgctaacatttaatcaatcataaaactattgcgaagtcactaa
    1801 atctggatttcgtcaattgagttcaagctcggtcaacattatgttgtttcatattggttggcggttgatgtcactatagtcaccaatatc
    1891 atactagtaaacaaccaatattttaaatcacggttgcgatcgcaattatgatcacggttgtggttactgcgattgcggctattgcaatgc
    1981 gatttgcgaccaatacagccaaatgtagtcaaatacagatgcgttacggcatgatgcatccatgtcacgtgatgccatccatagttttaa
      63                                                I  I  T  L  P  R  P  Q  P  S  H  P  L  D  P
    2071 tcaatgttagcaccggtatcttatacttgctataatgttgcacagGAATCATTACACTGCCAAGGCCTCAACCAAGCCACCCTTTGGACC
      78   L  S  P  A  E  I  S  L  A  V  A  T  V  R  A  A  G  K  T  P  E                           
    2161 CTTTATCTCCTGCTGAAATCTCTCTGGCAGTAGCTACTGTGAGGGCTGCTGGAAAAACTCCTGAGgttgttagtcacattatgacttgtt
    2251 aaattcttcaaaggaacatatttgcatacttttcttgtaaatatgagtctatgtaacttgaagcagtgattggttactaacgattccaac
      99                                  L  K  D  G  L  R  F  M  E  I  A  L  L  E  P  D  K  H  V  
    2341 ttgtttctcccaaattgtatttatttgttgcagCTTAAAGACGGTTTGCGATTCATGGAAATAGCTTTGCTCGAACCGGATAAACATGTC
     118 V  A  L  A  D  A  Y  F  F  P  P  F  Q  P  S  L  L  P  K  G  G  F  V  I  P  T  K  L  P  P  
    2431 GTTGCACTAGCAGATGCTTATTTTTTTCCACCTTTCCAGCCATCATTGCTTCCTAAAGGAGGGTTTGTGATCCCAACTAAACTCCCTCCA
     148 R  C  A  R  L  L  V  Y  N  R  K  T  N  E  T  S  L  W  I  V  E  L  S  Q  V  H  A  V  T  R  
    2521 AGATGTGCTAGACTTCTTGTTTACAATAGGAAGACAAATGAGACTAGTCTTTGGATCGTCGAGTTATCGCAAGTTCATGCTGTAACTCGA
     178 G  G  N  H  L  G  K  V  I  S  S  Q  V  V  P  D  V  Q  P  P  M                             
    2611 GGTGGAAATCATTTAGGAAAAGTAATTTCATCACAAGTTGTACCTGATGTTCAGCCTCCAATGgtatgttgttcttttcctcacaacttc
    2701 tgattccctctttttacattttgtatgaactaatgtctttttaaataattttaagtcatttcaaatggtattgcatcaaatcattttgca
    2791 catacagtttaagaaacaattgcagaatatgttgtacaagttgaagcagtctctgtagtgagatgtcttacttttactttgttacatttt
    2881 ctattgtttataaatgagagtttcatattagagtaatgaatagcataacatgttaaaatattgaaattcttatatattttatgtattatt
    2971 ttttcattcctacactaaggagttcataaactgcatatctactctttccaattaagaagagaacatagttgttagttgggaagattggta
    3061 aaatctgaaattggaggtcctagttttgttctgaaagaaaatccctagagttttcaaaacttgtaaaataataatatcaaaactgttaat
    3151 accgataaatttgtcaagatcctactattttaatgtccaaaaaattatgagacccactgtagtagatgtacgcagtcttacataaatgct
     199                               D  A  V  E  Y  A  E  C  E  A  A  V  K  S  Y  P  P  F  I  E  
    3241 tcaattctgtattattctttcgatttgcagGATGCTGTGGAGTATGCAGAATGTGAGGCTGCTGTTAAAAGTTATCCTCCATTTATAGAG
     219 A  M  K  K  R  G  I  E  N  M  E  L  V  M  V  D  P  W                                      
    3331 GCTATGAAGAAAAGGGGTATTGAAAACATGGAGCTTGTGATGGTAGATCCCTGgtaaataaattcagatatccaatgtttatagagatct
     237                                                                             C  A  G  Y  F 
    3421 tgtttggagacattgccattctttctcttagaatttcatcattccaaacttttttactacatatttatgctgcagGTGTGCTGGTTACTT
     242  S  E  A  D  D  P  N  R  R  L  A  K  P  I  I  F  C  K  C  E  S  D  C  P  M  E  N  G  Y  A 
    3511 CAGTGAAGCTGATGATCCGAACCGAAGACTTGCTAAACCAATAATATTTTGCAAGTGTGAGAGTGATTGCCCTATGGAAAATGGCTATGC
     272  R  P  V  E  G  I  F  V  L  V  D  M  Q  K  M  E  V  I  Q  F  E  D  R  K  L  V  P  L  P  P 
    3601 TCGCCCGGTCGAGGGAATCTTTGTTCTTGTTGATATGCAAAAGATGGAGGTGATACAGTTCGAAGACCGCAAACTTGTTCCTCTGCCTCC
     302  V  D  P  L  R  N  Y  T  H  A  A  T  R  G  G  T  D  R  S  D  L  K  P  L  K  I  V  Q  P  E 
    3691 TGTAGATCCCTTAAGGAACTATACACATGCTGCAACTAGAGGTGGCACTGATAGAAGTGACTTAAAACCATTGAAAATTGTTCAACCTGA
     332  G  P  S  F  S  V  N  G  Y  Y  V  E  W  Q  K                                              
    3781 AGGTCCAAGCTTTTCCGTCAATGGATATTATGTTGAATGGCAAAAGgtctagaagaacttctgcattgtaaattgtaactactaattact
     347                                             W  N  F  R  I  G  F  T  P  K  E  G  L  V  I  Y
    3871 gaacttgcaacttggcatataacaatttttgtctttgttaacagTGGAACTTTCGGATTGGATTCACACCCAAAGAAGGTTTAGTTATAT
     363   S  V  A  Y  V  D  G  S  Q  G  L  R  P  V  A  H  R  L  S  F  V  E  M  V  V  P  Y  G  D  P
    3961 ATTCTGTTGCATATGTTGATGGTAGTCAAGGTCTAAGGCCTGTAGCTCATAGGTTGAGTTTTGTGGAGATGGTTGTACCCTACGGAGATC
     393   N  D  P  H  Y  R  K  N  A  F  D  A  G  E  D  G  L  G  R  N  A  H  S  L  K  K            
    4051 CAAACGATCCACATTACAGGAAAAATGCTTTTGATGCTGGGGAAGATGGCCTAGGAAGAAATGCACATTCCTTGAAGAAGgtttaagact
    4141 tacacatttattatcttcacatacatgtattgttttgagtttactatttctgattggattctcccatttgtctaagtccaagtttgttgt
     419      G  C  D  C  S  G  I  V  K  Y  F  D  A  H  F  T  N  F  T  G  G  V  E  T  I  E  N  C  V 
    4231 tacagGGATGTGATTGTTCTGGCATAGTCAAATATTTTGATGCTCACTTCACAAATTTCACTGGTGGTGTGGAGACAATTGAAAATTGTG

C L H E E D H G I L W K H Q D W R T G L S E V R R S R R L S

    4321 TATGTTTGCATGAAGAAGATCATGGAATTCTTTGGAAGCATCAAGATTGGAGAACTGGCTTATCAGAAGTCCGAAGGTCTAGAAGGCTTT

V S F I C T V A N Y E Y G F F W H F Y Q
    4411 CAGTTTCATTTATATGTACTGTGGCTAACTATGAGTATGGATTTTTTTGGCACTTTTATCAGgcaagtacaatatcacttcctttcaaga
    4501 gatctatatgcttaccttaatgaaattttaaagtatagttatatgattcgtggtatcggttctagtatgcagtactcggttcaatgctgt

    4591 catcattttttaaattattattacaaagacttcatttagatggaactcaatacgttgtatgccatatgccaaacctggcacattcagaaa
     447                                                                                      D  G 
    4681 ctttaaaaaatatgttgttgttttgattttactgaactacttgtgtatacaaatttgtcgaaaatgatggcatgtgttgtttcagGATGG
     449  K  M  E  A  E  V  K  L  T  G  I  L  S  M  G  A  L  M  P  G  E  Y  R  K  Y  G  T  V  I  A 
    4771 AAAGATGGAAGCTGAAGTTAAGCTAACTGGAATTCTGAGCATGGGAGCCTTAATGCCCGGAGAGTATCGAAAATATGGAACCGTGATTGC
     479  P  G  L  Y  A  P  V  H  Q  H  F  F  V  A  R  M  N  M  A  V  D  S  R  P  G  E  A  L  N  Q 
    4861 CCCAGGTCTATATGCTCCAGTTCATCAACACTTTTTTGTTGCTCGTATGAACATGGCTGTTGATTCTAGACCTGGTGAAGCTTTGAATCA
     509                                                                                           
    4951 Ggtaatttgaattaacactttatgttgttcttcaacaattttaccacttttttaacagtgaaataactcagctactttcatgttttgttt
    5041 gcaatttcacttttatacagtcctacttgtctaataatttatgccactttgctattgcataacaattcatttttcaagtcaacattctta
     509                     V  V  E  V  N  V  K  A  E  E  P  G  D  H  N  V  H  N  N  A  F  Y  A  E
    5131 ctcttaggaatttttgttagGTTGTGGAAGTCAATGTGAAAGCTGAGGAACCTGGTGATCATAATGTTCACAATAATGCATTCTATGCCG
     533   E  T  L  L  R  S  E  M  E  A  M  R  D  C  D  P  M  T  A  R  S  W  I                     
    5221 AAGAAACTTTGCTCAGATCTGAAATGGAAGCAATGCGTGATTGCGATCCCATGACTGCTCGATCTTGGATTgtaagtcttccccgggttt
    5311 tacagccaactttggactatggattgctaataatgtcctactttctcatactactgaattcttatctgctatgttgaaaaacattgttgc
    5401 tcctagcctaaataaacatatcagatgaatgccccttaatgtttgataaactttatcatcattttcatatagaatttagcatatgctctt
     556                                                                               V  R  N  T  
    5491 agcaattccaaaacattatcttcaattctcataggagtaagcatatcattgactatttactttttaacgatccttcagGTAAGGAATACA
     560 R  S  T  N  R  T  G  H  L  T  G  Y  K  L  V  P  G  S  N  C  L  P  F  A  H  S  D  A  K  F  
    5581 AGATCAACCAATAGAACTGGACACTTGACAGGCTACAAGCTAGTACCTGGCTCGAACTGCTTACCATTCGCGCATTCGGATGCCAAGTTT
     590 L  R  R  G  A  F  L  K  H  N  L  W  V  T  A  Y  S  P  D  E  L  F  P  G  G  E  F  P  N  Q  
    5671 TTAAGAAGAGGTGCTTTCTTGAAGCATAATCTTTGGGTTACAGCTTACTCACCCGATGAGCTGTTTCCTGGAGGAGAATTTCCTAATCAA
     620 N  P  R  I  G  D  G  L  P  T  W  V  T  Q  N  R  S  L  E  E  S  D  I  V  L  W              
    5761 AATCCACGCATTGGCGACGGATTACCTACATGGGTTACGCAGAACCGATCTTTAGAAGAGTCTGATATAGTTCTTTGgtattctctctct
    5851 cagtctctcacacacattccaaaaaaattcacaaaatttgtaatgctaaattattaaagcactacttttatatgtaagtttccatattta
    5941 gtgatttatatttcaatatatcaaagagatgatataagtattattccacccttgaaacaaaaattcacattgtgtcttctcaactaacta
     646                   Y  V  F  G  V  T  H  V  P  R  L  E  D  W  P  V  M  P  V  E  H  I  G  F  
    6031 tgatatgttatcttcagGTATGTATTTGGAGTCACACATGTTCCTCGTTTAGAAGACTGGCCTGTTATGCCAGTAGAGCACATTGGTTTT
     670 M  L  M                                                                                   
    6121 ATGCTCATGgtaatttatgtgattccttttttcaagaccaaacttttttgagaaaagaatttatgcatgaaagagatttatacactatca
    6211 gtccatgatgcatcgtttatttttaattattattccccctttaaaaaaagtaatacaaatgaatgatgaataatatactcatgactgtgt
     673                                                                                      P  H 
    6301 ataaatatttacactgactgttcatatatattacactcatcttttgttcatgttatgtatttaactgcatttttttattttgcagCCTCA
     675  G  F  F  N  C  S  P  A  I  D  V  P  P  S  K  C  E  L  E  A  K  E  K  D  I  K  D  N  G  V 
    6391 TGGATTCTTCAATTGTTCCCCTGCAATAGATGTTCCACCTAGTAAATGTGAATTGGAGGCTAAAGAAAAAGATATAAAGGATAATGGGGT
     705  L  K  P  I  E  N  S  L  A  S  K  L  *  
    6481 TTTGAAGCCAATTGAGAATTCCTTAGCATCAAAGCTCTAA

**FIGURE S8 | (B)** Alignment of predicted protein sequences of *S. flavescens,* SfCAO and *L. angustifolius*, LaCAO. Sequences are from this study (SfCAO, **Figures 5, S4**) and LaCAO (Lup000530, <https://legumeinfo.org/>). SfCAO and LaCAO are 77% identical (84.6% similarity), based on alignment using Needle (<https://www.ebi.ac.uk/Tools/psa/emboss_needle/>). Conserved residues are indicated, NYE/X motif (double underlined) and the three histidines (*) that interact with the catalytic copper ion (Yang et al., 2017). The copper amine oxidase domain (Pfam, PF01179) spans from QIIQ (exon 4) to VPP (exon 12). Yellow highlights show the amino acid residues that are encoded by DNA sequence that spans an intron, based on introns in *Lup000530* (**Figure S8A**).

**B**

SfCAO 1 MASVSQKVAPPS-PCCSPGGDSNHIPLHAAATSSAETQDWTDTISDDRRPNTVALVRPVDSLPVPPTNAPTVKGITTMPRPQSSHPLDPLSAAEISVAVA

:.||| .|||.|.||..|| |.|| ::|.:.|||..:.||.|.|.:.:|.||||||.|..|| |||.|:||||.||||||||.||||:|||

LaCAO 1 -------MVPPSCACCSAGNDSAIIP-HIAA-AAAPSADWTANVPDDGRLNKMTIVHPVDSLPQPSINA---KGIITLPRPQPSHPLDPLSPAEISLAVA

SfCAO 100 TVRAAGSTPELRDSMRFLEVVLVEPDKHVIALADAYFFPPFQPSLLHRTKGGPLIPTKLPPRCAKLVVYSRKTNETSIWIVELSQAHAVTRGGHHRGKVI

||||||.||||:|.:||:|:.|:||||||:|||||||||||||||| .|||.:||||||||||:|:||:|||||||:|||||||.|||||||:|.||||

LaCAO 89 TVRAAGKTPELKDGLRFMEIALLEPDKHVVALADAYFFPPFQPSLL--PKGGFVIPTKLPPRCARLLVYNRKTNETSLWIVELSQVHAVTRGGNHLGKVI

SfCAO 200 VSHVIPEVQPPMDAVEYAECEAAVKSFPPFIEAMKKRGVEDMDLVMVDPWCAGYFSEADAPKRRLAKPLIFCRSESDCPMENGYARPVEGIFVLVDMQNM

.|.|:|:|||||||||||||||||||:|||||||||||:|:|:||||||||||||||||.|.||||||:|||:.||||||||||||||||||||||||.|

LaCAO 187 SSQVVPDVQPPMDAVEYAECEAAVKSYPPFIEAMKKRGIENMELVMVDPWCAGYFSEADDPNRRLAKPIIFCKCESDCPMENGYARPVEGIFVLVDMQKM

SfCAO 300 VVIEFEDRKLVPLPPVDPLRNYTRGETRGGTDRSDVKPLQIIQPEGPSFRVNGYYVEWQKWNFRVGFTPKEGLVIYSVAYVDGSRGRRPVAHRLSFVEMV

.||:|||||||||||||||||||...|||||||||:|||:|:|||||||.||||||||||||||:|||||||||||||||||||:|.|||||||||||||

LaCAO 287 EVIQFEDRKLVPLPPVDPLRNYTHAATRGGTDRSDLKPLKIVQPEGPSFSVNGYYVEWQKWNFRIGFTPKEGLVIYSVAYVDGSQGLRPVAHRLSFVEMV

SfCAO 400 VPYGDPNDPHYRKNAFDAGEDGLGRNAHSLKKGCDCLGFIKYFDAHFTSFTGGVETIENCVCMHEEDHGILWKHQDWRTGLAEVRRSRRLTVSFICTVA**N**

||||||||||||||||||||||||||||||||||||.|.:||||||||:|||||||||||||:||||||||||||||||||:||||||||:|||||||||

LaCAO 387 VPYGDPNDPHYRKNAFDAGEDGLGRNAHSLKKGCDCSGIVKYFDAHFTNFTGGVETIENCVCLHEEDHGILWKHQDWRTGLSEVRRSRRLSVSFICTVA**N**

* *

SfCAO 500 **YE**YGFFWHFYQDGKIEAEVKLTGILSLGALMPGEYRKYGTMIAPGLYAPV**H**Q**H**FFVARMDMAVDSKPGEALNQVVEVNVKIEEPGENNVHNNAFYAEETL

||||||||||||||:|||||||||||:|||||||||||||:||||||||||||||||||:|||||:||||||||||||||.||||::|||||||||||||

LaCAO 487 **YE**YGFFWHFYQDGKMEAEVKLTGILSMGALMPGEYRKYGTVIAPGLYAPV**H**Q**H**FFVARMNMAVDSRPGEALNQVVEVNVKAEEPGDHNVHNNAFYAEETL

SfCAO 600 LRSELEAVRDCNPMTARHWIVRNTRSSNRTGELTGYKLVPGSNCLPLAGSDAKFLRRAAFLKHNLWVTAYSPDEMFPGGEFPNQNPRIGEGLPTWVKQNR

||||:||:|||:|||||.||||||||:||||.||||||||||||||.|.||||||||.||||||||||||||||:||||||||||||||:||||||.|||

LaCAO 587 LRSEMEAMRDCDPMTARSWIVRNTRSTNRTGHLTGYKLVPGSNCLPFAHSDAKFLRRGAFLKHNLWVTAYSPDELFPGGEFPNQNPRIGDGLPTWVTQNR

*

SfCAO 700 SLEETNIVLWYVFGVT**H**VPRLEDWPVMPVEHIGFMLMPHGFFNCSPAIDVPPNPCELDSKDNDIKDNGALKPIQSALAAKL* 780

||||::||||||||||||||||||||||||||||||||||||||||||||||:.|||::|:.||||||.||||:::||:||

LaCAO 687 SLEESDIVLWYVFGVT**H**VPRLEDWPVMPVEHIGFMLMPHGFFNCSPAIDVPPSKCELEAKEKDIKDNGVLKPIENSLASKL* 768

**----------------**

**exon 11**

**FIGURE S9 |** PCR suggests two *SfCAO* genes related to *LaCAO* cDNA (MF152953.1) involved in QA metabolism. *SfCAO1* and *SfCAO2* differ mostly in the length of their introns. **(A)** Two strong PCR products were obtained with most genotypes when attempting to PCR a genic fragment using a ‘forward’ primer for exon 10 (SfCAO_e10_F) and a ‘reverse’ primer in the 3' UTR (SfCAO_R1). Most genotypes produced a ‘lower’ band of the expected size (compared to lupin gene (Lup000530), ≈ 750 basepairs (bp), see **Figure S8**) and an ‘upper’ band (≈1200 bp). Sample 4_15 did not produce a 750 bp band. M represents the 1 kb plus DNA marker (Thermofisher, Australia). To minimize UV exposure and thus DNA damage the gel image was taken on a blue-light transilluminator (Safe Imager^TM^, Invitrogen) with a handheld phone camera and low ambient light creating the uneven background observed. PCR products of sample 7_10 are faint, due to low DNA concentration, and sufficient products were obtain when PCR was repeated using more template DNA. **(B)** Sequences of the 685 bp 3' *SfCAO* PCR product (excluding primer sequence), hereafter called *SfCAO1*, includes the 3' end of exon 10 (35 nt, green uppercase text), intron 10 (241 nt, lowercase), exon 11 (82 nt, purple uppercase text), intron 11 (147 nt, lowercase) and exon 12 (135 nt, uppercase blue text), and 45 nt of 3' UTR. **(C)** Sequence of the 1126 bp 3' *SfCAO* PCR product (excluding primer sequence) from sample 1_12, hereafter called *SfCAO2*, has the same exon sequence however intron 10 is 40 nt longer (at 281 nt) and intron 11 is 399 nt longer (at 546 nt). **(D)** Alignment of the 3' sequence of *SfCAO1* and *SfCAO2* (from sample 1_12).

**A**


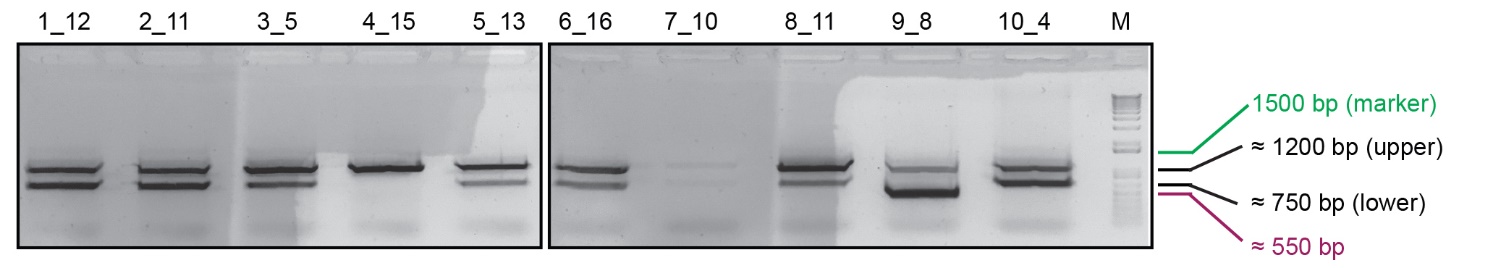


**B**

>SfCAO1_1_12_partial_3prime_685nt_RefSeq

**AACCGGTCTTTAGAAGAGACTAATATAGTTCTTTG**gtgcgctctctctctctcttaaaaaagagtgtactgctaatatttttctctataaaatagtttaaaacactgtccaacagtaccaagaaataacctcttctgtaatttatattacattatattaagattagagatgacataaattccccaattccaactttttgttccaatccactttaatgttccacccttaaaatggtttcacattttgtcttctggagactaaccgtgttatcttcag**GTATGTATTTGGAGTCACACATGTTCCTCGTTTAGAAGACTGGCCTGTTATGCCAGTAGAGCACATTGGTTTTATGCTCATG**gtaattacctgattgattgttttcatattttttttaccactctttttatgctcatggtaattacctgattgattgttttcatatttattaaacttcatctggagattttccaattttctgatcatgaagctctgttgatgtgtgcag**CCTCATGGATTCTTCAATTGTTCCCCTGCGATAGATGTGCCACCTAATCCATGTGAATTGGATTCTAAAGATAATGACATCAAGGACAATGGTGCTTTGAAGCCAATTCAGAGTGCGTTAGCGGCAAAGCTTTAG**gaacctttcgcaccaaaagttatggcaatgtgctgccgagagaaa

**C**

>SfCAO2_1_12_partial_3prime_1126nt_RefSeq

**AACCGGTCTTTAGAAGAGACTAATATAGTTCTTTG**gtgcgctctctctctctctctcttaaaaagagtatgttgctagtatttctctctgtaaaatagtt

taaaacactgtccaacagtaccaagaaataacctcttctgtaatttatattacattatattaagattagagatgacataaattccccaattccatctttt

tgttccaatccacacataaattccccaattccaattttttgttccaattcactttaatgttccacccttaaaatggtttcacattttgtcttctggagac

taaccgtgttgtcttcag**GTATGTATTTGGAGTCACACATGTTCCTCGTTTAGAAGACTGGCCTGTTATGCCAGTAGAGCACATTGGTTTTATGCTCATG**

gtaattacctgattgattgttttcatattttttttaccactcttttgattatagtgtgttgtttgcctttcatgtaagttgcttgatatgatgctcttga

ttttttaatttttcaatgttattatagaacacttggtggtaagtgcttaaaacatcactagaatattaagaatgttgatttggggtagttgttattaggt

ccggtccaaaggtaaggttagacacacccttacttaggccctcaacccaccgcactcagtcccctatatagttcactaacaaaaattgcacatgtgagga

tcccttccatgatttattcgctctaaggcgtcacttaattagctttatgcctcacattgccttgtgccgaccctggttgttatcggggacaatgtttagt

gtagcacatcttttccatcaccgatgagcttttgttgtctctgttatttttttgggggggccttattcttttgtttatgttatttattaaacttcatctg

gaggttttccaattttctgatcatgaagctctgttgatgtgtgcag**CCTCATGGATTCTTCAATTGTTCCCCTGCGATAGATGTGCCACCTAATCCATGT**

**GAATTGGATTCTAAAGATAATGACATCAAGGACAATGGTGCTTTGAAGCCAATTCAGAGTGCGTTAGCGGCAAAGCTTTAG**gaacctttcgcaccaaaag

ttatggcaatgtgctgccgagagaaa

**FIGURE S9 | continued**

**D**

SfCAO1_1_12 1 **AACCGGTCTTTAGAAGAGACTAATATAGTTCTTTG**gtgcgctctctctctctct---taaaaaagagtgtactgctaatatttttctctataaaatagtt 97

**|||||||||||||||||||||||||||||||||||**||||||||||||||||||| |.|||||||||.|..|||||.|||||.|||||.||||||||||

SfCAO2_1_12 1 **AACCGGTCTTTAGAAGAGACTAATATAGTTCTTTG**gtgcgctctctctctctctctcttaaaaagagtatgttgctagtatttctctctgtaaaatagtt 100

SfCAO1_1_12 98 taaaacactgtccaacagtaccaagaaataacctcttctgtaatttatattacattatattaagattagagatgacataaattccccaattccaactttt 197

||||||||||||||||||||||||||||||||||||||||||||||||||||||||||||||||||||||||||||||||||||||||||||||.|||||

SfCAO2_1_12 101 taaaacactgtccaacagtaccaagaaataacctcttctgtaatttatattacattatattaagattagagatgacataaattccccaattccatctttt 200

SfCAO1_1_12 198 tgttccaatc---------------------------------------cactttaatgttccacccttaaaatggtttcacattttgtcttctggagac 258

|||||||||| |||||||||||||||||||||||||||||||||||||||||||||||||||

SfCAO2_1_12 201 tgttccaatccacacataaattccccaattccaattttttgttccaattcactttaatgttccacccttaaaatggtttcacattttgtcttctggagac 300

SfCAO1_1_12 259 taaccgtgttatcttcag**GTATGTATTTGGAGTCACACATGTTCCTCGTTTAGAAGACTGGCCTGTTATGCCAGTAGAGCACATTGGTTTTATGCTCATG** 358

||||||||||.|||||||**||||||||||||||||||||||||||||||||||||||||||||||||||||||||||||||||||||||||||||||||||**

SfCAO2_1_12 301 taaccgtgttgtcttcag**GTATGTATTTGGAGTCACACATGTTCCTCGTTTAGAAGACTGGCCTGTTATGCCAGTAGAGCACATTGGTTTTATGCTCATG** 400

SfCAO1_1_12 359 gtaattacctgattgattgttttcatattttttttaccactcttt-------------------------------------------ttatgctcatg- 414

||||||||||||||||||||||||||||||||||||||||||||| |.||||||.||

SfCAO2_1_12 401 gtaattacctgattgattgttttcatattttttttaccactcttttgattatagtgtgttgtttgcctttcatgtaagttgcttgatatgatgctcttga 500

SfCAO1_1_12 415 -----------------gtaatta---------------------------------------------------------------------------- 421

||.||||

SfCAO2_1_12 501 ttttttaatttttcaatgttattatagaacacttggtggtaagtgcttaaaacatcactagaatattaagaatgttgatttggggtagttgttattaggt 600

SfCAO1_1_12 422 ---------------------------------------------------------------------------------------------------- 421

SfCAO2_1_12 601 ccggtccaaaggtaaggttagacacacccttacttaggccctcaacccaccgcactcagtcccctatatagttcactaacaaaaattgcacatgtgagga 700

SfCAO1_1_12 422 -----------------------------------------------------------------------cctgattgat-----------tgttt--- 436

||||.|||.| |||||

SfCAO2_1_12 701 tcccttccatgatttattcgctctaaggcgtcacttaattagctttatgcctcacattgccttgtgccgaccctggttgttatcggggacaatgtttagt 800

SfCAO1_1_12 437 -----------------tca------------------------------------------------------------tatttattaaacttcatctg 459

||| ||||||||||||||||||||

SfCAO2_1_12 801 gtagcacatcttttccatcaccgatgagcttttgttgtctctgttatttttttgggggggccttattcttttgtttatgttatttattaaacttcatctg 900

SfCAO1_1_12 460 gagattttccaattttctgatcatgaagctctgttgatgtgtgcag**CCTCATGGATTCTTCAATTGTTCCCCTGCGATAGATGTGCCACCTAATCCATGT** 559

|||.||||||||||||||||||||||||||||||||||||||||||**||||||||||||||||||||||||||||||||||||||||||||||||||||||**

SfCAO2_1_12 901 gaggttttccaattttctgatcatgaagctctgttgatgtgtgcag**CCTCATGGATTCTTCAATTGTTCCCCTGCGATAGATGTGCCACCTAATCCATGT** 1000

SfCAO1_1_12 560 **GAATTGGATTCTAAAGATAATGACATCAAGGACAATGGTGCTTTGAAGCCAATTCAGAGTGCGTTAGCGGCAAAGCTTTAG**gaacctttcgcaccaaaag 659

**|||||||||||||||||||||||||||||||||||||||||||||||||||||||||||||||||||||||||||||||||**|||||||||||||||||||

SfCAO2_1_12 1001 **GAATTGGATTCTAAAGATAATGACATCAAGGACAATGGTGCTTTGAAGCCAATTCAGAGTGCGTTAGCGGCAAAGCTTTAG**gaacctttcgcaccaaaag 1100

SfCAO1_1_12 660 ttatggcaatgtgctgccgagagaaa 685

||||||||||||||||||||||||||

SfCAO2_1_12 1101 ttatggcaatgtgctgccgagagaaa 1126

**
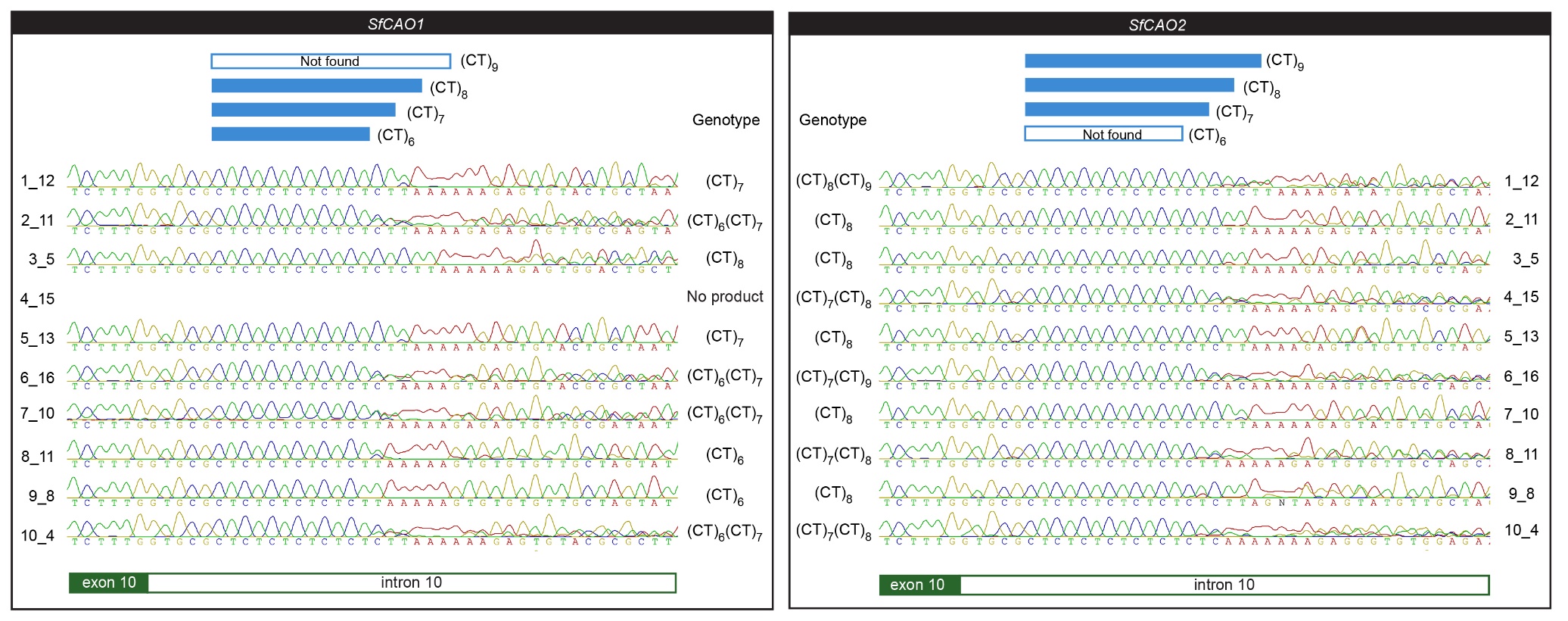
**

**FIGURE S10 |** Chromatograms showing different length SSR alleles for *SfCAO1* and *SfCAO2*. The SSR is near the start of intron 10 (**Figure 7D,E**). The length of the CT repeats for *SfCAO1* (left panel) range from (CT)_6_ to (CT)_8_, whereas for *SfCAO2* (right panel), they range from (CT)_7_ to (CT)_9_. SSR genotypes are summarised in **Table S8**. Individuals that are heterozygous for SSR length do not produce readable sequence after the SSR.

**Figure S11** | PCR comparison of *CAO* products from genomic DNA and cDNA. **(A)** PCR with genomic DNA extracted from two field grown plants, 1_LC1 and 9_AC1, showed that 9_AC1 contains the deletion variant of *SfCAO1* missing exon 11 (556 nt), as do most of the other samples from this population (**Figure 6**), and has the expected size product (1179 nt) for *SfCAO2.* Surprisingly, 1_LC1 only produced a PCR product for *SfCAO1* (738 nt, that includes exon 11), but not *SfCAO2,* providing another example of genetic variation within the *SfCAO* gene family (most likely as a result of changes at one or both primer binding sites), even with touchdown PCR. **(B)** Plants 1_LC1 and 9_AC1 both produce full length *SfCAO* cDNA sequences (≈ 2350 nt) and sequencing confirmed both products contained the 82 nt sequence of exon 11 (**Figure S4F**). **(C)** PCR did not amplify a smaller cDNA product for the deletion variant of *SfCAO1* in 9_AC1*,* as both 1_LC1 and 9_AC1 have the same size product, even when a smaller PCR product is amplified (350 nt, using SfCAO_e10_F and SfCAO_R1, that bind to exon 10 and 3' UTR respectively).


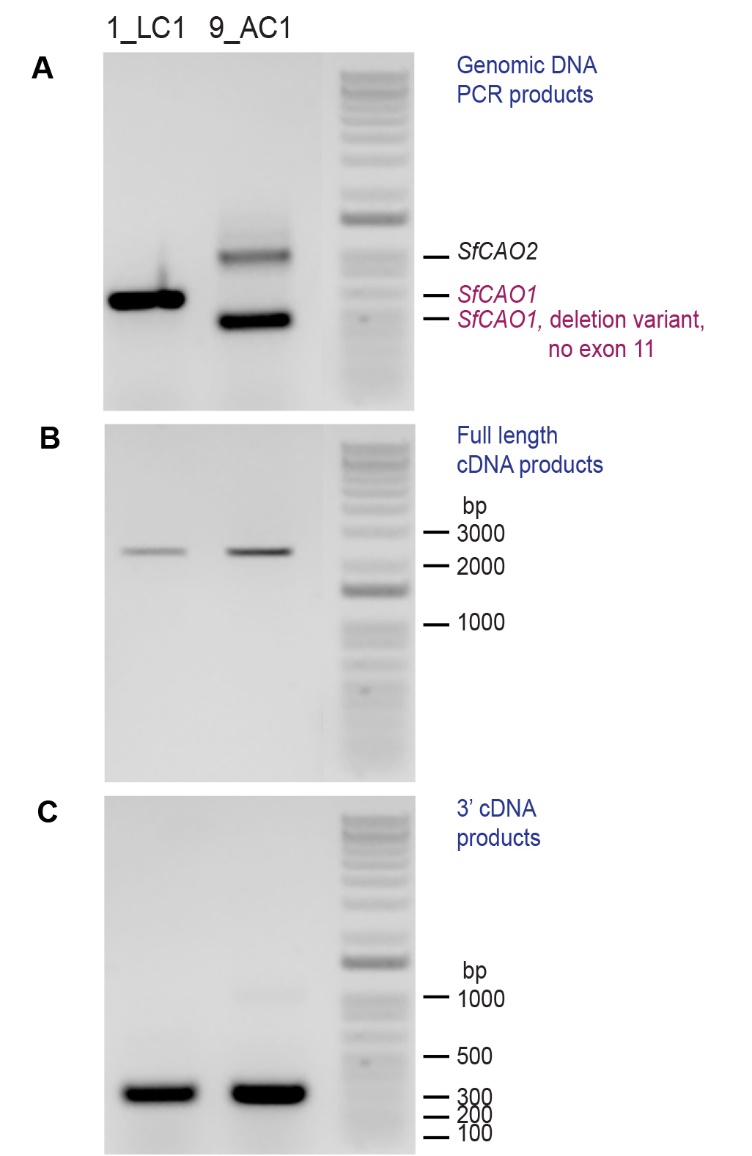


**FIGURE S12 |** Selected HPLC chromatograms showing the elution of oxymatrine and matrine in a variety of tissues. **(A)** root small AC1, **(B)** root big AC1, **(C)** TCM root, **(D)** immature seed LC4, and **(E)** dry seed. Chromatograms shown are all examples of the 10x concentrated samples (2 μL injection of 1000 μL of extract, dried, and resuspended in 30–50 μL of 30% ethanol). Chromatograms show well resolved peaks for oxymatrine (retention time (RT) = 4.61) and an unknown compound (RT=5.79). Identification of matrine (RT = 7.87) was more difficult due to its low abundance and co-elution with other compounds. UV spectra were used to confirm the presence or absence of matrine (as indicated on the chromatograms), by checking all peaks within ± 0.4 of the expected RT of matrine. The unknown peak could be sophoridine as this was the other abundant in the extract method we followed (HKCMMS, 2012), however its identity could not be verified as we do not have a commercial standard for it, nor could we find a UV spectra for it. The observed UV spectra of the unknown peak is similar to the published spectra for oxysophocarpine (data not shown). Methods such as LC-MS would allow identification of the peaks (Otterbach et al., 2019). X-axis, time. Absorbance measured at 220 nm (mAU, Y-axis).

figure next page

**
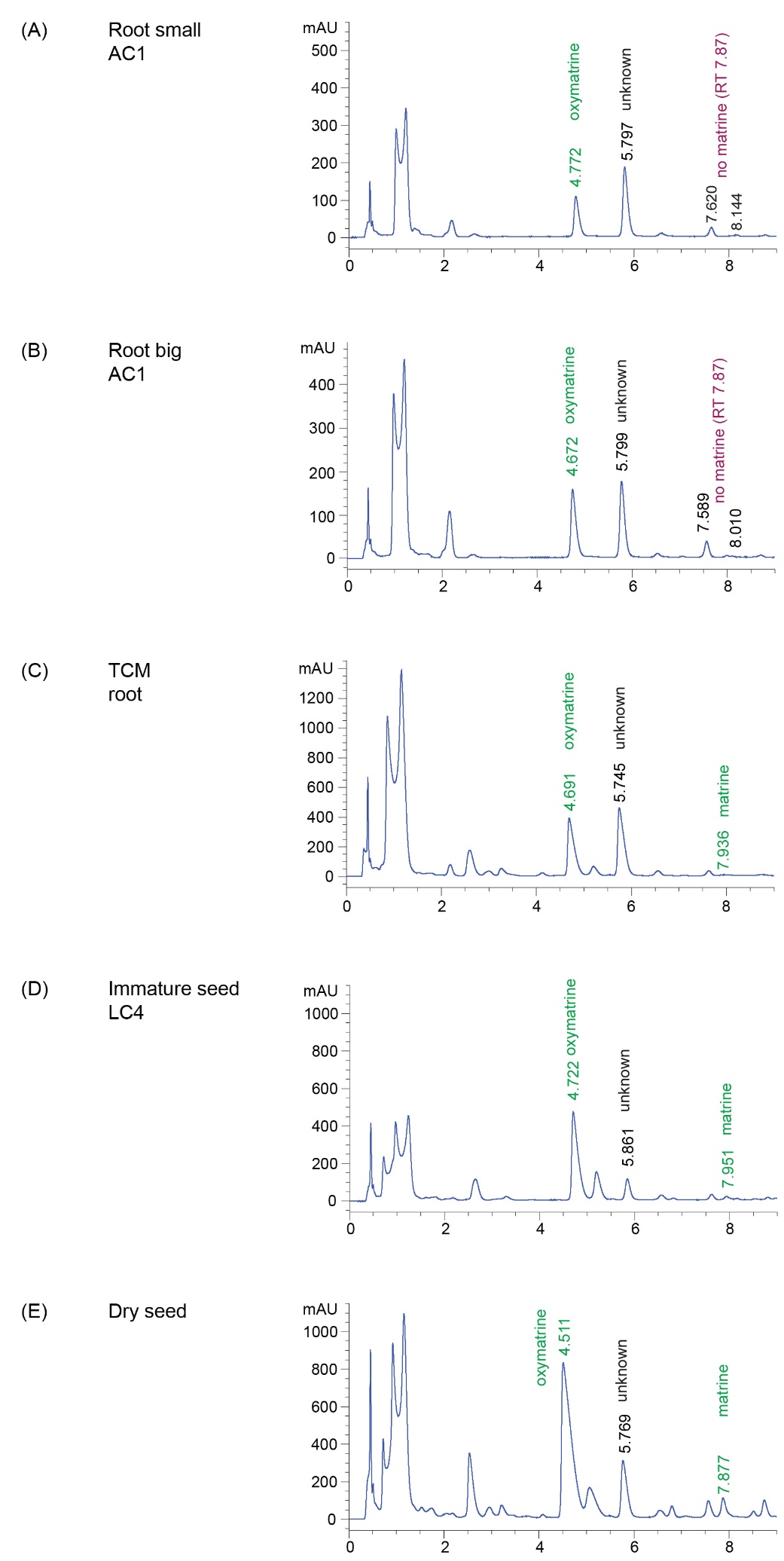
**

**References**

**Bunsupa S, Katayama K, Ikeura E, Oikawa A, Toyooka K, Saito K, Yamazaki M. 2012.** Lysine decarboxylase catalyzes the first step of quinolizidine alkaloid biosynthesis and coevolved with alkaloid production in leguminosae. *Plant Cell* **24:** 1202-1216.

**HKCMMS** (2012) Sophorae flavescentis radix. Monograph of Hong Kong Chinese Materia Medica Standards (HKCMMS) Section. Volume 4 (English) <https://www.cmro.gov.hk/hkcmms/vol4/pdf_e/Sophorae_Flavescentis_Radix_v4_e.pdf>

**Frey K, Pucker B.** 2020. Animal, fungi, and plant genome sequences harbour different non-canonical splice sites. *Cells* **9:** 458.

**Otterbach SL, Yang T, Kato L, Janfelt C, Geu-Flores F. 2019.** Quinolizidine alkaloids are transported to seeds of bitter narrow-leafed lupin. *J Exp Bot* **70:** 5799-5808.

**Yang T, Nagy I, Mancinotti D, Otterbach SL, Andersen TB, Motawia MS, Asp T, Geu-Flores F. 2017.** Transcript profiling of a bitter variety of narrow-leafed lupin to discover alkaloid biosynthetic genes. *J Exp Bot* **68:** 5527-5537.

**
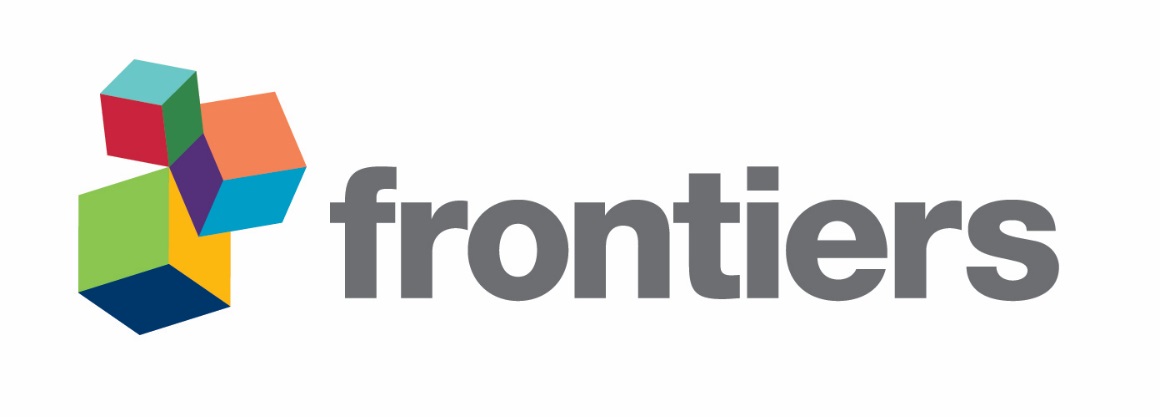
**
